# A fatty acid anabolic pathway in specialized-cells sustains a remote signal that controls egg activation in *Drosophila*

**DOI:** 10.1371/journal.pgen.1011186

**Published:** 2024-03-14

**Authors:** Mickael Poidevin, Nicolas Mazuras, Gwénaëlle Bontonou, Pierre Delamotte, Béatrice Denis, Maëlle Devilliers, Perla Akiki, Delphine Petit, Laura de Luca, Priscilla Soulie, Cynthia Gillet, Claude Wicker-Thomas, Jacques Montagne

**Affiliations:** 1 Institut for Integrative Biology of the Cell (I2BC), CNRS, Université Paris-Sud, CEA, Gif-sur-Yvette, France; 2 Laboratoire Evolution, Génomes, Comportements, Ecologie (EGCE), CNRS, IRD, Université Paris-Saclay, Gif-sur-Yvette, France; 3 Centre Médical Universitaire, Department of Cell Physiology and Metabolism, Geneva, Switzerland; College de France CNRS, FRANCE

## Abstract

Egg activation, representing the critical oocyte-to-embryo transition, provokes meiosis completion, modification of the vitelline membrane to prevent polyspermy, and translation of maternally provided mRNAs. This transition is triggered by a calcium signal induced by spermatozoon fertilization in most animal species, but not in insects. In *Drosophila melanogaster*, mature oocytes remain arrested at metaphase-I of meiosis and the calcium-dependent activation occurs while the oocyte moves through the genital tract. Here, we discovered that the oenocytes of fruitfly females are required for egg activation. Oenocytes, cells specialized in lipid-metabolism, are located beneath the abdominal cuticle. In adult flies, they synthesize the fatty acids (FAs) that are the precursors of cuticular hydrocarbons (CHCs), including pheromones. The oenocyte-targeted knockdown of a set of FA-anabolic enzymes, involved in very-long-chain fatty acid (VLCFA) synthesis, leads to a defect in egg activation. Given that some but not all of the identified enzymes are required for CHC/pheromone biogenesis, this putative VLCFA-dependent remote control may rely on an as-yet unidentified CHC or may function in parallel to CHC biogenesis. Additionally, we discovered that the most posterior ventral oenocyte cluster is in close proximity to the uterus. Since oocytes dissected from females deficient in this FA-anabolic pathway can be activated *in vitro*, this regulatory loop likely operates upstream of the calcium trigger. To our knowledge, our findings provide the first evidence that a physiological extra-genital signal remotely controls egg activation. Moreover, our study highlights a potential metabolic link between pheromone-mediated partner recognition and egg activation.

## Author summary

Efficient sexual reproduction requires gamete formation and maturation, partner mating, fecundation and the triggering of embryonic development. Deciphering the underlying physiological regulatory processes represents a major scientific challenge, crucial both for medical issues and agricultural pest control. A pivotal step in the reproductive process, known as egg activation, is the oocyte-to-embryo transition. In a wide range of metazoan species, this activation is provoked by the entry of the spermatozoon into the oocyte. Conversely, in insects, egg activation is initiated as the oocyte moves through the female genital tract, operating independently of spermatozoon entry. Here, we discovered that egg activation in *Drosophila* depends on a set of anabolic enzymes required for very-long-chain fatty acid (VLCFA) synthesis in the oenocytes. The oenocytes, abdominal cells specialized in lipid metabolism, are necessary for the biogenesis of cuticular hydrocarbons and pheromones. To our knowledge, our findings provide the first evidence that a non-genital physiological signal remotely governs egg activation. Given that pheromone-deficient *Drosophila* females attract males regardless of their species, we propose that pheromone biogenesis and this VLCFA-dependent remote signal might have co-evolved to prevent the development of hybrid individuals.

## Introduction

Sexual reproduction encompasses a complex sequence of events, including pheromone-mediated recognition, partner mating between individuals of the same species [1], gamete fusion and egg activation [2–4]. While studying the biogenesis of cuticular hydrocarbons (CHCs) and pheromones, we serendipitously discovered that CHC-deficient *Drosophila melanogaster* females were sterile because of egg activation defect, thereby highlighting a physiological link between pheromone biogenesis and embryogenesis onset.

Egg activation, representing the oocyte-to-embryo transition, is triggered by a calcium wave propagating within the oocytes [5]. Oogenesis, culminating in the formation of mature oocytes arrested at a specific meiotic stage, is controlled by numerous systemic regulatory inputs, including nutrition and hormonal signals [6–8]. The specific stage of meiosis arrest and its release upon egg activation depends on the animal species. *In contrast to oogenesis and oocyte maturation*, *egg activation*, *to our knowledge*, *has never been reported to be dependent on non-genital physiological signals*. In most metazoans, the calcium signal triggering egg activation is induced by fertilization, although, for species where egg fertilization occurs in an aquatic environment, ionic changes in Na^+^ or Mg^2+^ may also play a role in this process [9]. However, numerous studies reveal that fertilization is not required for egg activation in insects. In *Drosophila melanogaster*, oogenesis takes place in a pair of ovaries and results in stage-14 oocytes arrested at the metaphase-I of meiosis [10]. After mating, most spermatozoa are stored in the female seminal receptacle and spermathecae, and are progressively delivered to the oocytes as they reach the uterus [11–13]. Egg activation in *Drosophila* is initiated while the oocyte transits through the female genital tract [14], thereby prompting meiosis completion, *vitelline* membrane modifications to prevent polyspermy, and translation of maternally-provided mRNAs necessary for early embryogenesis [15–19]. Mature stage-14 oocytes that detach from the ovaries are swelling in the oviduct lumen, where activation initiates [20]. Interestingly, modifications of osmotic and hydrostatic pressures have been shown to induce egg activation *in vitro*, suggesting that these conditions mimic a local stimulus within the female genital tract [21]. Thus, oocyte swelling and mechanical constraints from the oviduct are postulated to induce Ca^2+^ remobilization from the endoplasmic reticulum, generating an activation wave that propagates through the entire oocyte [22,23].

*Drosophila* CHCs play a key role in waterproofing the external cuticle, and some have been identified as sexual pheromones [24]. They are produced through the decarboxylation of very-long-chain fatty acids (VLCFAs) [25]. VLCFA synthesis results from the elongation of a LCFA (long-chain fatty acid) substrate, catalyzed by an enzymatic complex [26]. This elongase complex, located at the endoplasmic reticulum, comprises four subunits: a 3-Keto-acyl-CoA-reductase (KAR, encoded by *KAR/spidey* in Drosophila), a 3-Hydroxy-acyl-CoA-dehydratase (HADC), a Trans-enoyl-CoA-reductase (TER) and the Elongase subunit (Elo) that determines the specificity of both the FA primer and the resulting VLCFA; both of which remain unidentified for most of the 20 *Drosophila* Elos [24]. LCFA synthesis is catalyzed by FASN (fatty acid synthase) from an acetyl-CoA primer [27]. Synthesis of both LCFAs and VLCFAs requires the sequential incorporation of malonyl-CoA units, whose biogenesis is catalyzed by Acetyl-CoA carboxylase (ACC) [28]. CHC synthesis chiefly takes place in the oenocytes, which are groups of cells located beneath the abdominal cuticle in the form of dorsal rows and ventral clusters [29–31]. Two of the three *Drosophila FASN* genes, *FASN2* and *FASN3*, are specifically expressed in the oenocytes [32–34]. FASN2 catalyzes the synthesis of methylated/branched(mb)FAs, utilizing an unconventional acyl-CoA primer, likely propionyl-CoA [31,33,35]. To date, neither the primer nor the final product of FASN3 has been characterized, although we previously reported that FASN3 knockdown impacts tracheal waterproofing in larvae [32].

Deciphering the physiology of sexual reproduction at the organismal level should greatly benefit from the plethora of *Drosophila* genetic tools [36]. Here, we show that a FASN3-dependent metabolic pathway operating in the oenocytes of *Drosophila* females is crucial for controlling egg activation. Thanks to RNA-interference (RNAi) knockdown targeted to the oenocytes, we have identified a set of six lipid-anabolic enzymes, indicating that an unidentified VLCFA is required for this process. Furthermore, we have shown that females deficient in these enzymes lay non-activated eggs, while their mature oocytes can still be activated *in vitro*. Since no obvious cellular defects could be observed in *Drosophila* females bearing FASN3-deficient oenocytes, our study suggests that a lipid-dependent signal acts upstream to control the genital tract triggering of egg activation. To our knowledge, our findings provide the first evidence that a non-genital physiological signal controls this critical step of female fertility.

## Results

### Identifying FA-metabolic genes required for female fertility

To investigate CHC biogenesis in *Drosophila*, we induced *UAS-RNAi* to 57 FA-metabolic genes, using the *1407-gal4* driver, which is active in oenocytes from the mid-L3 larval stage to adulthood. Surprisingly, *1407-gal4* induced knockdown of FASN3, ACC, an elongase complex subunit (KAR/spidey), a bipartite FA-transporter/acyl-CoA-ligase (FATP) and a putative short-chain-acyl-CoA-ligase (CG6432) resulted in female sterility (S1A Table and S1A Fig). The male siblings of these females were fertile (S1B Fig), indicating that a VLCFA from the oenocytes is crucial for female but not male fertility.

As previously reported [37], oenocyte knockdown of *KAR/spidey* led to oenocyte degeneration in old flies (S2A–S2F Fig). This phenotype was also observed in *1407-gal4*>*FATP-RNAi* oenocytes (S2J–S2L Fig), supporting our previous findings that FATP is closely involved in VLCFA synthesis [31]. In contrast, *1407-gal4*>*ACC-RNAi*, *1407-gal4>FASN3-RNAi* and *1407-gal4>CG6432-RNAi* oenocytes remained viable (S2G–S2I and S2M–S2R Fig). The knockdown of all these genes provoked female sterility starting from day 5 (see below), while oenocytes appeared viable at least until day 9 post-eclosion (S2D, S2G, S2J, S2M and S2P Fig), indicating that the sterile phenotype was caused by the inactivation of a specific FA-metabolic pathway, rather than due to oenocyte deficiency.

### Characterizing the metabolic pathway required for female fertility

**Fig 1:**
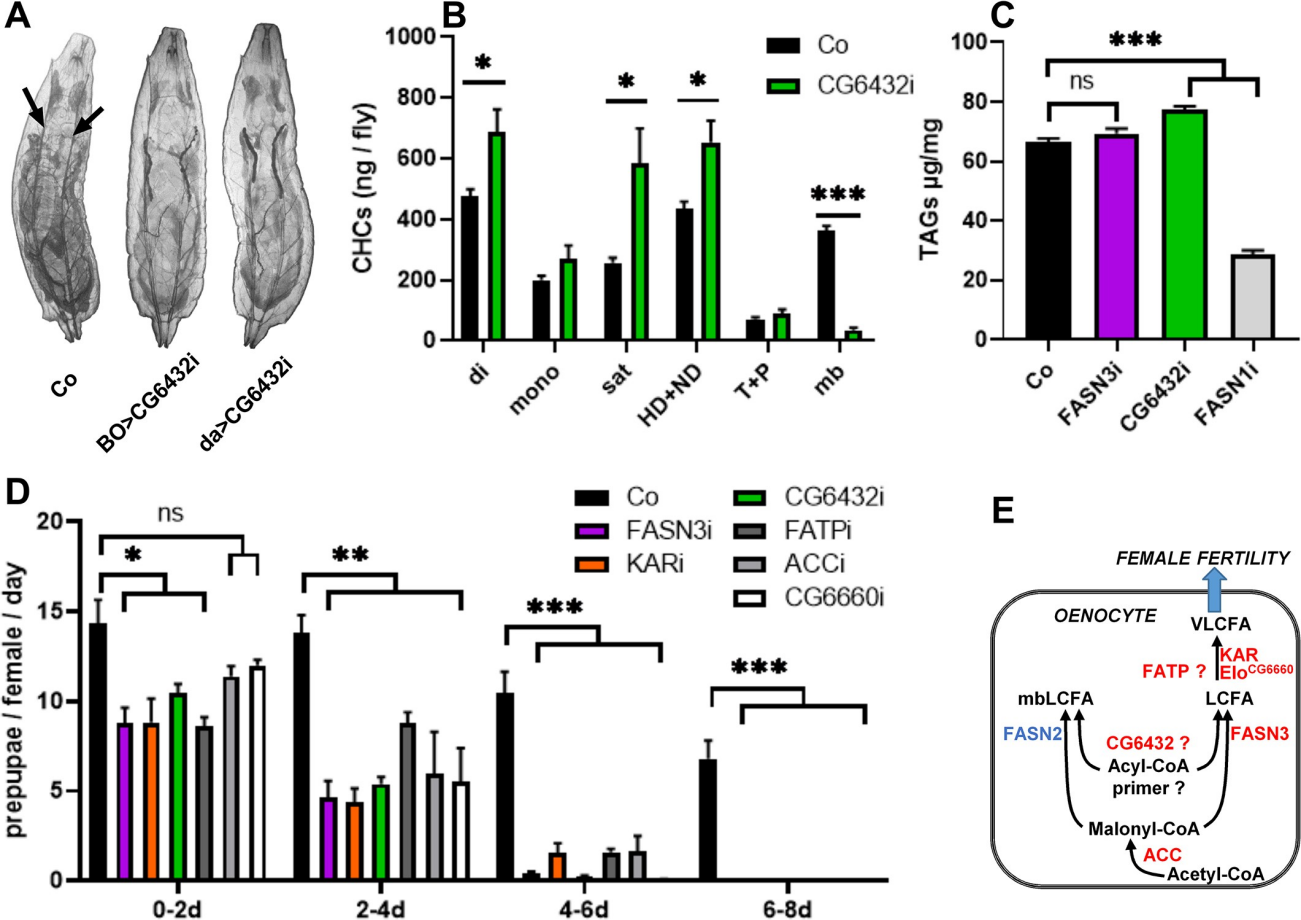
**A FA-anabolic pathway is required in the oenocytes for female fertility**: (**A**) *CG6432* knockdown using either the oenocyte-specific driver *BO-gal4* (BO) or the ubiquitous driver *daughterless-gal4* (da) induced tracheal flooding in late L2-larvae; in flooded sections, the tracheal trunks (arrows in control: Co) were hardly visible. (**B**) CHCs amounts in control (black) or *1407-gal4>CG6432-RNAi* (green) females (n = 10); Amounts of dienes (di), monoenes (mono), saturated linear (sat), pheromones (HD+ND) and mbCHCs (mb) are listed in S2 Table; note the strong reduction of mbCHCs. Bars correspond to the mean values of CHC amounts from five flies. (**C**) TAG content in 0–5 hours prepupae of the following genotypes: *Cg-gal4* control (black), *Cg-gal4>FASN3-RNAi* (purple), *Cg-gal4>CG6432-RNAi* (green) and *Cg-gal4>FASN1-RNAi* (grey). Bars correspond to the mean values of 10 replicates, each containing 10–11 prepupae. (**D**) Pupal progeny of females either control (black) or expressing *FASN3-RNAi* (purple), *KAR/spidey-RNAi* (orange), *CG6432-RNAi* (green), *FATP-RNAi* (dark grey), *ACC-RNAi* (light grey) or *elo*^*CG6660*^*-RNAi* (white). Control females are the F1 progeny from *promE-gal4* females mated with *w*^*1118*^ males, whereas oeKDs are the F1 progeny from *promE-gal4* females mated with *UAS-RNAi* males. Five 3-5-day old females were mated with five males for two days (0-2d), then, males were removed and females transferred in new tubes every second day (2-4d, 4-6d, 6-8d). Bars correspond to the mean values of 3–6 replicates and represent the numbers of pupae obtained from each egg collection. (**E**) Oenocyte anabolic pathway producing a VLCFA controlling fertility, where CG6432 is a potential acyl-CoA synthase for the primer used by FASN2/FASN3 and FATP a bipartite FA-transporter/acyl-CoA synthase potentially linked to FA elongation. Enzymes required in the oenocytes for female fertility are in red, whereas FASN2 required for mbCHCs biogenesis is in blue.

*CG6432* is hypothesized to encode a short-chain acyl-CoA ligase [38]. However, a BLAST protein alignment revealed that it contains a putative propionyl-CoA synthase domain and that its closest homologues are a short-chain fatty-acyl-CoA ligase in mice (Acss3) and an acetyl-CoA ligase in yeast (Acs1) (S3 Fig). To gain further insights into CG6432 function, we investigated its potential requirement in other FA-anabolic pathways. We previously reported that oenocyte knockdown of *ACC*, *FASN3*, *KAR/spidey* and *FATP*, using the *BO-gal4* driver that is active in the oenocytes of the embryo and early larvae, led to flooding of the tracheal system and resulted in lethality at the L2/L3 transition [32]. Similar to the effect observed with FASN3 knockdown [34], this phenotype also occurred with oenocyte (*BO-gal4*) or ubiquitous (*daughterless-gal4*) knockdown of *CG6432* (Fig 1A), indicating that the CG6432 gene product resides in the oenocyte-specific metabolic pathway controlling tracheal watertightness in larvae [32]. Furthermore, we previously reported that the *1407-gal4* induced knockdown of *ACC*, *KAR/spidey* and *FATP*, but not of *FASN3*, in adult oenocytes resulted in a severe decrease in total CHC amounts [31]. However, *1407-gal4* induced knockdown of *CG6432* did not reduce total CHC amounts in adult flies but resulted in a dramatic decrease in methylated/branched(mb)CHCs and in a concurrent increase in linear CHCs (S2 Table and Figs 1B and S4), a phenotype also seen in *1407-gal4*>*FASN2-RNAi* flies [31,33]. Finally, in contrast to fat body knockdown of *FASN1* that results in a dramatic reduction in total triacylglycerol levels, fat body knockdown (*Cg-gal4*) of *CG6432* did not impact total triacylglycerol stores (Fig 1C), highlighting its independence from FASN1. Collectively, these findings show that the enzyme encoded by *CG6432* selectively functions within the FASN2 and FASN3 but not in the FASN1 anabolic pathway.

The *1407-gal4* driver is not only active in the oenocytes, but also in several additional tissues, whereas the *BO-gal4* driver is active in the oenocytes during embryogenesis and early larval life. Therefore, in the following, we utilized the *promE-gal4* driver, which is active only in the oenocytes starting from the first larval (L1) stage [29,32]. While all the genes required in adult oenocytes for female fertility (S1A Table) are also essential in larval oenocytes for spiracle watertightness, the exception is the Elongase subunit *elo*^*CG6660*^, whose knockdown seemed to affect only the latter process [32]. Therefore, we re-evaluated *elo*^*CG6660*^ using the *promE-gal4* driver (S1B Table). Since *promE-gal4* induced knockdown (oeKD) leads to larval lethality following inactivation of each gene identified in our fertility screen [32], its activity was temporarily repressed until early metamorphosis using the thermo-sensitive Gal4 inhibitor, Gal80^ts^. Under this protocol, RNAi expression was induced by a temperature shift to 27°C. Emerging females were maintained at 27°C, mated 3 to 5 days after eclosion and transferred to new vials every second day. Compared to controls, females expressing any of the RNAis, including *elo*^*CG6660*^*-RNAi*, showed a marked decrease in fertility, which quickly dropped to complete sterility (Fig 1D). Due to variable RNAi efficiency across different targeted sequences, we employed additional RNAi lines to *CG6432*, *elo*^*CG6660*^, *FASN3*, *KAR/spidey* and *FATP*. All these lines, when driven by the *promE-gal4* driver, induced female sterility (S5A Fig). In sum, six FA-anabolic enzymes operating in the oenocytes are required for female fertility (Fig 1E): ACC, catalyzing malonyl-CoA synthesis [28]; FASN3, one of the three *Drosophila* FASN enzymes [34]; CG6432, potentially a short-chain fatty-acyl-CoA ligase or an acetyl-CoA ligase; FATP, a bipartite FA-transporter/acyl-CoA synthase previously suspected to be involved in VLCFA synthesis [31]; KAR/spidey and Elo^CG6660^, both components of the elongase complex as a common and a specific subunit, respectively [26]. Given that neither *1407-gal4*>*FASN3-RNAi* [31], nor *1407-gal4*>*CG6432-RNAi* (Fig 1B and S2 Table), affected total CHCs amounts, the sterility phenotype is more likely due to the absence of a specific VLCFA rather than a general VLCFA deficiency. Additionally, except a few minor variations, the CHC profile of *1407-gal4*>*elo*^*CG6660*^*-RNAi* flies did not dramatically differ from that of controls (S3 Table).

**Fig 2:**
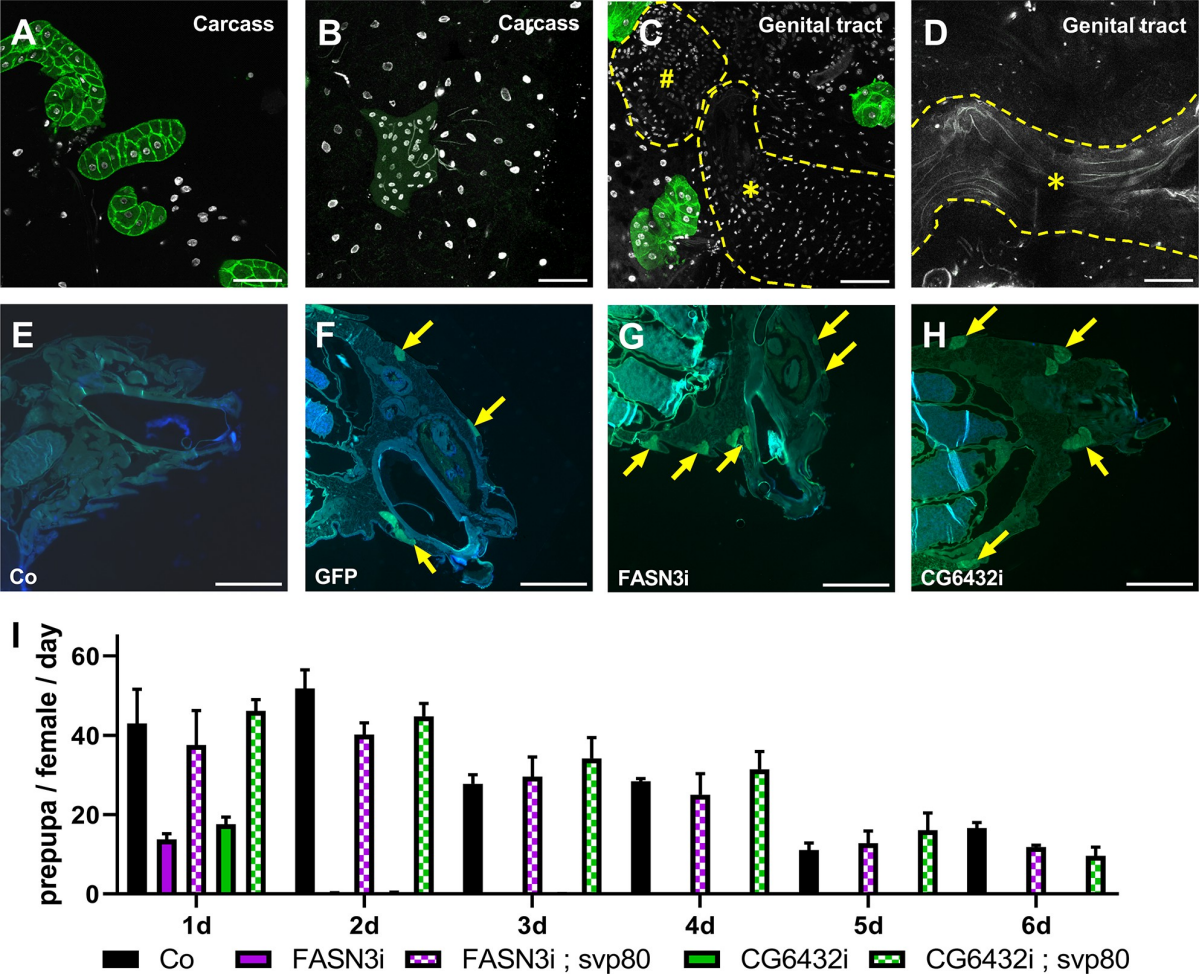
**Oenocyte specificity of the *promE-gal4* driver:** (**A-B**) abdominal cuticle with fat body and oenocytes of *promE-gal4>UAS-GFP* females with (B) or without (A) the *svp-gal80* transgene, which strongly suppresses the oenocyte-specific GFP expression. (**C-D**) Dissected genital tract of *promE-gal4>UAS-GFP* females with (D) or without (C) the *svp-gal80* transgene. Note the lack of GFP expression in the genital tract, even though GFP acquisition signal was increased compare to that of Fig 2A and 2B, and that the most posterior oenocytes can remain tightly linked to the uterus after dissection (C). (*) Uterus; (**#**) seminal receptacle. (**E-G**) Transversal sections of embedded abdomens of *promE-gal4* 10-day old females either control (E), expressing two *UAS-GFP* transgenes (F) or one *UAS-GFP* transgene together with *FASN3-RNAi* (G) or *CG6432-RNAi* (H); unspecific auto-fluorescence likely due to lipid accumulation or tissue folding can be observed. (**I**) Pupal progeny of *promE-gal4>FASN3-RNAi* (purple) and *promE-gal4>CG6432-RNAi* (green) females in combination with (dotted colors) or without (plain colors) the *svp-gal80* transgene. Three females were mated with three males for one day, then, males were removed and females transferred in new tubes every day over a 6-day period. Bars correspond to the mean values of 5 replicates and represent the numbers of pupae obtained from each egg collection.

When using the *promE-gal4* driver, we observed total female sterility beginning on day 8 post-eclosion (Fig 1D and 2E). In contrast, *1407-gal4>UAS-RNAi* females appeared to be fully sterile when egg collection began 5 to 7 days after adult eclosion (S1A Fig). However, when egg collection began 4 days after adult eclosion, *1407-gal4>UAS-FASN3-RNAi* and, to a lesser extent, *1407-gal4>UAS-CG6432-RNAi* females produced a few progeny (S5B Fig). Similarly, when using the *promE-gal4* driver, and if the temperature shift to 27°C occurred at L2/L3 transition, *FASN3-* and *CG6432-*oeKD allowed the survival of a few females that then produced offspring during their early adult life, while developing a totally sterile phenotype by day 5 post-eclosion (S5C Fig). The sterile phenotype cannot be attributed to a non-specific effect of the *promE-gal4* driver, since oeKD of CPT1 ─the gatekeeper for mitochondrial FA β-oxidation─ and of CG3415 ─a peroxisomal multifunctional enzyme─ did not result in female sterility (S5C Fig). Moreover, considering the *1407-gal4* activity in several tissues, to confirm the oenocyte specificity of the sterile phenotype, we used the *svp-gal80* transgene to suppress Gal4 activity in the oenocytes [39]. In this setting, the sterile phenotype of *1407-gal4>UAS-FASN3-RNAi* and *1407-gal4>UAS-CG6432-RNAi* females was completely rescued by the *svp-gal80* transgene (S5D Fig). Finally, to rule out any leaky activity in the germline, we employed the *nanos-gal4* driver to direct *UAS-FASN3-RNAi* and *UAS-CG6432-RNAi*, and monitor the pupal progeny of 6-day old females. The pupal progeny from the eggs laid during the subsequent 10 days showed no significant difference between control and knockdown females (S6 Fig), thus excluding any potential germline effect and conclusively confirming the oenocyte specificity of the sterile phenotype.

### Analyzing oocyte fertility

We focused on *FASN3* and *CG6432*, since their knockdown does not affect oenocyte viability nor total CHC amounts. First, to verify oenocyte specificity, we used the *svp-gal80* transgene to suppress Gal4 activity in the oenocytes [39]. In *promE-gal4*>*UAS-GFP* adult females, GFP expression was not detected in the genital tract, but was exclusive to oenocytes and strongly suppressed by the *svp-gal80* transgene (Fig 2A–2D). Surprisingly, we noticed that the most posterior oenocytes sometimes remained tightly associated with the uterus and the seminal receptacle after dissection (Fig 2C). Therefore, we examined transversal sections of non-dissected abdomens of *promE-gal4* females either control or directing two *UAS-GFP* transgenes (Fig 2E and 2F). In this setting, GFP-positive oenocytes were found underneath the abdominal cuticle, while the most posterior ventral ones appeared apposed to the uterus (compare Fig 2E and 2F). Importantly, both dorsal and ventral oenocytes were observed in the abdomen of *FASN3-* and *CG6432-*oeKD 10-day old females also expressing one GFP transgene (Fig 2G and 2H). In addition, the sterile phenotype of *FASN3* and *CG6432-*oeKD was fully rescued by the *svp-gal80* transgene (Fig 2I). Taken together, these results not only rule out leaky expression of the *promE-gal4* driver in the female genital tract, but also highlights that a cluster of posterior oenocytes is in close proximity to the uterus and demonstrate that the oenocytes synthesize a VLCFA required for female fertility.

**Fig 3:**
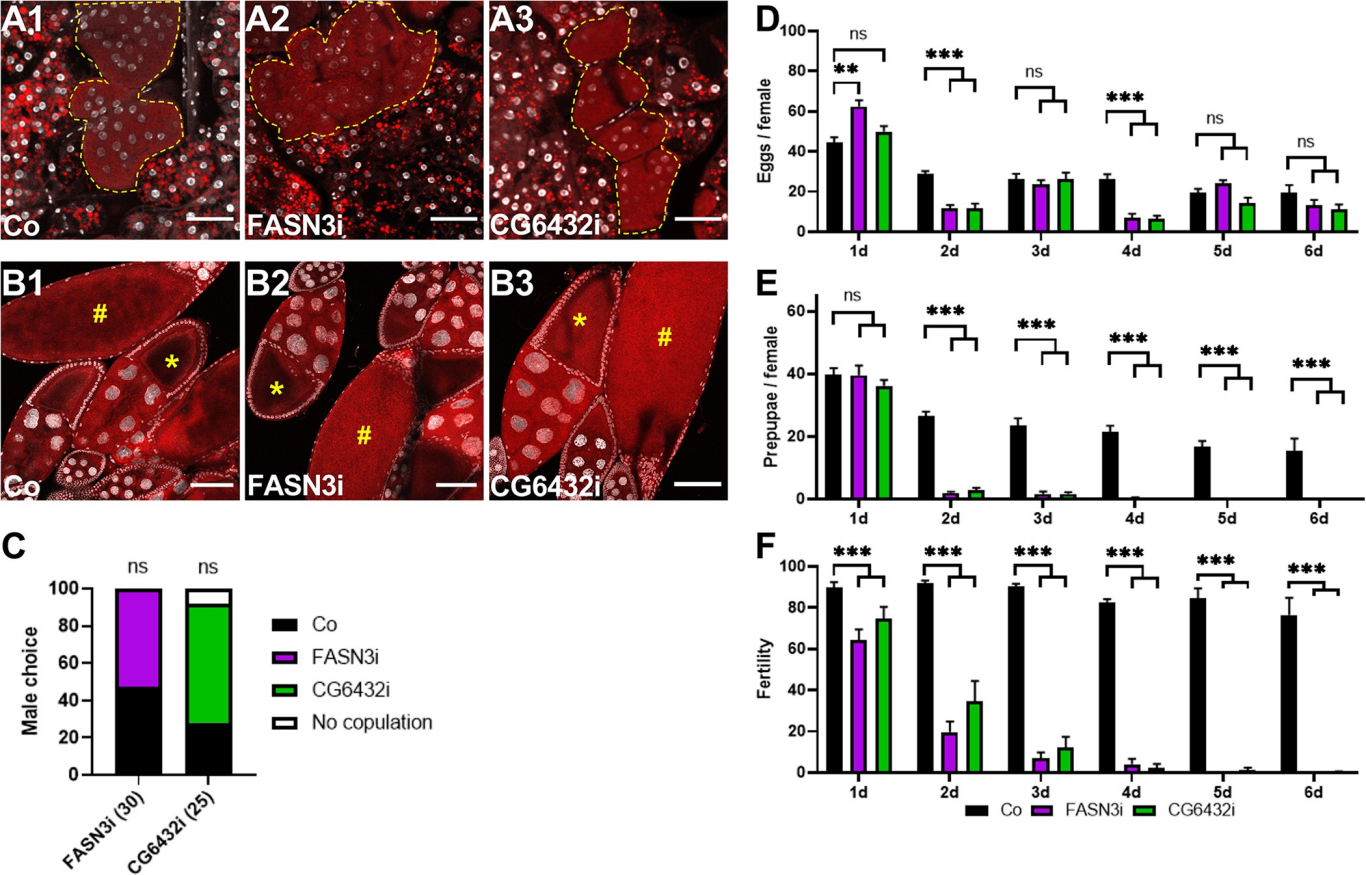
**Oogenesis and mating:** (**A-B**) Oenocytes (yellow dotted-line in A1-3) and stage 9–10 (*) and late (**#**) egg chambers (B1-3) of *promE-gal4* females either control (A1, B1), or directing *FASN3-RNAi* (A2, B2) or *CG6432-RNAi* (A3, B3); tissues were dissected 10 days after mating; lipids and nuclei were labelled with Nile Red (red) and DAPI (silver), respectively; scale bars: 40μm (A1-3) and 100μm (B1-3). (**C**) Mating choice of single wild-type males in the presence of two females, one control and one expressing either *FASN3-RNAi* (purple) or *CG6432-RNAi* (green); bars represent the percentage of copulation with control (black) or RNAi-expressing (purple and green) females; males tend to prefer *CG6432-RNAi* females although not significantly; (N) represents the numbers of choice tests. (**D-F**) eggs (D) and pupae (E) from *promE-gal4* females either control (black) or expressing *FASN3-RNAi* (purple) or *CG6432-RNAi* (green); three 3–5 day old females were mated with three males for one day, then, males were removed and females transferred in new tubes every day over a 6-day period; index of fertility (F) were evaluated as the ratio of prepupae to eggs (total numbers). Bars correspond to the mean values of 9–10 replicates and represent the numbers of eggs (D) and pupae (E) obtained from each egg collection.

Next, we analyzed oenocytes and ovaries of *FASN3-* and *CG6432-*oeKD 10 days after mating, but did not find any noticeable defects (Fig 3A and 3B). Further, in mating assays, single wild-type males showed no significant preference when given a choice between a control female and either a *FASN3-* or *CG6432-*oeKD female (Fig 3C). We also monitored the number of eggs laid by females. Control, *FASN3-* or *CG6432-*oeKD females laid high amounts of eggs the day after insemination (Fig 3D); this number decreased the following days, although this effect was more pronounced for *FASN3-* and *CG6432-*oeKD compared to control females (Fig 3D). However, the number of eggs that eventually developed to pupae in the *FASN3-* and *CG6432-*oeKD conditions dropped more dramatically than the number of eggs laid, as compared to controls (Fig 3E and 3F). Therefore, the sterile phenotype of females bearing metabolic-deficient oenocytes is not attributable to partner mating or egg laying defects.

### Oenocytes do not influence sperm delivery to the oocytes

**Fig 4:**
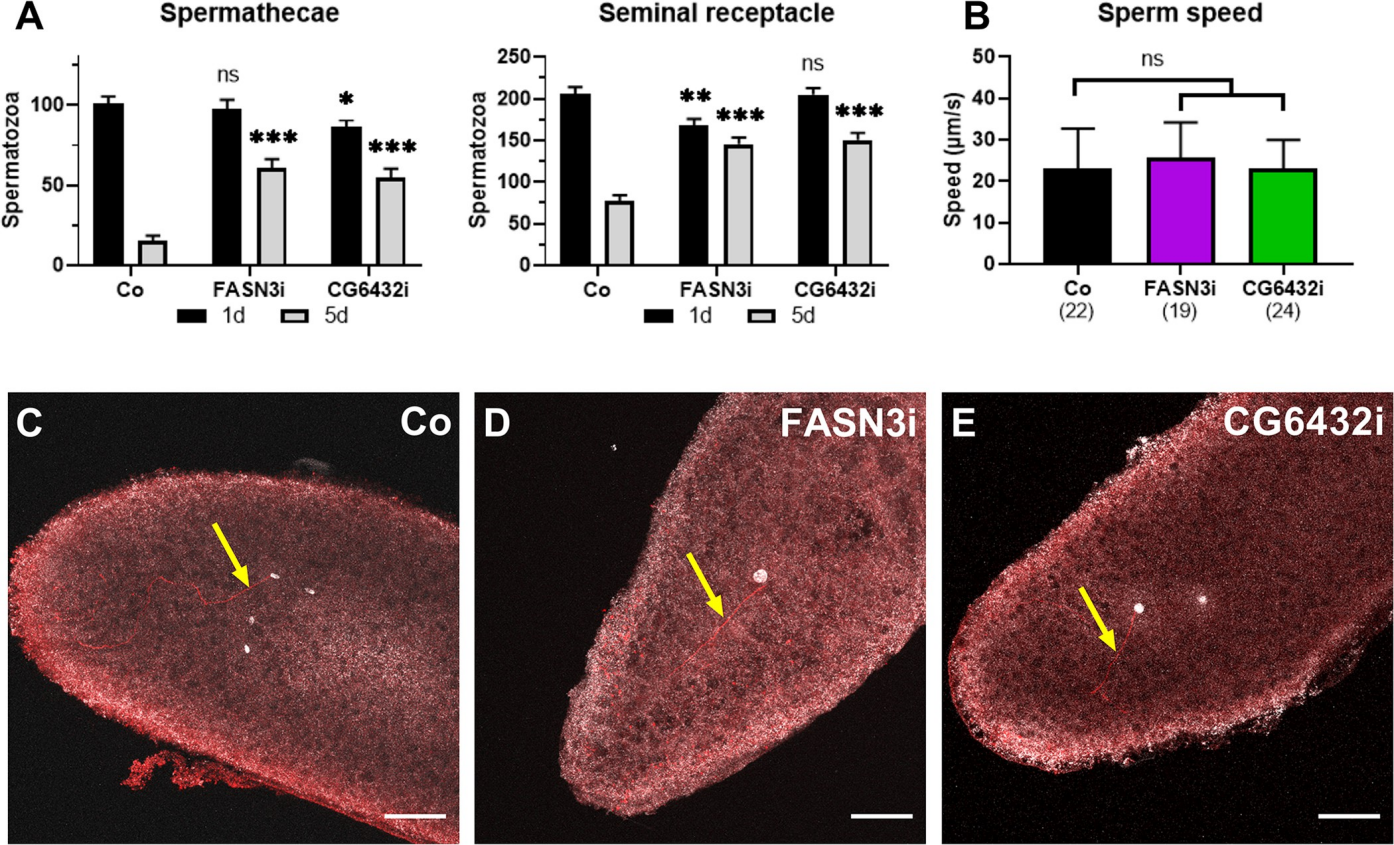
**Sperm activity:** (**A**) Numbers of spermatozoa in the spermathecae and the seminal receptacle of *promE-gal4* females either control (Co) or directing *FASN3-RNAi* or *CG6432-RNAi*, one day (black) and five days (grey) after mating; 3-5-day old females were mated with *P[ProtB-DsRed-monomer*,*w+]* males to label the spermatozoa; each bar corresponds to the mean values from 26 to 32 females (n>25). (**B**) Movement speed of *P[ProtB-DsRed-monomer*,*w+]* spermatozoa in the seminal receptacle of *promE-gal4* females either control (black), or directing *FASN3-RNAi* (purple) or *CG6432-RNAi* (green). (**C**-**E**) Eggs laid by *promE-gal4* 10-day old fertilized females either control (C), or directing *FASN3-RNAi* (D) or *CG6432-RNAi* (E); eggs were collected for 40 min, nuclei were labeled with DAPI (silver) and sperm flagella (arrows) with an anti-acetylated-tubulin (red); scale bars: 40μm.

We investigated potential defects in the spermatozoa, which after copulation, are stored within the seminal receptacle and spermathecae of the females, and progressively delivered to the oocytes that transit through the uterus [12]. In controls, the sperm number in the storage organs gradually declined after mating as females lay eggs (Fig 4A). The day after mating, the sperm number in the storage organs of *FASN3-* and *CG6432-*oeKD females was similar to that of controls (Fig 4A). Surprisingly, during the following days, this number decreased less in *FASN3-* and *CG6432-*oeKD females than in controls (Fig 4A). We also monitored sperm motility in the seminal receptacle but detected no difference in sperm speed between controls and *FASN3-* or *CG6432-*oeKD females (Fig 4B and S1–S3 movies). Moreover, confocal microscopy analysis revealed that the eggs laid by control, *FASN3-* and *CG6432-*oeKD 10-day old females were fertilized, as evidenced by the presence of sperm flagella (Fig 4C–4E). These findings indicate that, despite increased sperm retention in storage organs, the fertility defect is not due to impaired sperm entry into the oocyte.

### Oenocytes control egg activation

**Fig 5:**
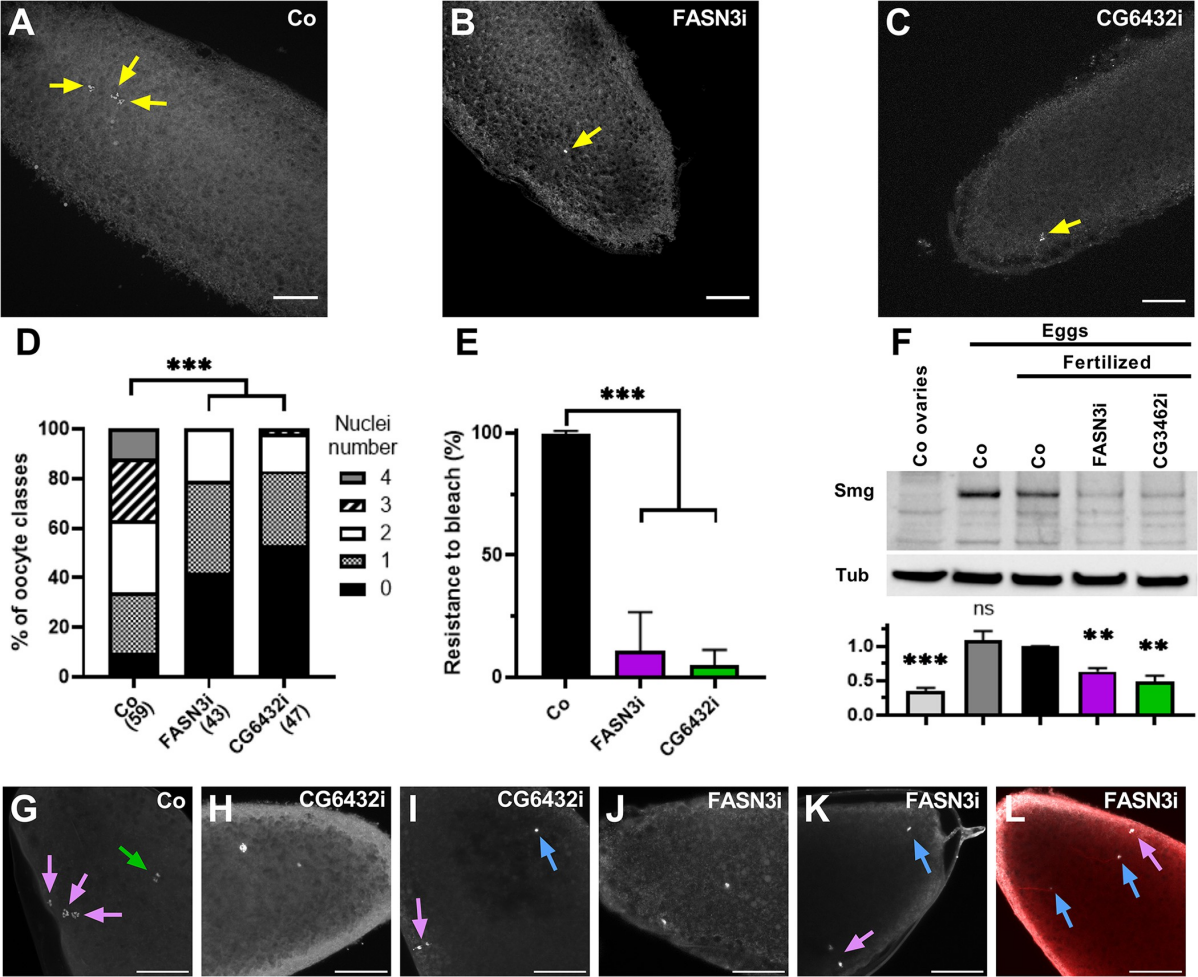
**Egg activation defect:** (**A**-**C**) Eggs laid by *promE-gal4* 10-day old virgin females either control (A), or directing *FASN3-RNAi* (B) or *CG6432-RNAi* (C); eggs were collected for 2hrs and nuclei labeled with DAPI (silver); scale bars: 40μm. (**D**) Numbers of nuclei counted in imaged eggs. (**E**) Resistance to bleach lysis of eggs laid by *promE-gal4* fertilized females either control (black), or directing *FASN3-RNAi* (purple) or *CG6432-RNAi* (green); bars correspond to the mean values from 27 independent tests, each containing 25 eggs per genotype. (**F**) Western-blotting to Smaug from protein extracts of dissected ovaries or eggs laid by *promE-gal4* 10-day old females either virgin or fertilized; ovaries and unfertilized eggs were from control females, fertilized eggs were from females either control or directing *FASN3-RNAi* or *CG6432-RNAi*. The bottom graph compares the means of four independent blots, where the band intensity of Smaug was normalized to that of the tubulin loading-control. (**G-L**) Confocal imaging of eggs laid by *promE-gal4* 10-day old fertilized females either control (G: metaphase-II first zygotic division), or directing *CG6432-RNAi* (two nuclei visible in H, three in I) or *FASN3-RNAi* (two nuclei visible in J, three in K and L; flagella connect two nuclei in L); nuclei are stained with DAPI (silver), flagella (L) with an anti-acetylated-tubulin (red). Arrows indicate nuclei likely of female (red) or male (bleu) origin. Collection for control and mutant eggs last 15 min and 1h, respectively; scale bars: 40 μm. Bleach dechorionation resulted in dramatic lysis of most eggs laid by *FASN3-* or *CG6432-*oeKD females.

A fertilized egg contains potentially five nuclei: the sperm and oocyte pronuclei, plus three polar bodies. However, upon examining the presence of sperm flagella, we noticed an evident defect in the number of nuclei in eggs laid by *FASN3-* and *CG6432-*oeKD 10-day old females (Fig 4C–4E). Consequently, we counted the number of nuclei in eggs laid by 10-day old virgin females and observed that this number in control eggs varied from zero to four, potentially because some nuclei are not stained or not visible (Fig 5A and 5D). Nevertheless, the number of nuclei in the eggs laid by *FASN3-* and *CG6432-*oeKD females was significantly lower than in controls (Fig 5B, 5C and 5D). The production of the three polar bodies is a result of meiosis completion [40], suggesting that this process may not be fully functional in *FASN3-* and *CG6432-*oeKD females. Meiosis completion is triggered by egg activation, which also induces hardening of the vitelline membrane and translation of maternally provided mRNAs [15]. Notably, the vitelline membrane of activated eggs makes them resistant to bleach [21]. Importantly, we observed that the fertilized eggs laid by *FASN3-* and *CG6432-*oeKD 10-day old females showed significantly reduced resistance to bleach treatment compared to control eggs (Fig 5E). Next, we analyzed Smaug, a protein encoded by a maternally provided mRNA, whose translation is induced by egg activation [21,41]. Western-blot analysis revealed that Smaug levels were considerably lower in eggs laid by *FASN3-* and *CG6432-*oeKD 10-day old females than in control eggs (Fig 5F). To further characterize the meiotic arrest, we compared the eggs laid by *FASN3-* and *CG6432-*oeKD females with those laid by control females, relying on previous publications that reported wild type and mutant phenotypes [42–47]. While eggs laid by control females were collected every 15 min, egg collections from *FASN3-* and *CG6432-*oeKD females lasted for 1hr. This prolonged period was necessary to confirm meiosis arrest and to obtain enough material, since bleach-dechorionation provoked a dramatic loss of mutant eggs. In control eggs, we observed various stages of early embryonic division, including the typical display of male and female pronuclei and polar bodies (e.g. metaphase of first cycle embryonic division in Fig 5G). In contrast, the eggs laid by *FASN3-* and *CG6432-*oeKD 10-day old females did not undergo early embryonic division, with most containing two (Fig 5H and 5J) or three nuclei (Fig 5I and 5K), one of which was likely the male pronucleus, while those presumed to be of female origin appeared arrested between anaphase-I and metaphase-II of meiosis. However, in the case where meiosis was arrested at metaphase-II, the axis of nuclei was positioned perpendicular to the cortex (Fig 5I and 5K), suggesting that spindle rotation had occurred. In addition, the presence of sperm flagella suggested that eggs laid by *FASN3-* and *CG6432-*oeKD females might contain more than one male pronucleus (Fig 5L). These findings indicate that eggs laid by *FASN3-* and *CG6432-*oeKD females exhibit both vitelline membrane defects and incomplete meiosis.

**Fig 6:**
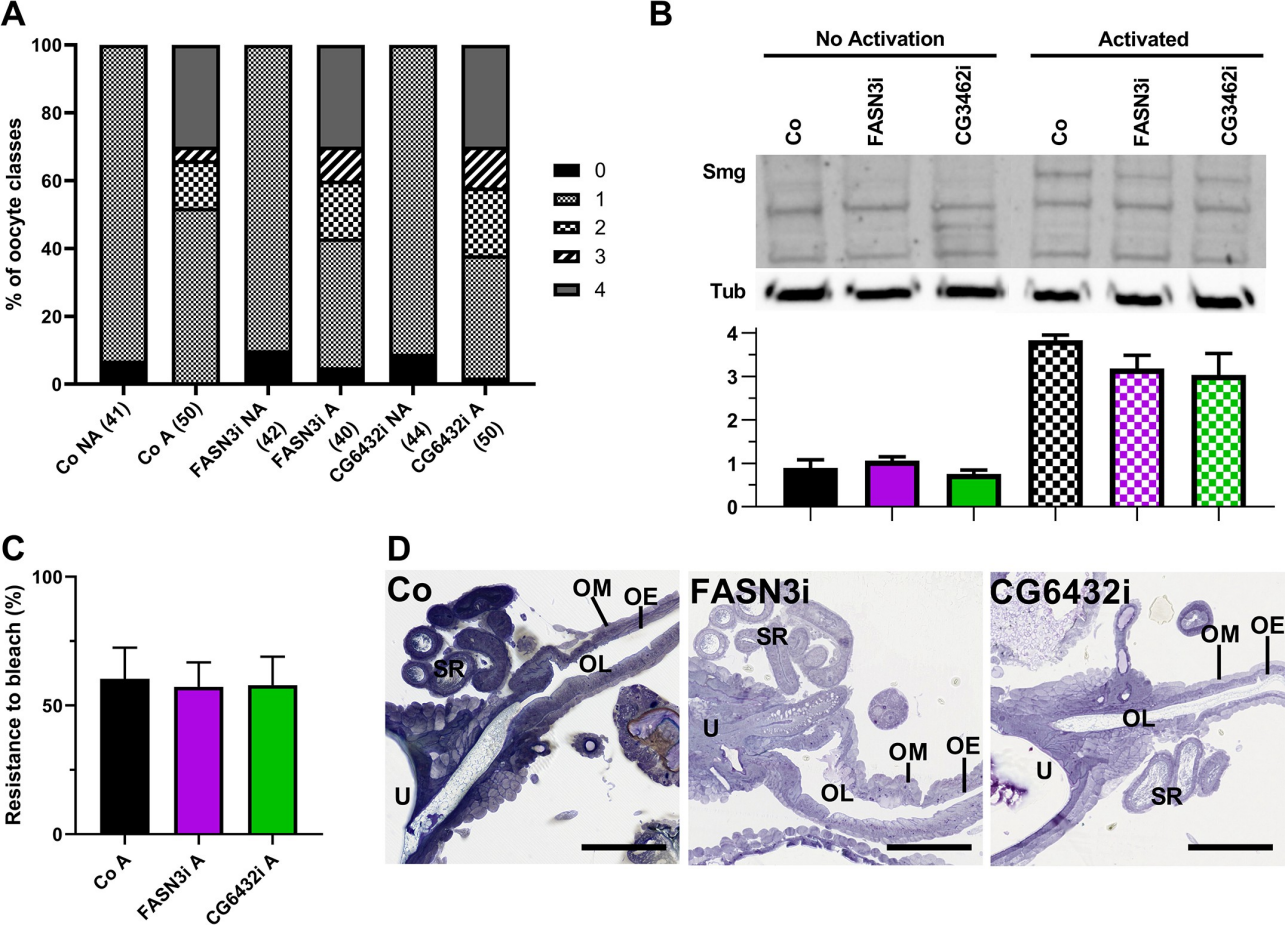
**Oocyte activation:** (**A**) Numbers of nuclei counted in stage-14 oocytes dissected from *promE-gal4* 10-day old females either control, or directing *FASN3-RNAi* or *CG6432-RNAi*. Oocytes were fixed, labeled with DAPI and imaged as in Fig 5A–5D; A: activated; NA: not activated. (**B**) Western-blotting to Smaug from protein extracts of stage-14 oocytes dissected from *promE-gal4* 10-day old females either control, or directing *FASN3-RNAi* or *CG6432-RNAi*. The bottom graph compares the means of three independent blots, where the band intensity of Smaug was normalized to that of the tubulin loading-control. (**C**) Resistance to bleach lysis of *in vitro* activated stage-14 oocytes dissected from *promE-gal4* 10-day old females either control, or directing *FASN3-RNAi* or *CG6432-RNAi*. Chlorax concentration and treatment duration was established for roughly 50% oocyte lysis in controls; bars correspond to the mean values of the percentage of bleach-resistant oocytes from 4 independent experiments. (**D**) Common oviducts dissected from *promE-gal4* 10-day old females either control, or directing *FASN3-RNAi* or *CG6432-RNAi*. OL: oviduct lumen; OE: oviduct epithelium; OM: oviduct muscles; SR: seminal receptacle; U: uterus. Scale bars: 75μm.

Our findings indicate that eggs laid by *FASN3-* and *CG6432-*oeKD 10-day old females fail to activate. This phenotype may stem from a flaw in either the activation process *per se* or an earlier step of oogenesis, rendering the eggs unresponsive to the activation signal as they move through the genital tract. To investigate this issue, we dissected stage-14 oocytes and tested their ability for *in vitro* activation using a hypotonic buffer [40,48]. Untreated stage-14 oocytes from control and *FASN3-* and *CG6432-*oeKD 10-day old females contained only one nucleus, whereas the nuclei number for all samples significantly increased after hypotonic treatment (Fig 6A). Consistently, the hypotonic treatment of stage-14 oocytes from control and *FASN3-* and *CG6432-*oeKD 10-day old females prompted the expression of Smaug (Fig 6B) and resistance to bleach treatment (Fig 6C). Taken together, these findings demonstrate that the eggs laid by *FASN3-* and *CG6432-*oeKD 10-day old females are unactivated yet capable of *in vitro* activation, indicating that the oenocytes control egg activation *per se*, rather than an oogenesis stage. *In vitro* activated stage-14 oocytes are not subjected to fertilization by spermatozoa and thus, do not proceed with embryogenesis. To determine whether eggs laid by *FASN3-* and *CG6432-*oeKD 10-day old females could developed upon *in vitro* activation, we performed a mild dechorionation treatment followed by an osmotic choc and monitored pupae formation. In this setting, none of the eggs laid by *FASN3-* and *CG6432-*oeKD fertilized females developed to pupae, whereas about half of the control eggs progressed to pupae irrespective of the osmotic choc treatment (S7 Fig), indicating that *in vitro* activation fails to promote development after egg laying. Given that egg activation is initiated while the oocyte transits through the oviduct, we dissected the female genital tracts and performed histological analysis to examine tissue organization of the oviduct. However, we did not observed any noticeable differences between the genital tracts of control and *FASN3-* or *CG6432-*oeKD 10-day old female (Fig 6D). In summary, our findings suggest that a VLCFA synthesized in the oenocytes either directly or indirectly acts on the female genital tract to trigger egg activation, while not altering the overall structure of the oviduct epithelium.

## Discussion

To our knowledge, our study provides the first evidence that egg activation can be controlled by a physiological non-genital signal. We show that a VLCFA synthesized in the oenocytes is required for this activation in *Drosophila melanogaster* females. Eggs laid by *Drosophila* females bearing dysfunctional oenocytes are not activated, while their stage-14 oocytes can be activated *in vitro*. These findings indicate that the egg activation flaw results from a defect in an upstream physiological lipid-dependent signal, but not in the oocyte itself.

We have identified six enzymes, ─ACC, FASN3, CG6432, FATP, KAR/spidey and Elo^CG6660^─ which act in the oenocytes to control egg activation, all exhibiting strong homologies to FA-anabolic enzymes (Fig 1E). Although their *in vitro* enzymatic activities have not been formally demonstrated, various pieces of evidence indicate that this metabolic pathway catalyzes VLCFA biogenesis. First, oenocyte knockdown of ACC, FATP, KAR/spidey results in the lack of CHCs, the precursors of which being VLCFAs [31,32]. Second, fat body knockdown of ACC, results in a severe reduction in TAG storage [31,32]. Third, oenocyte knockdown of CG6432 results in the lack of mbCHCs, similar to FASN2 knockdown [31,32]. Here, we found that CG6432 is also required for the synthesis of metabolites reliant on FASN3, the other oenocyte-specific FASN enzyme [33,34]. The best homologues of CG6432 are a short-chain fatty-acyl-CoA ligase in mouse and an acetyl-CoA ligase in yeast. Although the acyl-CoA primer for mbLCFA synthesis catalyzed by FASN2 is likely a propionyl-CoA [35], the one used by FASN3 remains unidentified. Since CG6432 contains a putative propionyl-CoA synthase domain, it is tempting to speculate that it catalyzes the synthesis of the FASN2 and FASN3 primers. Fourth, knockdowns of KAR/spidey and Elo^CG6660^, which are subunits of the elongase complex comprising four distinct subunits [26], indicate that the eventual product is a VLCFA. KAR is a common reductase subunit, whereas the Elongase subunit (Elo) determines the specificity to FA primer usage and to the VLCFA produced; both of which remain unidentified for Elo^CG6660^, as for most of the 20 *Drosophila* Elos [24]. In addition, the knockdown of FATP, which encodes a bipartite FA-transporter/acyl-CoA synthase, impacts oenocyte long-term survival similarly to KAR/spidey knockdown, reinforcing our previous suggestion that FATP is closely linked to VLCFA synthesis [31]. These six enzymes outline a metabolic pathway (Fig 1E) that is also required in larval oenocytes for spiracle watertightness [32], suggesting that a FASN3/Elo^CG6660^-dependent VLCFA exerts remote control from the oenocytes. Importantly, both processes likely depend on a specific VLCFA, rather than overall VLCFA synthesis, since knockdown of FASN3, CG6432 or Elo^CG6660^ does not affect the total CHC amounts [31,32]. Determining whether the FASN3/Elo^CG6660^-metabolic axis produces one particular VLCFA or a few ones, and whether it effluxes from the oenocytes to directly signal to the target tissues remains an open question. Unraveling the nature of this specific VLCFA and any putative-derived signal will require extensive future research, encompassing lipidomics and the development of functional tests to challenge the specific activity of potential candidates.

In most metazoan species, egg activation is typically induced by spermatozoon entry into the oocyte [9,17]. In insects, egg activation does not require spermatozoon entry [15], congruent with the occurrence of parthenogenesis in several species. In *Drosophila*, it has been shown that the activation initiates in the oviduct and proceeds while the oocyte moves through the genital tract [14,18,20,49]. Since stage-14 oocytes from *Drosophila* females with dysfunctional oenocytes can be activated *in vitro*, the lipid-dependent signal is likely to act upstream of the genital tract inducer, rather than directly on the oocyte, even though no visible defect in the oviduct epithelium could be detected. Egg activation induces several changes, including meiosis completion and modification of the vitelline membrane to prevent polyspermy. Eggs laid by *FASN3-* and *CG6432-*oeKD females show arrest between anaphase-I and metaphase-II of meiosis. These defects partially phenocopy those observed in Calcipressin *sarah* mutants [46], with the exception of the spindle rotation that apparently proceeds in the eggs laid by *FASN3-* and *CG6432-*oeKD females. In *Drosophila*, the release of spermatozoa from spermatheca and seminal receptacle takes place at the uterus anterior region [50] and the spermatozoon enters the oocyte via the micropyle, an extension of the anterior eggshell [51]. Therefore, changes in the vitelline membrane ─particularly in the micropylar region─ to prevent further spermatozoon entry cannot occur earlier than when the oocyte has fully entered the uterus. It remains unclear whether egg activation relies solely on initiation within the oviduct or requires additional inputs from the uterus. Intriguingly, we observed that the most posterior oenocytes are in close proximity to the uterus and the seminal receptacle. Furthermore, beyond the egg activation defect, we observed sperm retention in the storage organs, while eggs laid by *FASN3-* and *CG6432-*oeKD sterile females were fertilized. The vitelline membrane defect certainly increases the likelihood of sperm entry, evidenced by the occurrence of polyspermy. Additional research is needed to determine whether egg activation requires signals from both the oviduct and the uterus, and how this relates to sperm delivery.

At first glance, it is surprising that a VLCFA synthesized via a metabolic pathway parallel to that responsible for pheromone biogenesis, remotely controls oocyte activation. Notably, it has been shown that *Drosophila melanogaster* females devoid of oenocytes are attractive to males from other species, including *D*. *simulans*, *D*. *yakuba* and *D*. *erecta* [29]. These females are also more attractive to wild type *Drosophila melanogaster* males, evidenced by a reduced delay for copulation with oenocyte-deficient females. The increased attractiveness is believed to stem from carboxyl-methylated FAs produced by *Drosophila* females, since a shortened copulation delay does not occur with males deficient for the odorant receptor for these carboxyl-methylated FAs [52]. These studies suggest that female attractiveness depends on a balance between repulsion and attraction, where the cocktail of CHC-related compounds tends to be repulsive, while the species-distinctive pheromone signature provides selective attractiveness for conspecific males. Given that pheromone biogenesis shares common enzymes, such as ACC, FATP, KAR/spidey [31], with the synthesis of the oocyte-activating VLCFA, a deficiency in the former pathway should also impair the latter. Therefore, it raises the question whether these parallel pathways are totally distinct or whether they have coevolved to improve the reproductive fitness.

Deciphering the physiological processes of sexual reproduction constitutes a major scientific challenge, relevant both to medical research and to control agricultural pest propagation. To our knowledge, our study provides the first evidence in the animal kingdom that egg activation can be controlled by a non-genital physiological signal. Notably, in aquatic species with external fertilization, ionic changes in either Na^+^ or Mg^2+^ in the environmental media are also required, underscoring the notion that spermatozoon entry is not the sole egg activation trigger. In conclusion, the search of physiological non-genital inputs for egg activation in other metazoan species warrants further investigation.

## Material and methods

### Fly stocks

The *1407-gal4* [53], *BO-gal4*, *svp-gal80* [39], *promE-gal4* [29], *P[ProtB-DsRed-monomer*,*w+]* [54], *Cg-gal4*, *daughterless-gal4*, *Tub-gal80ts* (BDSC: https://bdsc.indiana.edu, #7071, #55850 and #7019 respectively) lines were employed. For driver specificity and usage see S1C Table. The *promE-gal4* driver was used in combination with the *Tub-gal80ts* transgene; a temperature shift at 27°C happened at early metamorphosis for most experiments reported here, with the exception of S5C Fig, where it happened at L2/L3 transition. Inducible *UAS-RNAi* lines (S1A and S1B Table) were obtained from VDRC [55], BDSC, NIG (https://shigen.nig.ac.jp/fly/nigfly) or previously described [32,56].

### Fertility screening

*1407-gal4>UAS-RNAi* females were mated with wild-type Canton-S males 4–6 days after adult eclosion. The subsequent day, males were removed and single females (20 for each genotype) were let to lay eggs in new vials, with vials being changed every second day.

### Mating and sperm analyses

Mating choice were performed as previously described [31]; two 4-day old virgin *promE-gal4* females, one control and one driving a *UAS-RNAi*, were tested with one wild-type male; the female chosen for copulation was noted after 15 min. For sperm counting and speed, females were mated with *P[ProtB-DsRed*,*w+]* males to label sperm nuclei [54]. For counting, spermathecae and seminal receptacle were dissected in PBS-1X, fixed 20 min in PFA-4% (Fisher scientific; 50-980-487), mounted in DABCO (Sigma-Aldrich) and spermatozoa were counted using a Zeiss Imager M2 fluorescent microscope. For sperm speed, seminal receptacles were carefully dissected to prevent tissue damage and mounted in Biggers, Whitten and Whittingham modified medium (95mM NaCl, 4.8mM KCl, 1.3mM CaCl2, 1.2mM MgSO4, 1.2mM KH2PO4, 5.6mM glucose, 25mM NaHCO3, 20mM HEPES, 0.6% fatty acid free BSA, pH7.6), supplemented with 0.5mM trehalose. Time lapse imaging was performed using a Zeiss Imager M2 fluorescent microscope (10-seconds; 0.15-second frame interval); speed means were calculated from at least 19 spermatozoa per genotype (five females each).

### Activation analyses

For bleach resistance (Fig 5E), eggs were collected for 2hrs, approximately 30 eggs were incubated with commercial bleach (3,7% chlorax) for 10 min, rinsed with water, and the number of eggs still visible was counted. *In vitro* activation of stage-14 oocytes with hypotonic buffer was performed as previously described [40,48]. Briefly, stage-14 oocytes were dissected in Isolation Buffer (IB) (55 mM NaOAc, 40 mM KOAc, 110 mM sucrose, 1.2 mM MgCl2, 1 mM CaCl2, 100 mM Hepes, adjusted to pH 7.4 with NaOH). Oocytes were maintained in fresh IB until their activation by 5 successive 5-min baths in Activating Buffer (AB) (3.3 mM NaH2PO4, 16.6 mM KH2PO4, 10 mM NaCl, 50 mM KCl, 5% PEG8000, 2 mM CaCl2, adjusted to pH adjusted to 6.4 with 1:5 NaOH:KOH). To monitor development (S7 Fig), eggs were maintained in modified Zalokar’s buffer (ZAB) (9 mM MgCl 2, 10 mM MgSO4, 2.9 mM, NaH2 PO4, 0.22 mM NaOAc, 5 mM glucose, 27 mM glutamic acid, 33 mM glycine, 2 mM malic acid, 7 mM CaCl2, adjusted to pH adjusted to 6.8 with 1:1 NaOH:KOH). After 15 min of recovery in ZAB buffer, embryos were transferred into fresh vials with a thin layer of the same buffer. For dissected oocytes (Fig 6C), we observed that after 2,5 min in 2% chlorax solution, 50% of control oocytes were lysed; in these conditions, we assessed the percentage of lysed oocytes from *FASN3-* and *CG6432-*oeKD females.

### Imaging

Dissected oenocytes and ovaries were fixed and labeled with DAPI (1μg/mL; Sigma-Aldrich) and Nile-Red (400 nM; Sigma-Aldrich) as previously described [31,57]. For immunostaining of sperm flagella and meiotic spindles, eggs were dechorionated for 2 min in commercial bleach (3,7%chlorax), fixed in a 1:1 heptane:methanol mixture and stored at -20°C. Notably, dechorionation efficiency varies significantly depending on the bleach batch, particularly for *FASN3-* and *CG6432-*oeKD eggs that are highly sensitive to bleach treatment. Next, they were gradually rehydrated in 25, 50, 75% series in PBS 0.1%Triton-X100 and methanol for 10 min. Then, they were washed three times for 10 min each with PBS 0.1%Triton-X100 and incubated overnight at 4°C with anti-acetylated-tubulin at 1/500 (Sigma-Aldrich; T6793) or α-tubulin at 1/200 (Sigma-Aldrich; T5168) and DAPI (1μg/mL; Sigma-Aldrich); next, eggs were washed three times and incubated with an anti-mouse antibody Alexa-Fluor-568 or 488 nm at 1/500 (Invitrogen; A11061 and A11001). Samples were mounted in DABCO (Sigma-Aldrich) and analyzed on a Leica SP8 confocal-laser-scanning microscope. For histology, female genital tracts were dissected, fixed in glutaraldehyde and post-fixed in osmium tetroxide, dehydrated in ethanol followed by propylene oxide. Samples were embedded in Epon and sectioned into 1 μm thick slices. Sections were stained in Toluidine Blue and scanned with a Grundium Ocus 40.

### Abdomen embedding and sectioning

Whole *Drosophila* abdomens, excluding heads and thoraces, were fixed 4h at 4°C in 0.1 M phosphate buffer (PB) pH 7.2, 4% paraformaldehyde and 0.5% glutaraldehyde. Next, the abdomens were washed in PB and incubated 30 min in 100 mM glycine to quench aldehyde, followed by overnight incubation at 4°C under stirring in PB buffer plus 1mg/ml DAPI. Samples were progressively dehydrated in graded ethanol series (50, 70, 90, 90%), with the 90% ethanol solution supplemented with 1mM of dithiothreitol (DTT). Embedding in graded series (50, 60, 75, 100, 100%) of resin (LR White Resin, Agar Scientific, Oxford instruments) mixed with ethanol was processed manually for 2 days at -20°C. Samples were polymerized in gelatin capsules (TAAB) at 50°C for 24 h. Sections (2μm) were cut with an ultramicrotome EM UC6 (Leica Microsystems) with JUMBO diamond (DIATOME), collected on glass coverslips before mounting in DABCO (Sigma-Aldrich). Images were acquired with a Zeiss Imager M2 fluorescent microscope.

### Biochemistry

Experiments performed as previously described: CHC measurement [31]; TAG measurement [34]; protein extracts and western-blotting [58]. The Smaug antibody, kindly provided by M Simonelig, was used at 1/1000 for western blotting [59]. Quantification of western-blots was performed using ImageJ.

### Statistics

Statistical analysis were performed with PRISM/Graphpad. Significance levels are indicated as *, ** and *** corresponding to P< 0.05, 0.01 and 0.001, respectively. T-test were utilized for Figs 1B, 1C, 1D, 2I, 3D, 3E, 4A, 4B, 5E, 5F, 6B, 6C and for S4, S5, S6 and S7. Chi-2 tests were utilized for Figs 3C, 5D and 6A. ANOVA was applied for S2A and S3 Tables.

## Supporting information

S1 Raw Data Raw data for Figs 1B, S1, S4, S5, S6 and S7 and S2 and S3 Tables. (ZIP)

S1 Fig Screening for sterility. (**A**) *1407-gal4>UAS-RNAi* females crossed to Canton-S males were let to lay eggs during six days (D) in three successive vials and the progeny was counted at adult emergence. The y-axis indicates total numbers (cumulative) of emerging flies after 2, 4 and 6 days. For each genotype, values are mean of emerging flies from 20 females maintained in separate tubes. (**B**) Reciprocal crosses to test male fertility.
(PDF)

S2 Fig Oenocyte viability during adult lifespan. **(A-R)** Dorsal abdominal cuticles stained for lipids and nuclei of *1407>UAS-GFP* females either control (A-C), or directing *UAS-RNAis* to *KAR/spidey* (D-F), *ACC* (G-I), *FATP* (J-L), *FASN3* (M-O) or *CG6432* (P-R). Females were dissected 9 days (A,D,G,J,M,P), 18 days (B,E,H,K,N,Q) or 27 days (C,F,O,R) after adult emergence, except ACC- and FATP-RNAis flies that did not survive longer than 24 days (I,L). Oenocytes were visualized by GFP (green) the nuclei by DAPI (silver) and the fat body by Nile red. Since the Nile red partially interferes with the GFP channel and that the GFP intensity varied a lot for unknown reasons, the lipid staining appeared either red (strong GFP) or orange (low GFP). Note that oenocyte loss appeared earlier in age for *FATP-RNAi* (K,L) than for *KAR/spidey-RNAi* (F). Scale bars: 100μm.
(TIF)

S3 Fig CG6432 homologues. Peptidic sequence alignment of CG6432 (Dm) to the best homologues Acss3 in mouse (Mm) and Acs1 in the yeast *Saccharomyces cerevisiae* (Sc), using www.uniprot.org.
(PDF)

S4 Fig CHCs in CG6432 knockdown males. Means values of CHCs from 10 control (white) or 10 *1407-gal4>CG6432-RNAi* (black) males. Note the drop of mbCHCs that is compensated by an increase in linear CHCs in *CG6432* knockdown males.
(PDF)

S5 Fig **Fertility tests: (A)** Testing additional lines directing either double strand RNA (Ri) or short-hairpin RNA (sh): pupal progeny of *promE-gal4* females either control (black) or expressing *CG6432-shRNA* (green), *elo*^*CG6660*^*-RNAi* (white) *elo*^*CG6660*^*-shRNA* (dashed), *FASN3-shRNA* (purple), *KAR/spidey-shRNA* (orange) or *FATP-RNAi* (dark grey); developing animals were switched to 27°C at early metamorphosis, 3 adult females were mated to wild type males 3–5 days after adult eclosion; males were removed the day after and females were transferred to new vials every day. (**B**) Pupal progeny of *1407-gal4* females either control (black) or expressing *FASN3-RNAi* (purple) or *CG6432-RNAi* (green); 3-day old females were mated to wild type males; males were removed the day after and females were transferred to new vials every day for offspring counting. (**C**) Pupal progeny of *promE-gal4* females either control (black) or expressing *FASN3-RNAi* (purple), *CG6432-RNAi* (green), *CG3415-RNAi* (light grey) or *CPT1-RNAi* (dark grey); developing animals were switched to 27°C at L2/L3 transition, adult females were mated to wild type males 4 days after adult eclosion and transferred to new vials every day; day 1 (1d) corresponds to the eggs laid by 5-day old females. (**D**) Adult progeny of *1407-gal4* females expressing either *FASN3-RNAi* (purple) or *CG6432-RNAi* (green), with (dotted colors) or without (plain colors) the *svp-gal80* transgene; 3-4-day old females were mated to wild type males; males were removed the day after and females were transferred to new vials every days for offspring counting. Bars correspond to the mean values of pupae (5 replicates with 3 females each in A and C) or adults (20 females individually tested in B and D) obtained from each egg collection.
(PDF)

S6 Fig Germline fertility test. *nanos-gal4* 6-days old females either control (black) or expressing *FASN3-sh* (purple), *CG6432-sh* (green) were transferred every second or third days in new vials. Total pupal progeny is shown for each collection but calculated for one day. Females were maintained at 27°C since L3 early pupal stages and mated with wild type males during 6 days. Next, three females were transferred in new tubes as indicated. Mean values are obtained from six replicates (3 females each) for control and seven replicates for *FASN3-sh* and *CG6432-sh*.
(PDF)

S7 Fig In vitro activation test of dechorionated eggs. Pupal progeny from dechorionated eggs laid by *promE-gal4* 10-day old females either control (Co) or expressing *FASN3-RNAi* or *CG6432-RNAi*, with (A) or without (No A) *in vitro* activation.
(PDF)

S1 Table List of *UAS-RNAi*, *UAS-shRNA* and driver lines. (**A**) List of the genes and the corresponding lines screened for female sterility (fertility column) using the *1407-gal4* driver. (**B**) List of the genes and the corresponding lines tested for female sterility using the *prom-gal4* driver. RNAi and shRNA lines were provided by NIG, BDSC or VDRC; three of them have been generated in S Eaton, CWT or JM laboratories (Stock column). The lines from BDSC express shRNA. (**C**) List of the gal4 drivers (left column), their tissue specific expression (middle column), and their usage (right column) in the present study.
(PDF)

S2 Table **CHC amounts in *1407>CG6432-RNAi* (*6432i*) flies:** 4-5-day old males (top) or females (bottom). First column: CHC identities; elemental composition is indicated as the carbon chain length followed by the number of double bonds; Me- are mbCHCs. CHCs are expressed in ng/fly (Tot) or in percentages relative to total CHC amount as the mean (± SEM) of CHCs produced by 10 flies maintained 4 days at 25°C.
(PDF)

S3 Table **CHC amounts in *1407-gal4> elo***^***CG6660***^***-RNAi* (*6660i*) flies:** 4-5-day old males (top) or females (bottom). First column: CHC identities; elemental composition is indicated as the carbon chain length followed by the number of double bonds; Abbreviation as in S2 Table.
(PDF)

S1 Movie Displacement of spermatozoa in the seminal receptacle of a *promE-gal4* control female. (AVI)

S2 Movie Displacement of spermatozoa in the seminal receptacle of a *promE-gal4*>*FASN3-RNAi* female. (AVI)

S3 Movie Displacement of spermatozoa in the seminal receptacle of a *promE-gal4*>*CG6432-RNAi* female. (AVI)
