# Supplementary material for: A fatty acid anabolic pathway in specialized-cells sustains a remote signal that controls egg activation in Drosophila
Source: PLoS Genet. 2024 Mar 14;20(3):e1011186. doi: 10.1371/journal.pgen.1011186 (PMC10965083; doi:10.1371/journal.pgen.1011186)
Supplement: S1 Raw Data — (ZIP) [file pgen.1011186.s001.zip › Poidevin DATA/Fig2/F_Summary_GFP_POI8.pptx]

## Slide 1
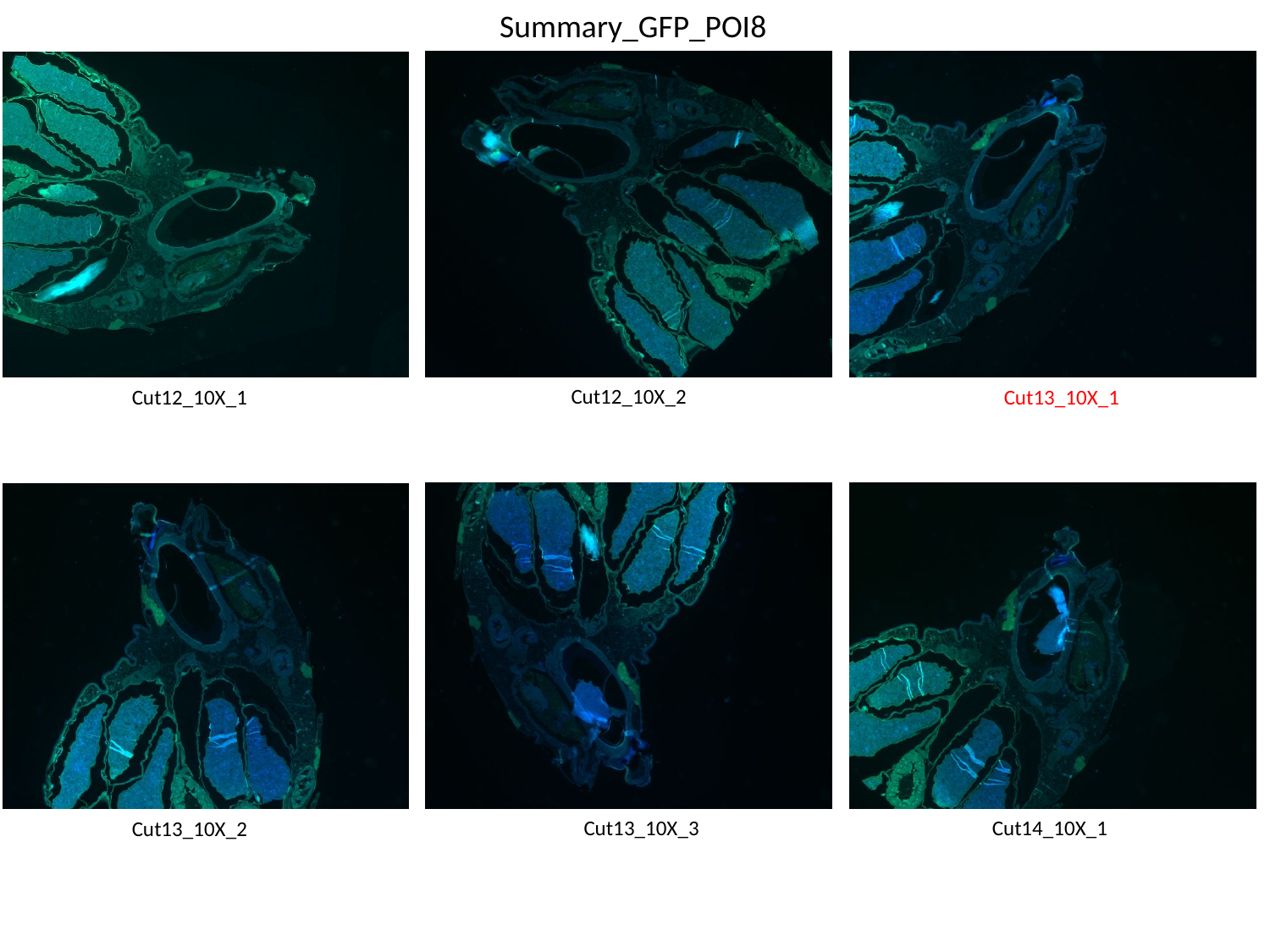

Summary_GFP_POI8
Cut12_10X_2
Cut12_10X_1
Cut13_10X_1
Cut14_10X_1
Cut13_10X_3
Cut13_10X_2

## Slide 2
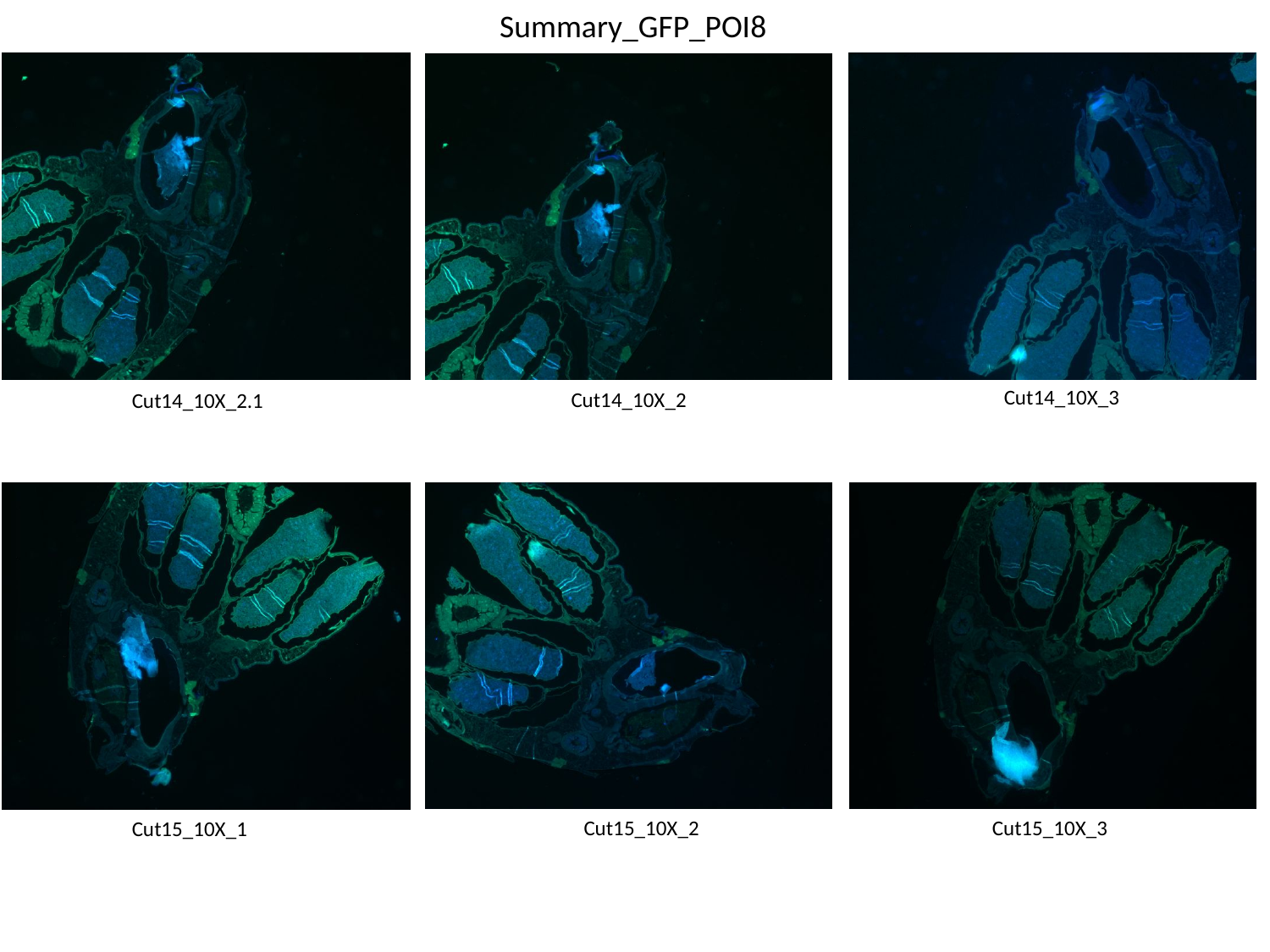

Summary_GFP_POI8
Cut14_10X_3
Cut14_10X_2
Cut14_10X_2.1
Cut15_10X_3
Cut15_10X_2
Cut15_10X_1

## Slide 3
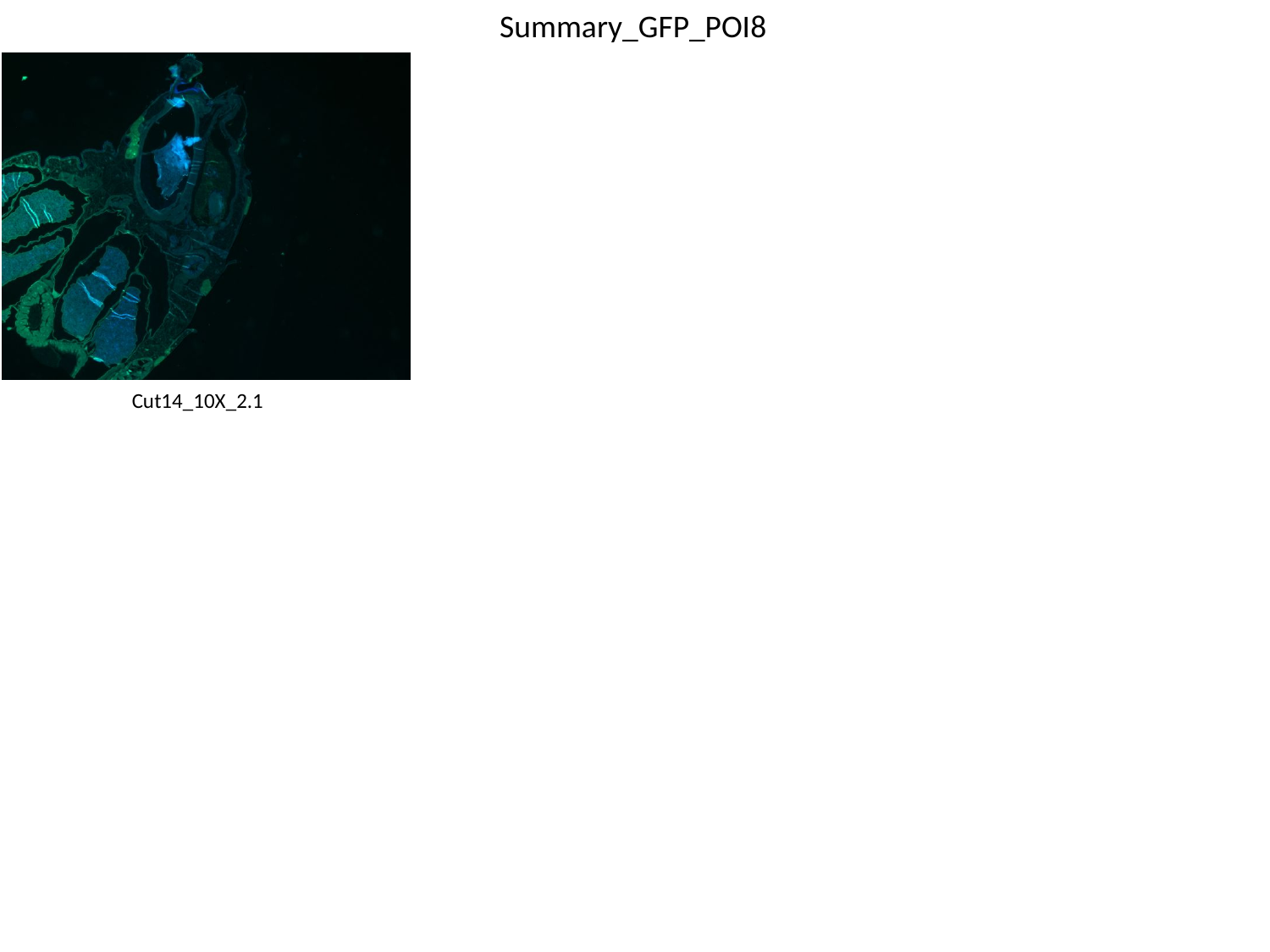

Summary_GFP_POI8
Cut14_10X_2.1
