# Supplementary figures and images for: A fatty acid anabolic pathway in specialized-cells sustains a remote signal that controls egg activation in Drosophila
Source: PLoS Genet. 2024 Mar 14;20(3):e1011186. doi: 10.1371/journal.pgen.1011186 (PMC10965083; doi:10.1371/journal.pgen.1011186)

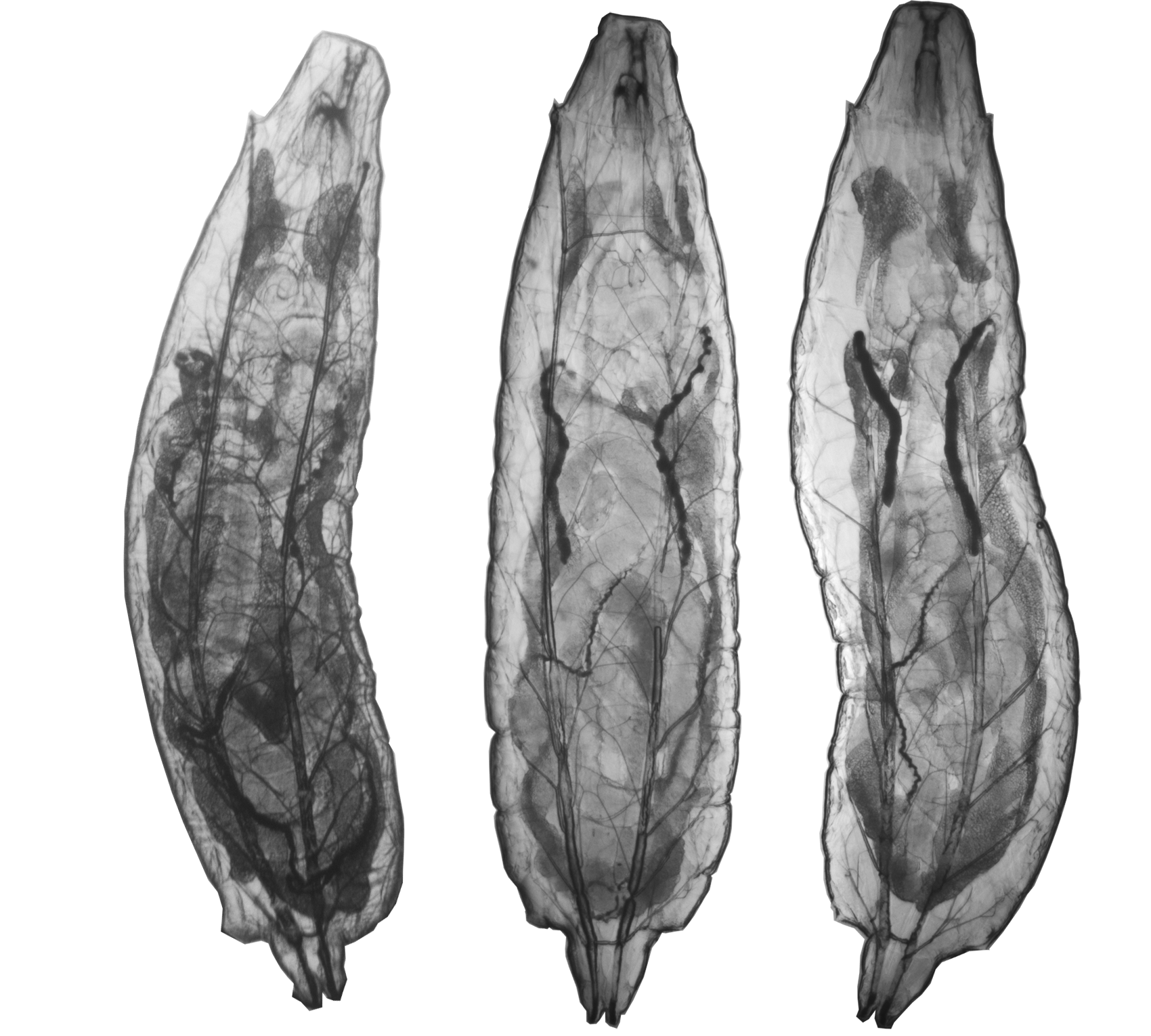

Supplement: S1 Raw Data — (ZIP) [file pgen.1011186.s001.zip › Poidevin DATA/Fig1/1A_Spiracles NEW.tif]

## Slide 1
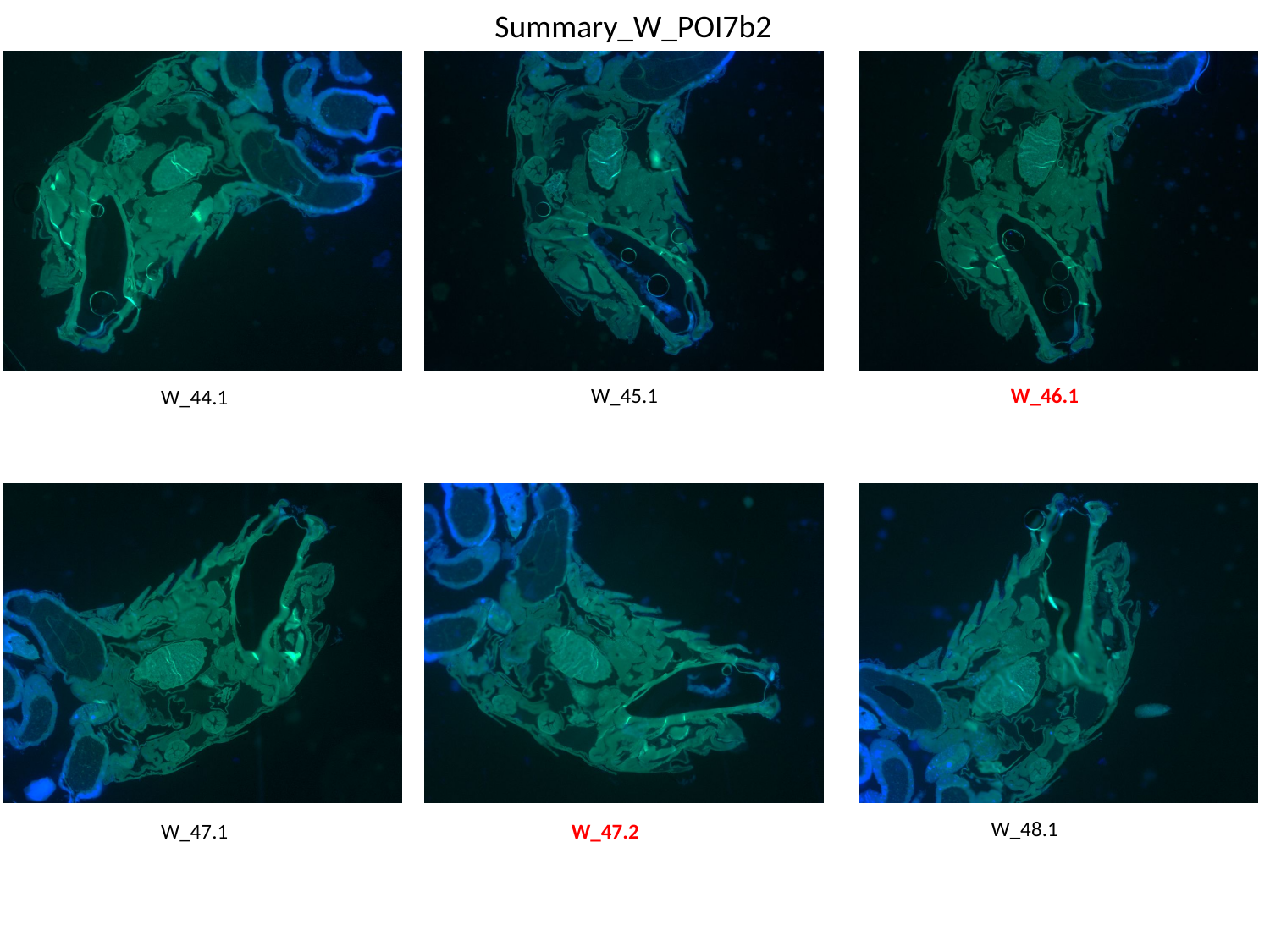

Summary_W_POI7b2
W_45.1
W_46.1
W_44.1
W_48.1
W_47.1
W_47.2

## Slide 2
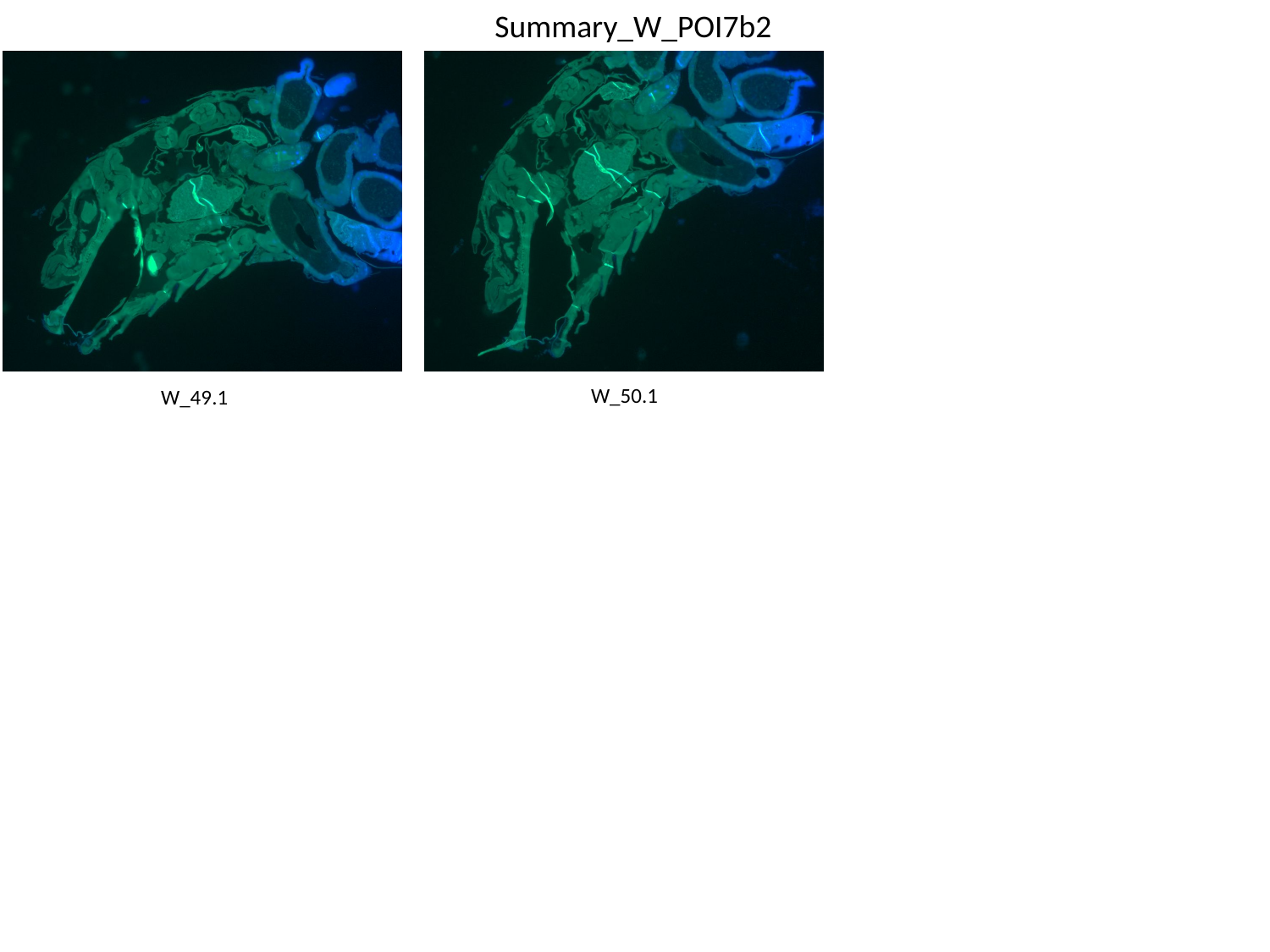

Summary_W_POI7b2
W_50.1
W_49.1

Supplement: S1 Raw Data — (ZIP) [file pgen.1011186.s001.zip › Poidevin DATA/Fig2/E_Summary_W_POI7b2.pptx]

## Slide 1
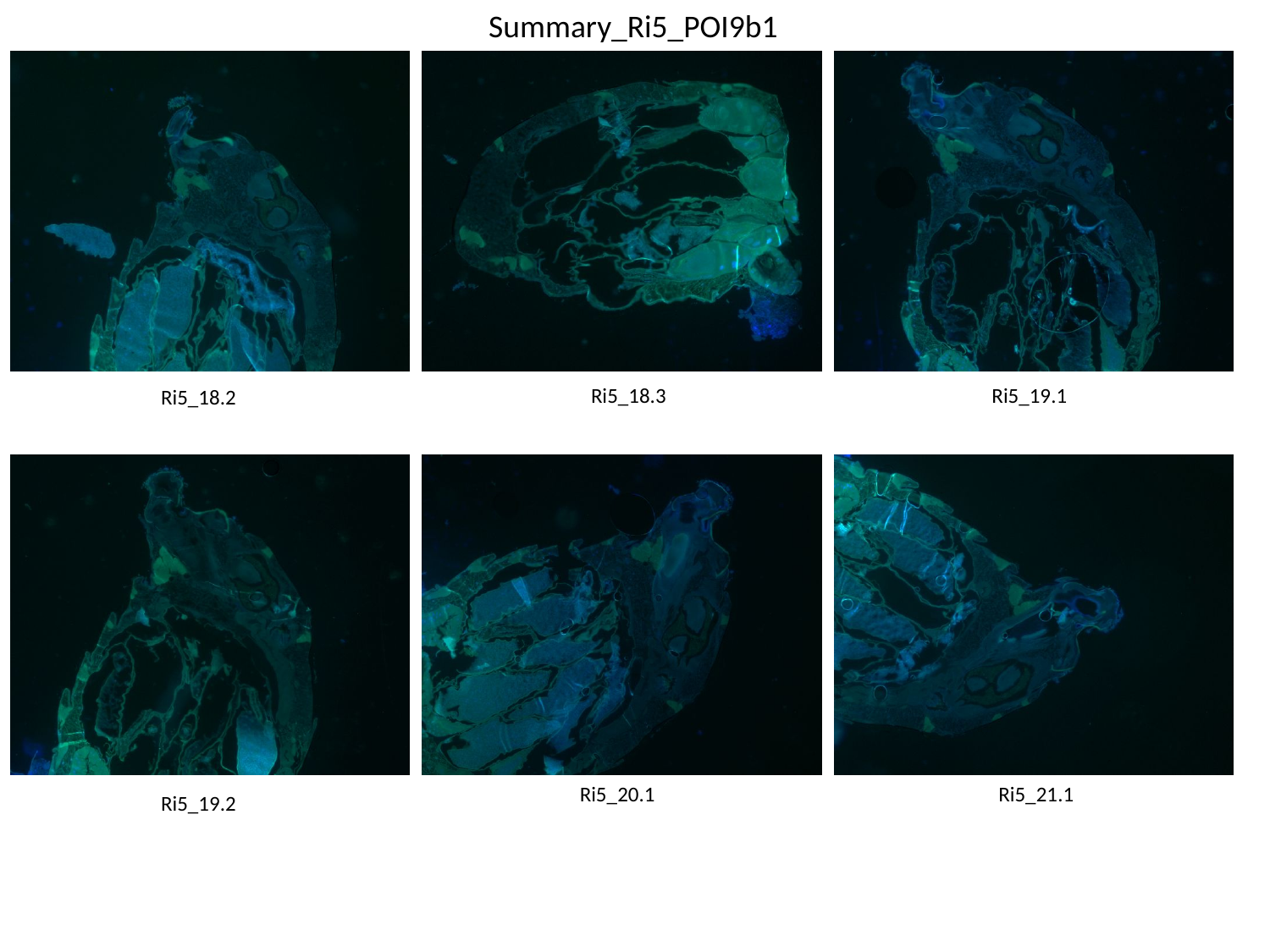

Summary_Ri5_POI9b1
Ri5_19.1
Ri5_18.3
Ri5_18.2
Ri5_21.1
Ri5_20.1
Ri5_19.2

## Slide 2
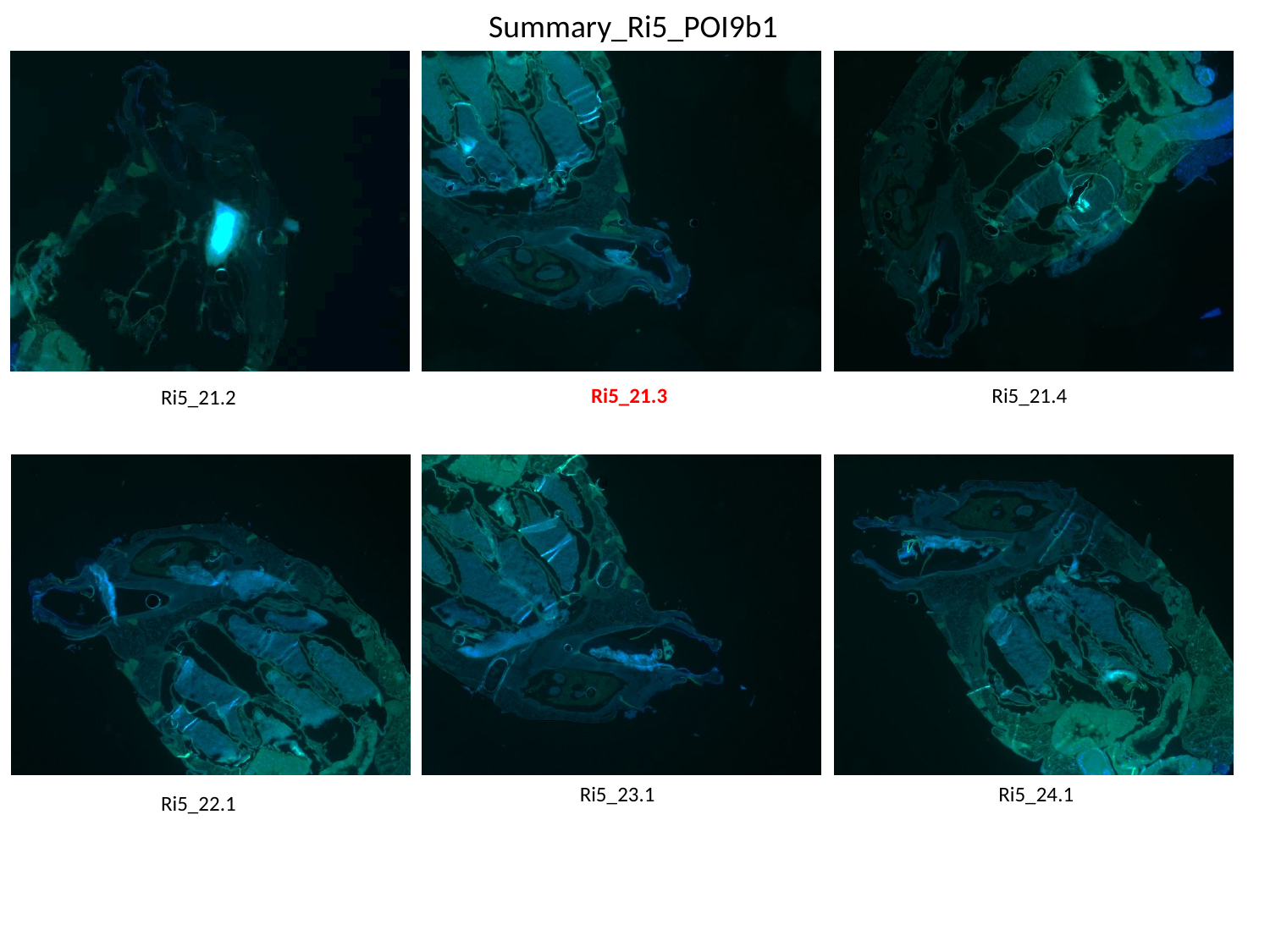

Summary_Ri5_POI9b1
Ri5_21.4
Ri5_21.3
Ri5_21.2
Ri5_24.1
Ri5_23.1
Ri5_22.1

Supplement: S1 Raw Data — (ZIP) [file pgen.1011186.s001.zip › Poidevin DATA/Fig2/G_Summary_Ri5_POI9b1.pptx]

## Slide 1
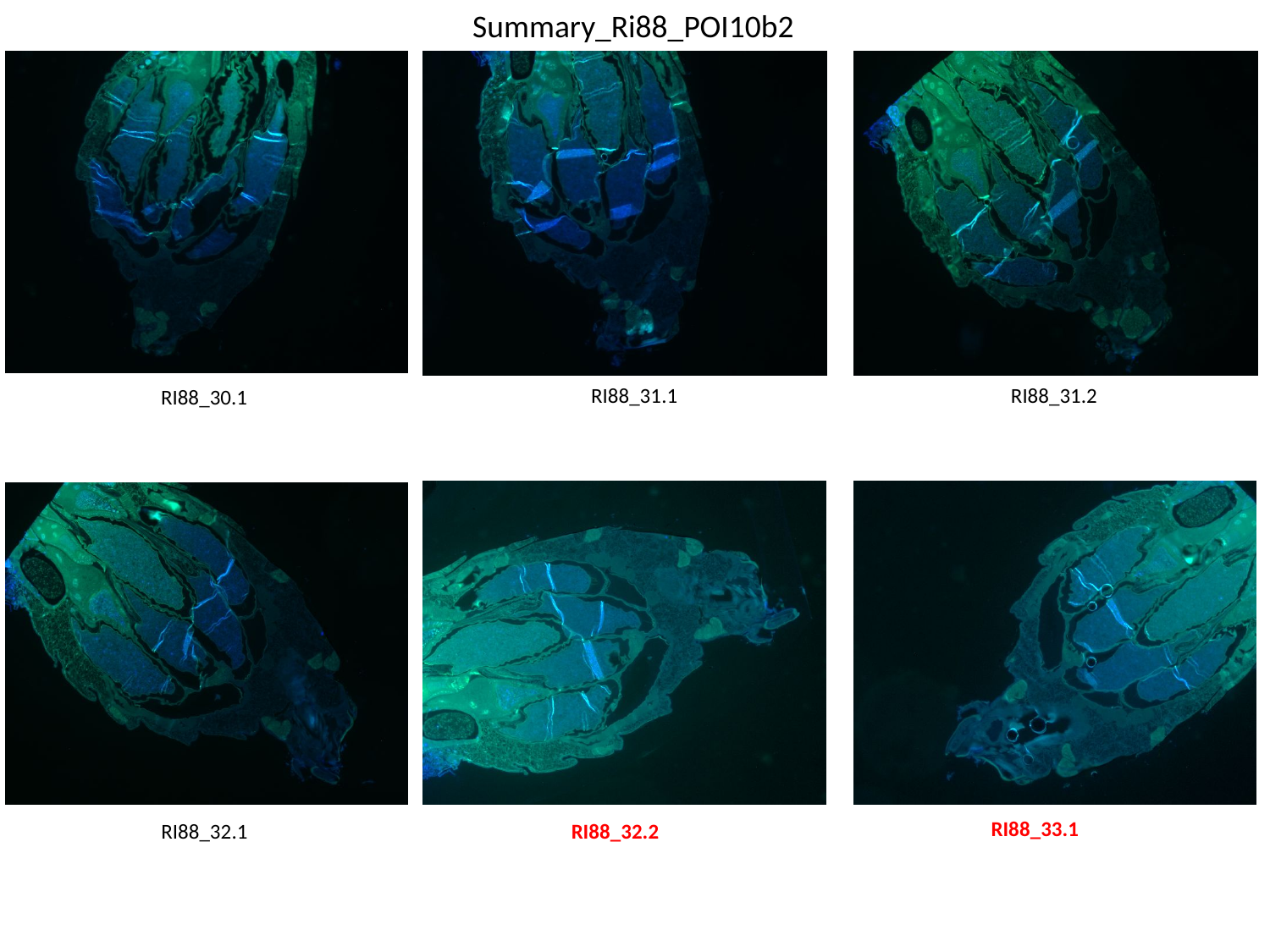

Summary_Ri88_POI10b2
RI88_31.1
RI88_31.2
RI88_30.1
RI88_33.1
RI88_32.1
RI88_32.2

Supplement: S1 Raw Data — (ZIP) [file pgen.1011186.s001.zip › Poidevin DATA/Fig2/H_Summary_Ri88_POI10b2.pptx]

## Slide 1
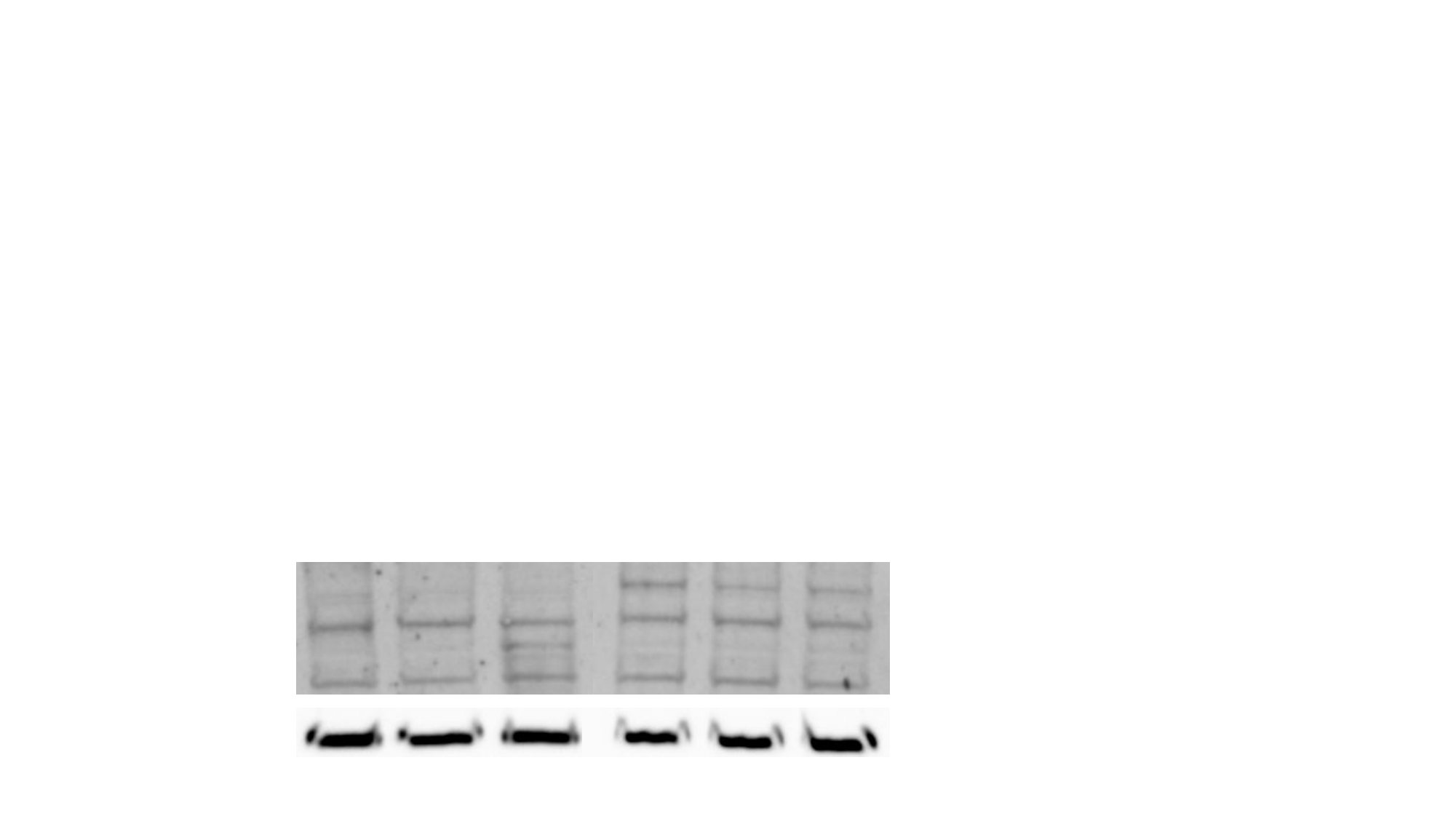

Supplement: S1 Raw Data — (ZIP) [file pgen.1011186.s001.zip › Poidevin DATA/Fig6/6B_ Western blot Smaug.pptx]

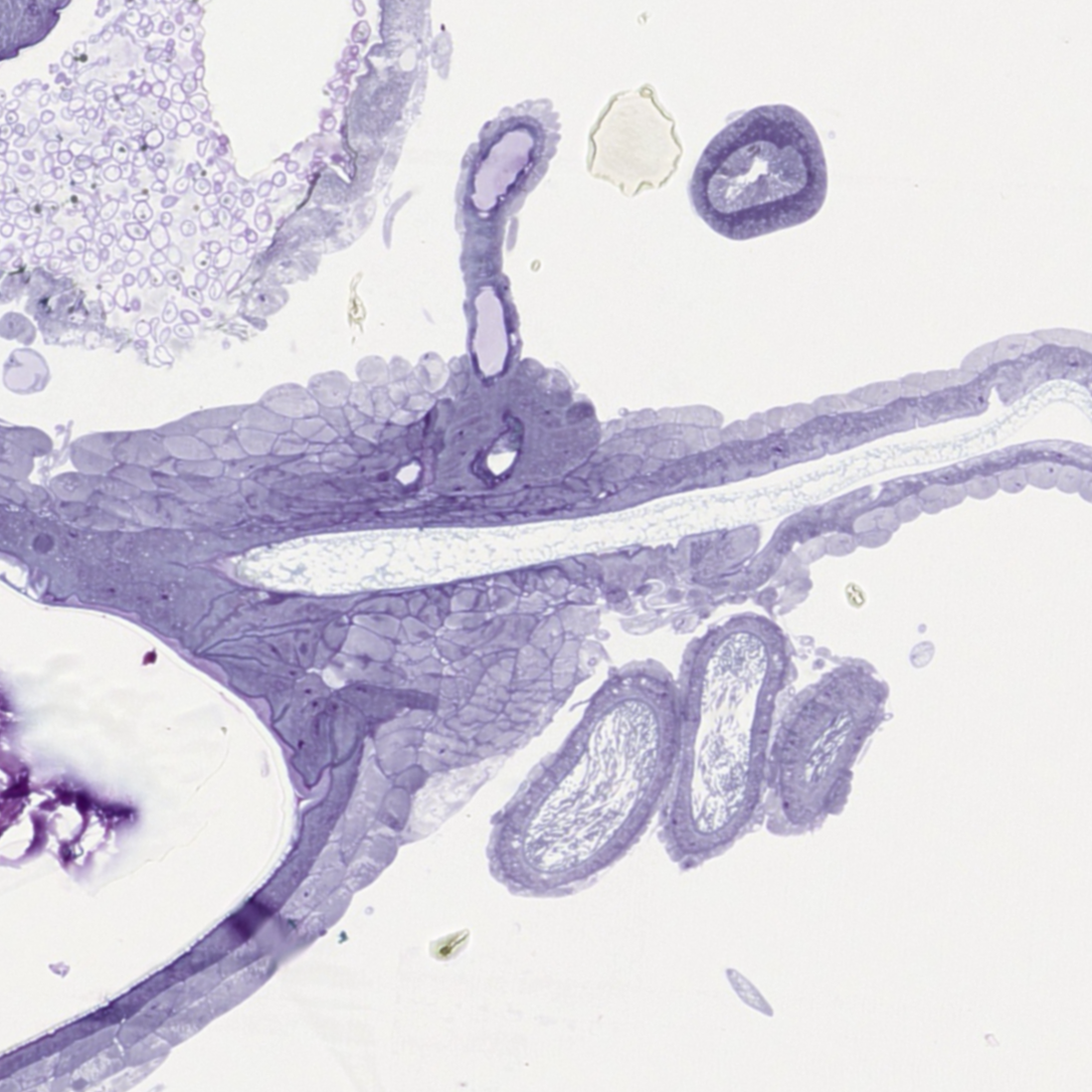

Supplement: S1 Raw Data — (ZIP) [file pgen.1011186.s001.zip › Poidevin DATA/Fig6/6D_Cytomine pictures/88-4_3-TIFF_CROP.tif (RGB).tif]

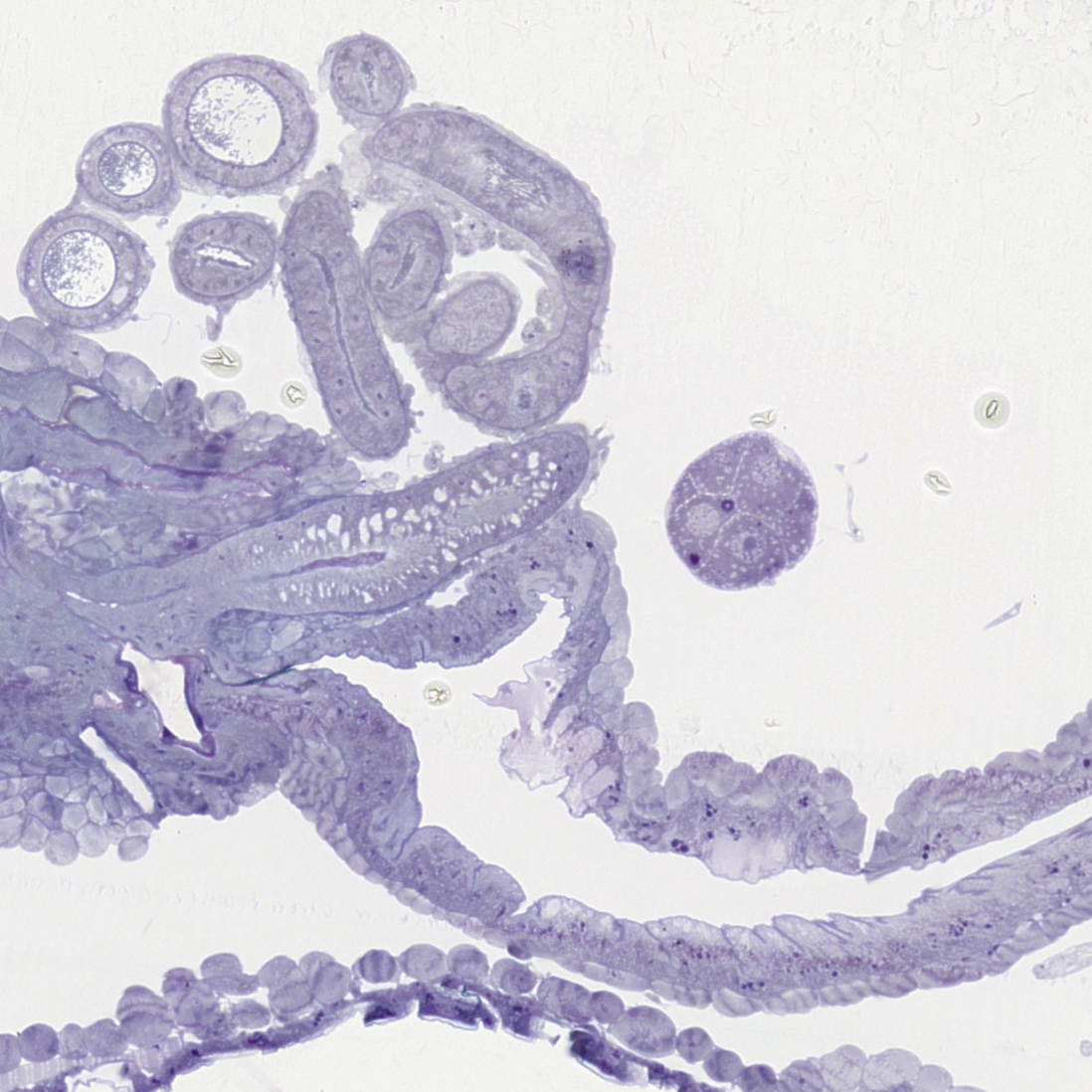

Supplement: S1 Raw Data — (ZIP) [file pgen.1011186.s001.zip › Poidevin DATA/Fig6/6D_Cytomine pictures/i5-4_3-TIFF_CROP.tif (RGB).tif]

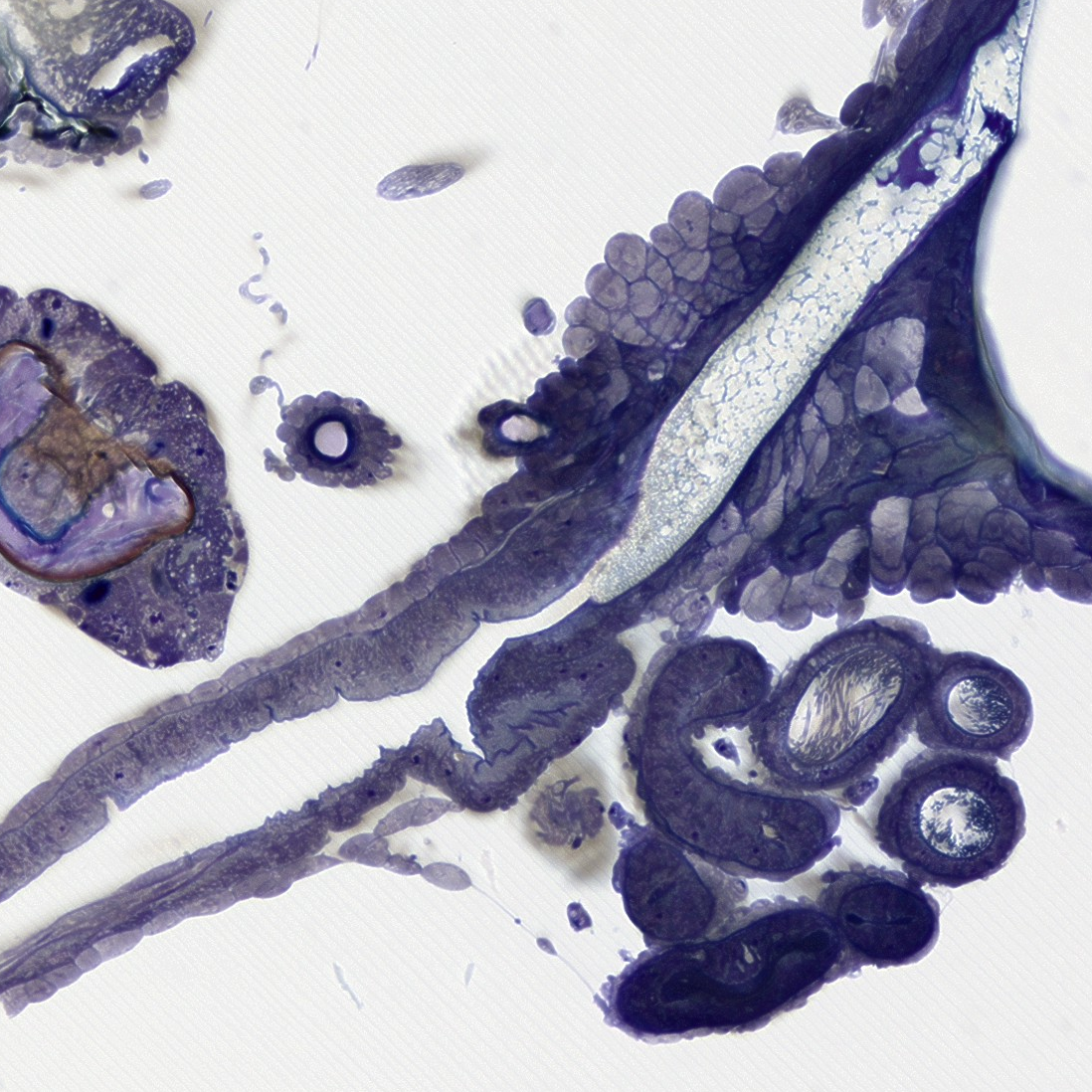

Supplement: S1 Raw Data — (ZIP) [file pgen.1011186.s001.zip › Poidevin DATA/Fig6/6D_Cytomine pictures/wt-4_3-TIFF_CROP.tif (RGB).tif]

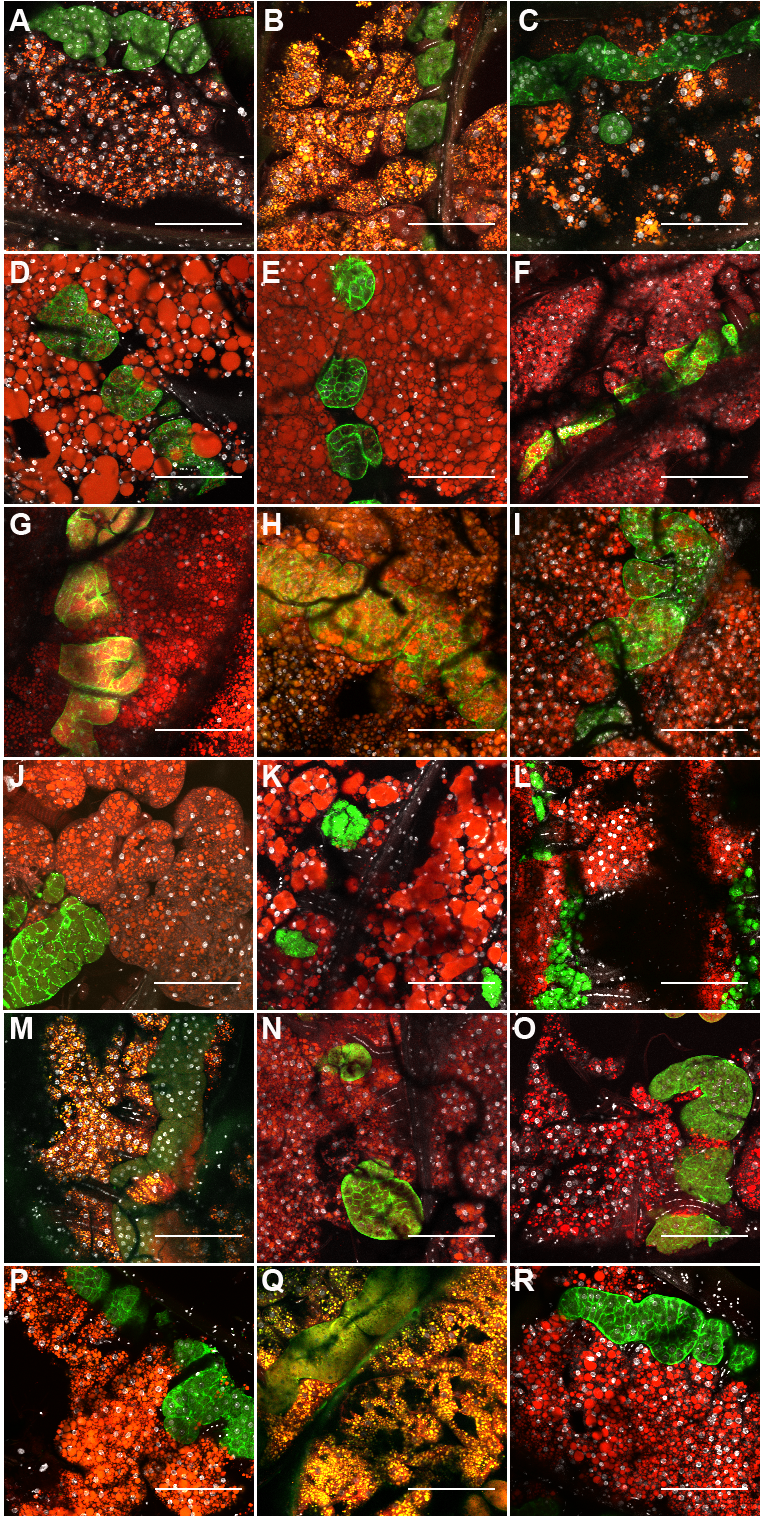

Supplement: S1 Raw Data — (ZIP) [file pgen.1011186.s001.zip › Poidevin DATA/SUP DATA/Supplementary figure S2/Supplementary Fig. 2.tif]

## Slide 1
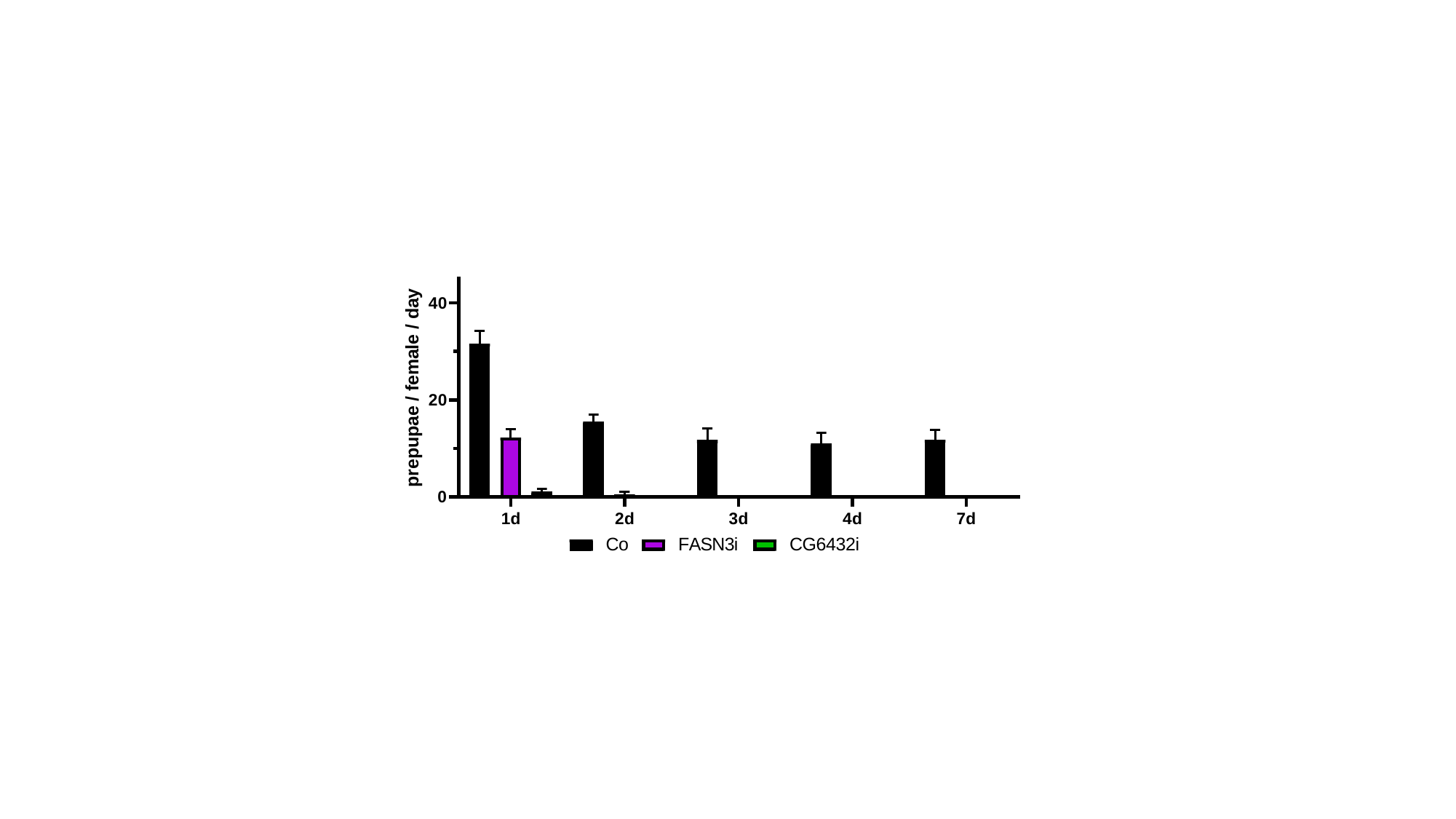

Supplement: S1 Raw Data — (ZIP) [file pgen.1011186.s001.zip › Poidevin DATA/SUP DATA/Supplementary figure S5/B_1407_FAS+6432-ponte.pptx]

## Slide 1
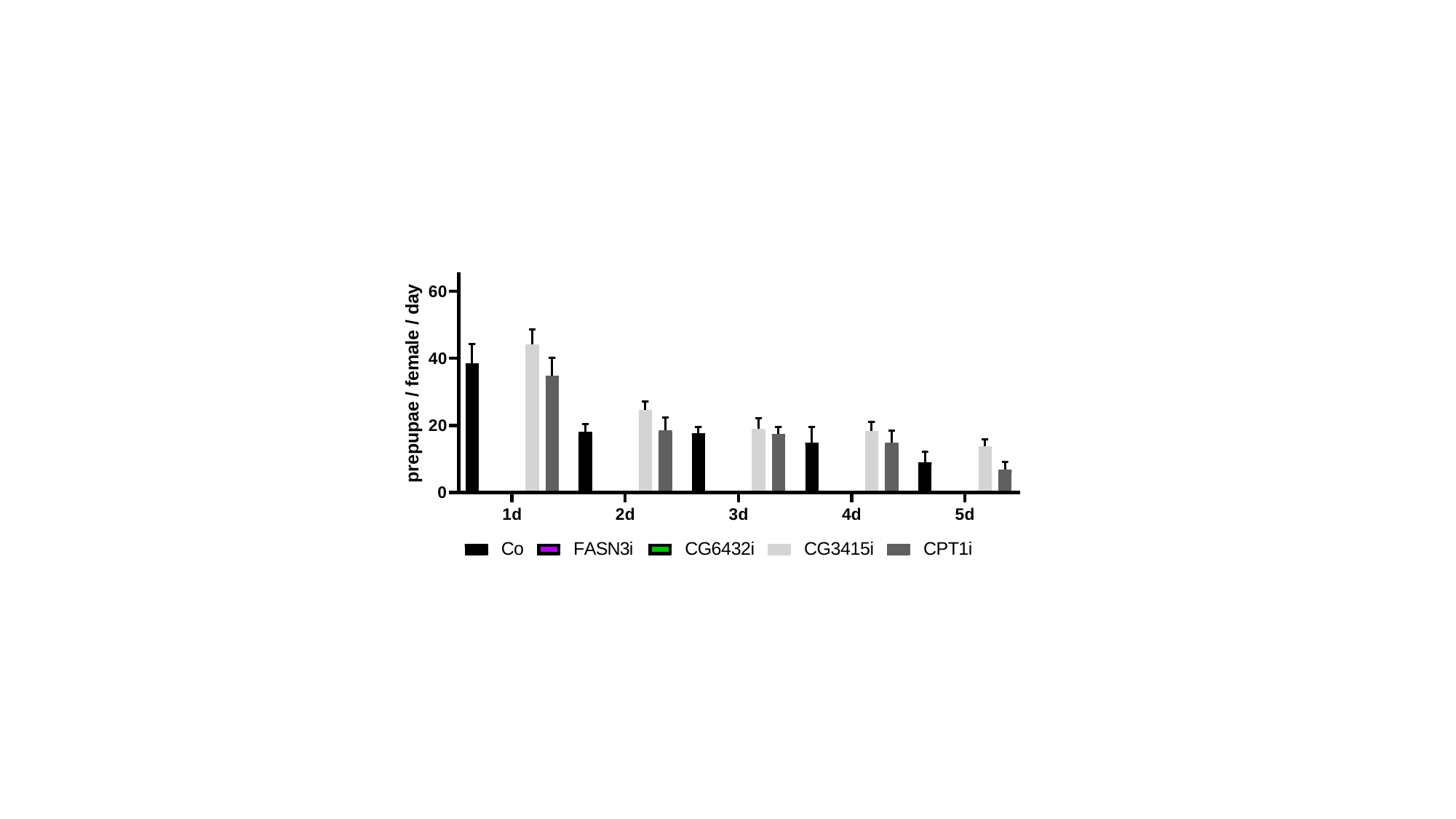

Supplement: S1 Raw Data — (ZIP) [file pgen.1011186.s001.zip › Poidevin DATA/SUP DATA/Supplementary figure S5/C_230707 Fertility SUP.pptx]

## Slide 1
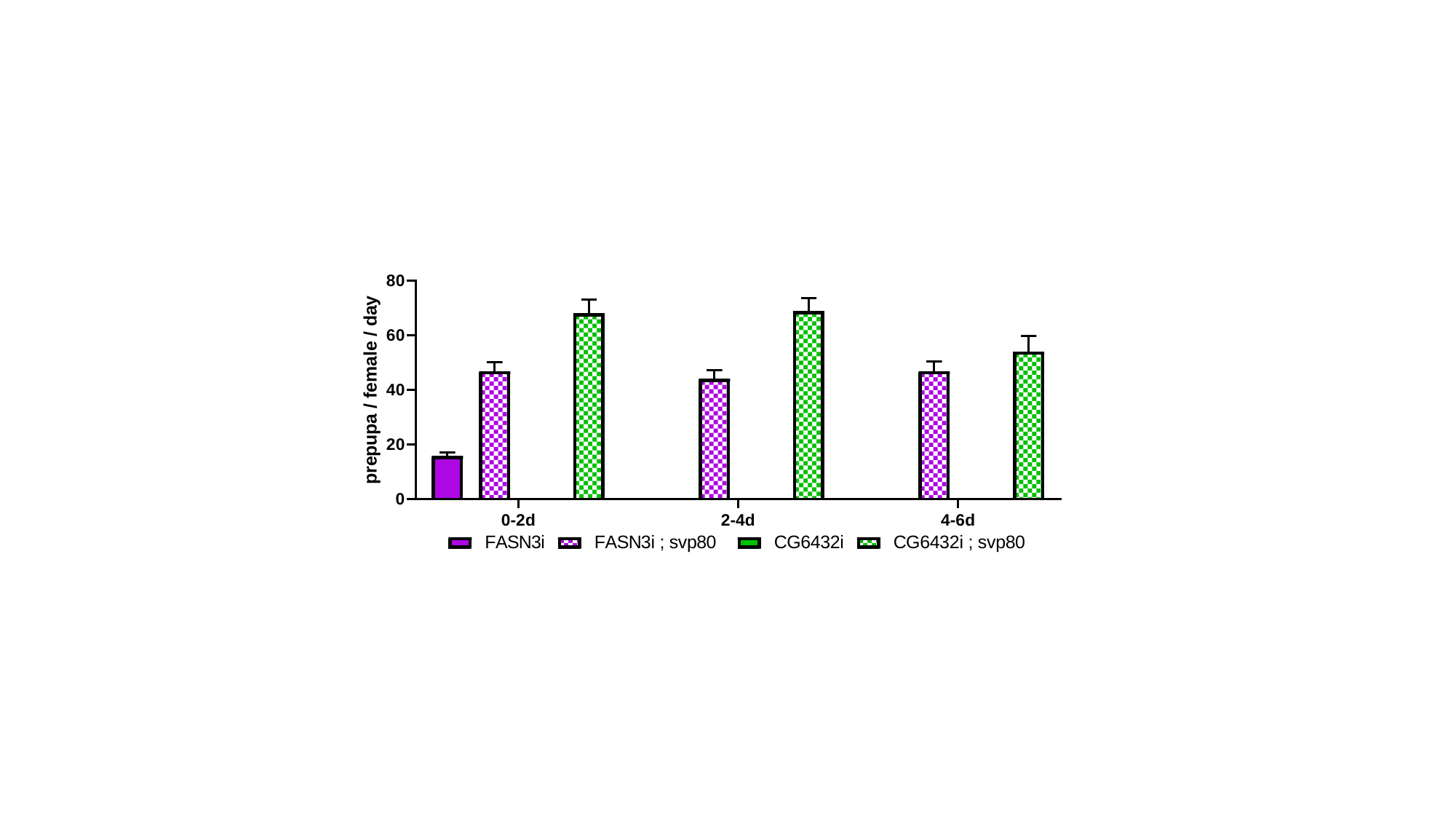

Supplement: S1 Raw Data — (ZIP) [file pgen.1011186.s001.zip › Poidevin DATA/SUP DATA/Supplementary figure S5/D_2016-10-PONTE fas3i-6432igraphes SVP SUP.pptx]

## Slide 1
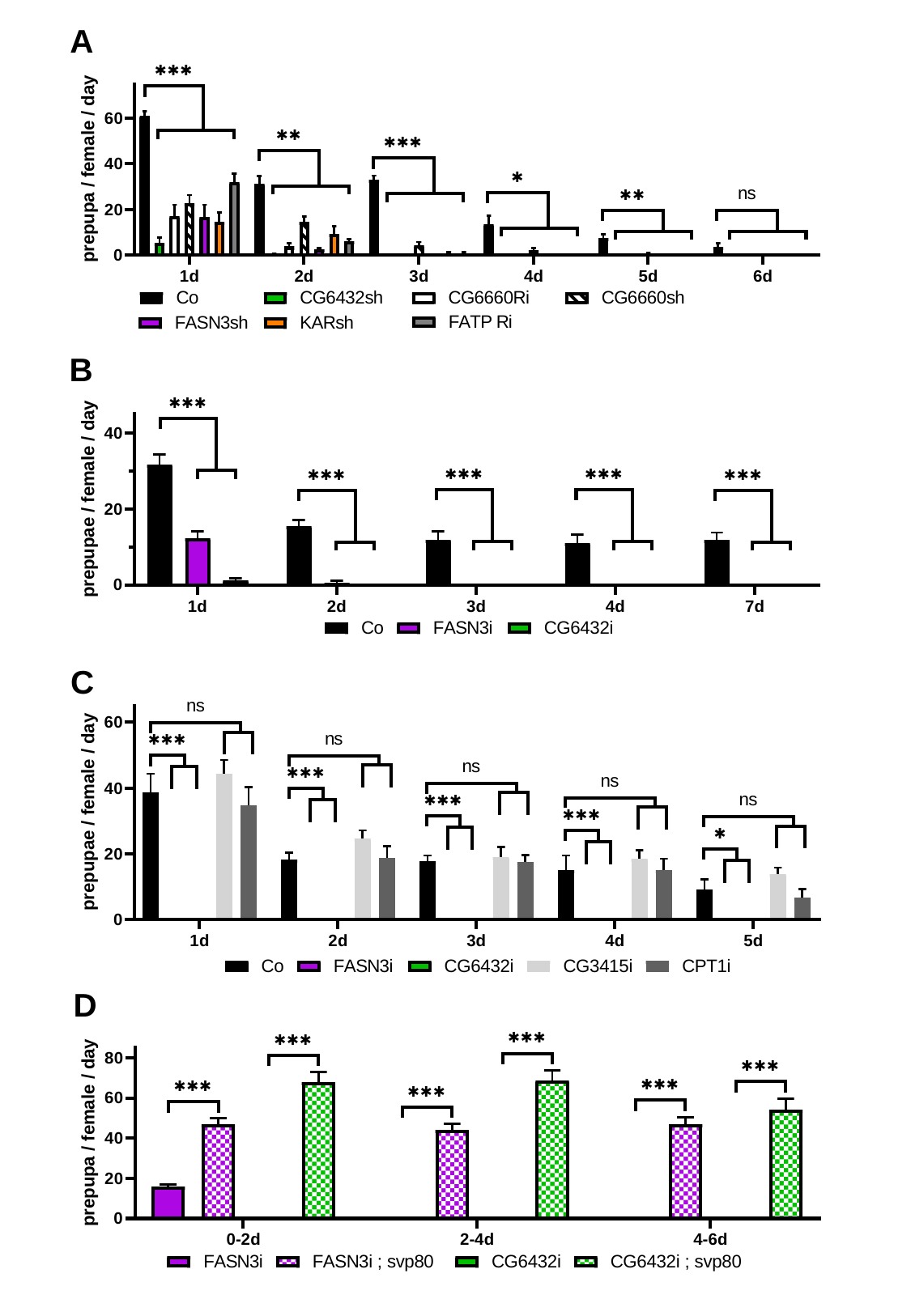

Supplement: S1 Raw Data — (ZIP) [file pgen.1011186.s001.zip › Poidevin DATA/SUP DATA/Supplementary figure S5/Supplementary Figure S5.pptx]

## Slide 1
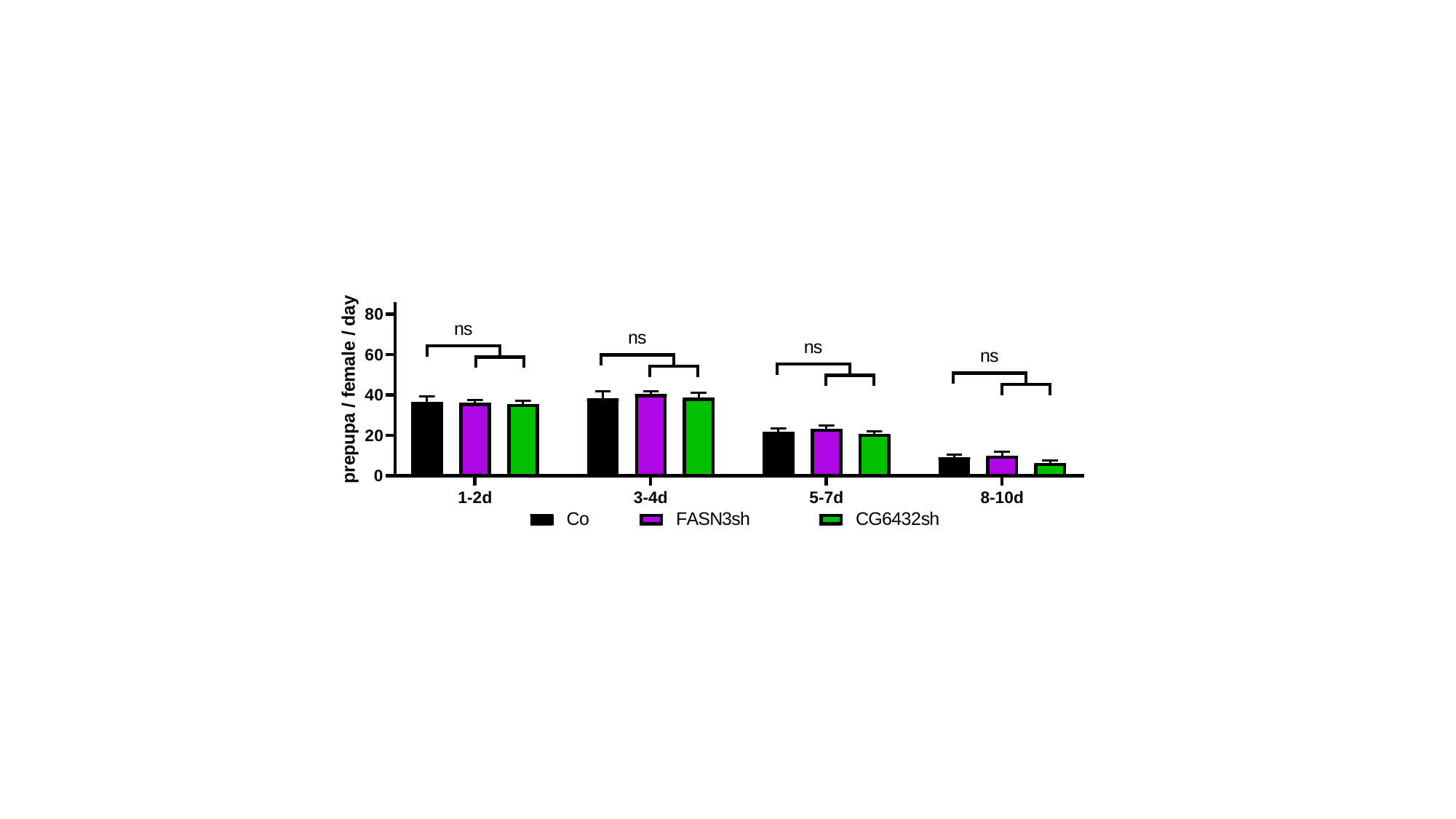

Supplement: S1 Raw Data — (ZIP) [file pgen.1011186.s001.zip › Poidevin DATA/SUP DATA/Supplementary figure S6/231117 Nanos_S6SUP.pptx]

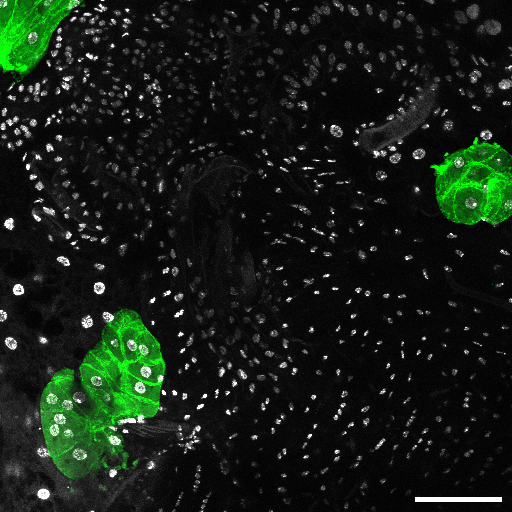

Supplement: S1 Raw Data — (ZIP) [file pgen.1011186.s001.zip › Poidevin DATA/Fig2/C_17_2021 10 19.lif_GFP_uterus_gainGFP 600_17-1_flatten.tif]

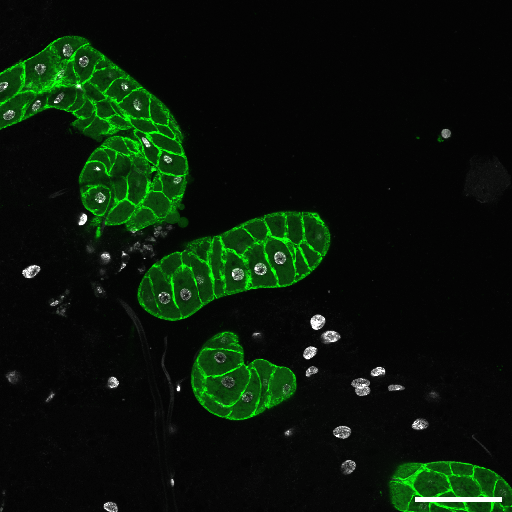

Supplement: S1 Raw Data — (ZIP) [file pgen.1011186.s001.zip › Poidevin DATA/Fig2/A_4_4_2021 10 19 PromEXGFP.lif_GFP_cuticule_1.tif]

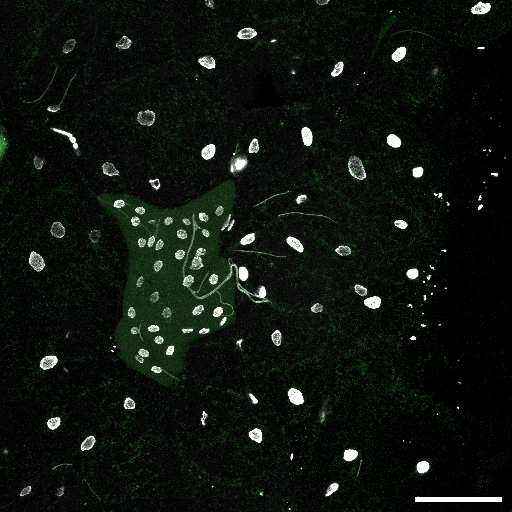

Supplement: S1 Raw Data — (ZIP) [file pgen.1011186.s001.zip › Poidevin DATA/Fig2/B_2_2_2021 10 19.lif_PromExGFP SVP80_cuticuke_1.tif]

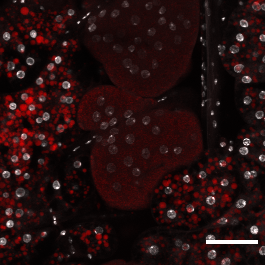

Supplement: S1 Raw Data — (ZIP) [file pgen.1011186.s001.zip › Poidevin DATA/Fig3/A1_AVG_20201112_3weeks.lif - WT 3-1_1.tif]

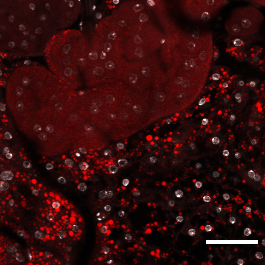

Supplement: S1 Raw Data — (ZIP) [file pgen.1011186.s001.zip › Poidevin DATA/Fig3/A2_AVG_20201112_3weeks.lif - RI45 1_1.tif]

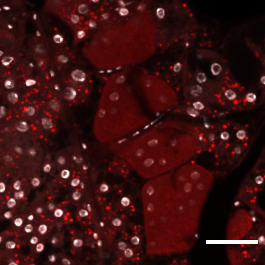

Supplement: S1 Raw Data — (ZIP) [file pgen.1011186.s001.zip › Poidevin DATA/Fig3/A3_AVG_20201112_3weeks.lif - RI88 3-1_1.tif]

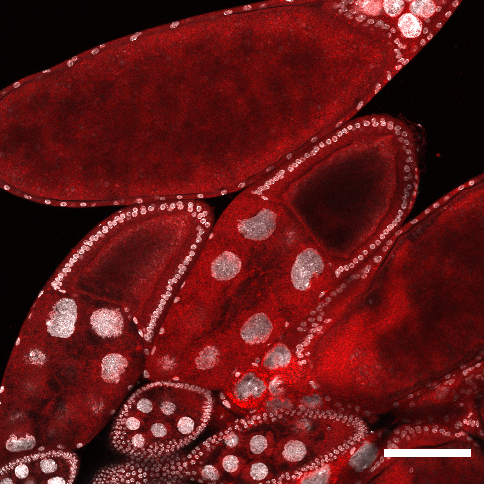

Supplement: S1 Raw Data — (ZIP) [file pgen.1011186.s001.zip › Poidevin DATA/Fig3/B1_MAX_20201213 1week.lif - ctrl6-1_s.tif]

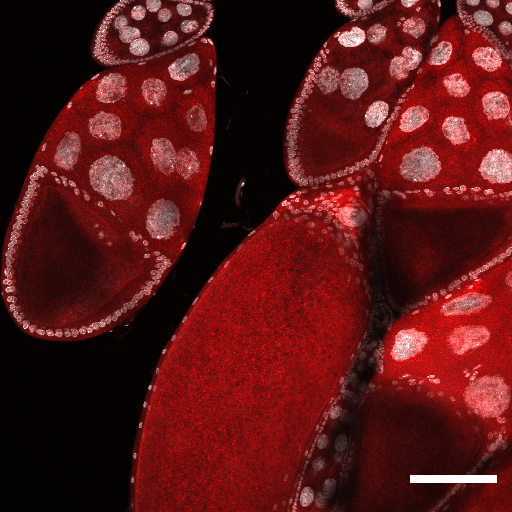

Supplement: S1 Raw Data — (ZIP) [file pgen.1011186.s001.zip › Poidevin DATA/Fig3/B2_MAX_20201213_1week.lif - ri453_.tif]

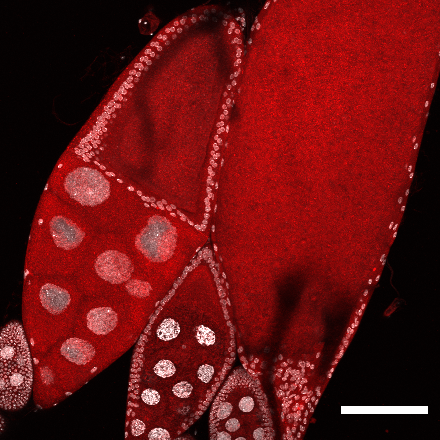

Supplement: S1 Raw Data — (ZIP) [file pgen.1011186.s001.zip › Poidevin DATA/Fig3/B3_MAX_20201213_1week.lif - ri887-1_.tif]

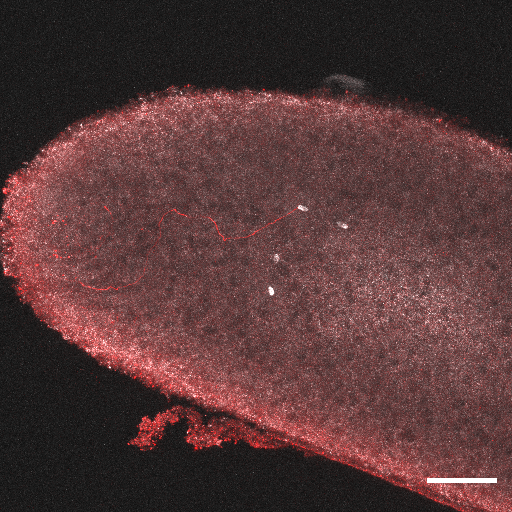

Supplement: S1 Raw Data — (ZIP) [file pgen.1011186.s001.zip › Poidevin DATA/Fig4/C_2020 07 1_Ovary_tub Acetyled _Cy3.lif_W.tif]

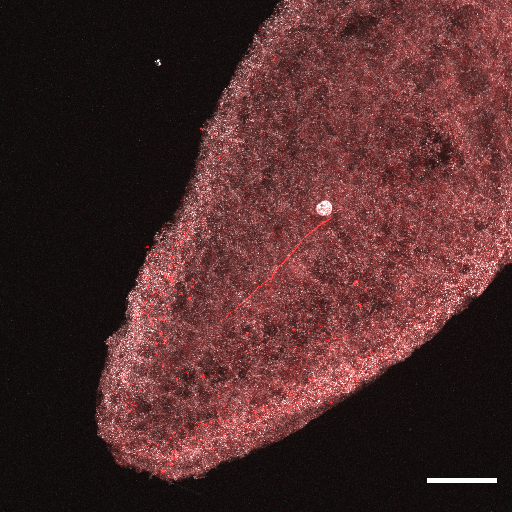

Supplement: S1 Raw Data — (ZIP) [file pgen.1011186.s001.zip › Poidevin DATA/Fig4/D_2020 07 1_Ovary _tub Acetyled _Cy3.lif_Ri5.tif]

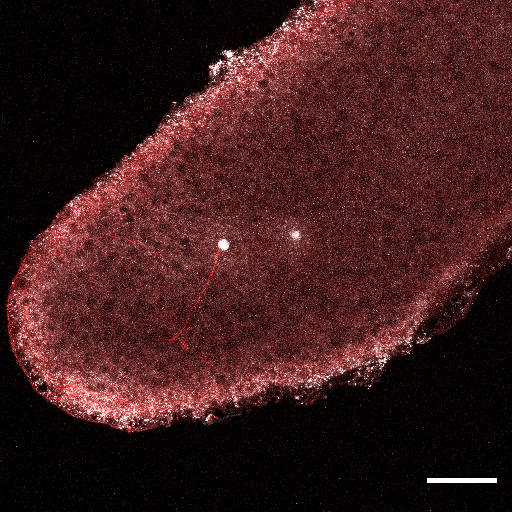

Supplement: S1 Raw Data — (ZIP) [file pgen.1011186.s001.zip › Poidevin DATA/Fig4/E_2020 07 1_Ovary_tub Acetyled _Cy3.lif_Ri88.tif]

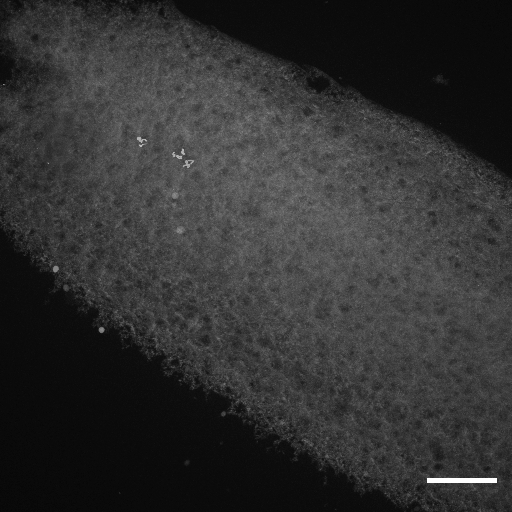

Supplement: S1 Raw Data — (ZIP) [file pgen.1011186.s001.zip › Poidevin DATA/Fig5/A_2020 09 16_Ovary _DAPI.lif - W.tif]

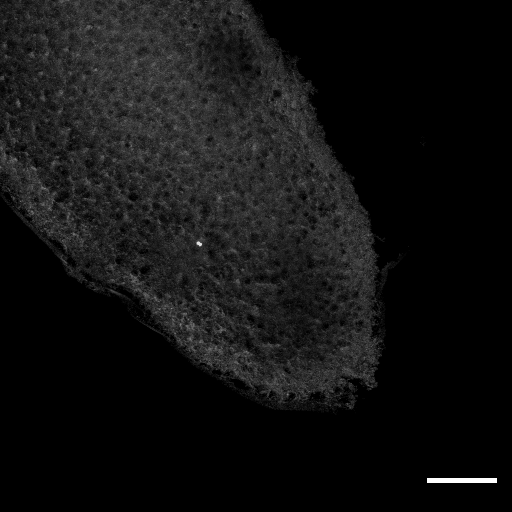

Supplement: S1 Raw Data — (ZIP) [file pgen.1011186.s001.zip › Poidevin DATA/Fig5/B_2020 09 16_Ovary_DAPI.lif_Ri5_9.tif]

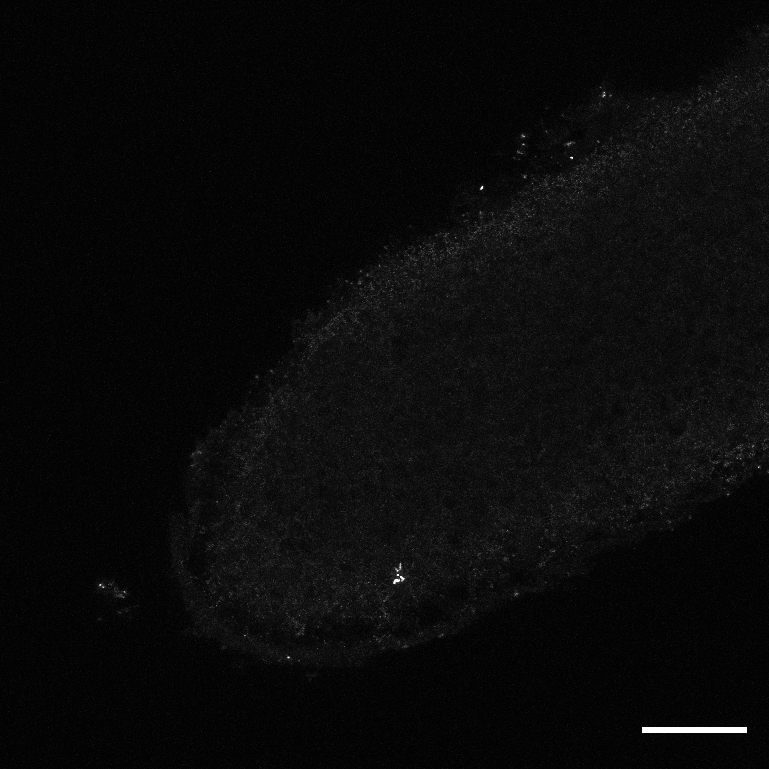

Supplement: S1 Raw Data — (ZIP) [file pgen.1011186.s001.zip › Poidevin DATA/Fig5/C_2020 09 16_Ovary_DAPI.lif_Ri88_16.tif]

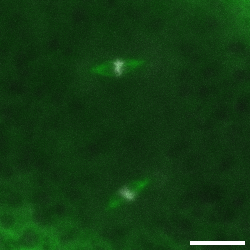

Supplement: S1 Raw Data — (ZIP) [file pgen.1011186.s001.zip › Poidevin DATA/Fig5/G_20230608_spindles oocyte_5.lif - F_U24_3.tif]

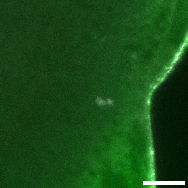

Supplement: S1 Raw Data — (ZIP) [file pgen.1011186.s001.zip › Poidevin DATA/Fig5/H_20230608_spindles oocyte_3.lif - F_R5_3.tif]

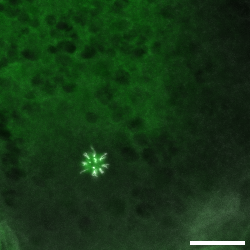

Supplement: S1 Raw Data — (ZIP) [file pgen.1011186.s001.zip › Poidevin DATA/Fig5/I_20230608_spindles oocyte_4.lif - F_Ri88_3.tif]

## Slide 1
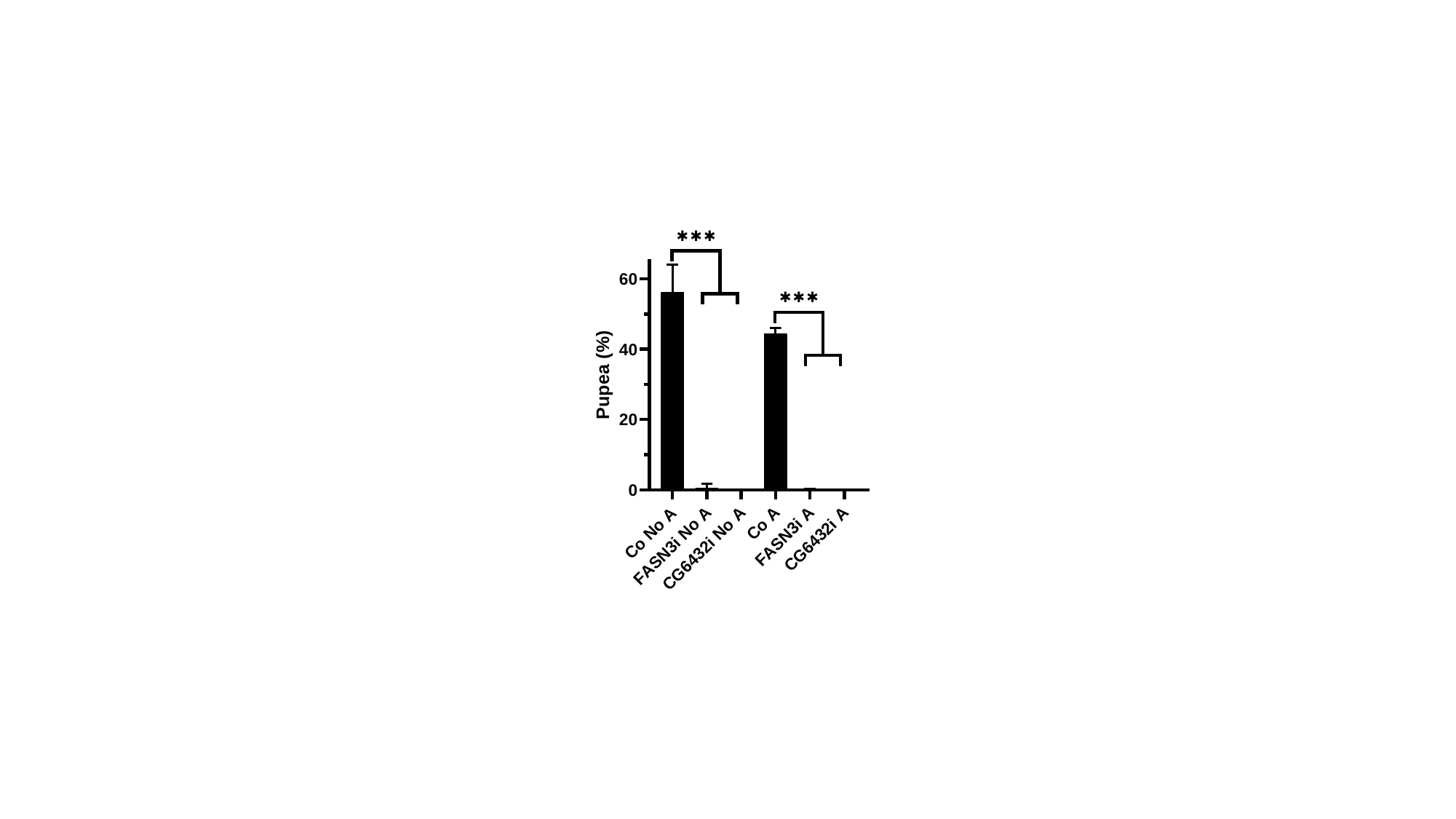

Supplement: S1 Raw Data — (ZIP) [file pgen.1011186.s001.zip › Poidevin DATA/SUP DATA/Supplementary figure S7/221130 Lay eggs Activation with or not dechorionation.pptx]

A

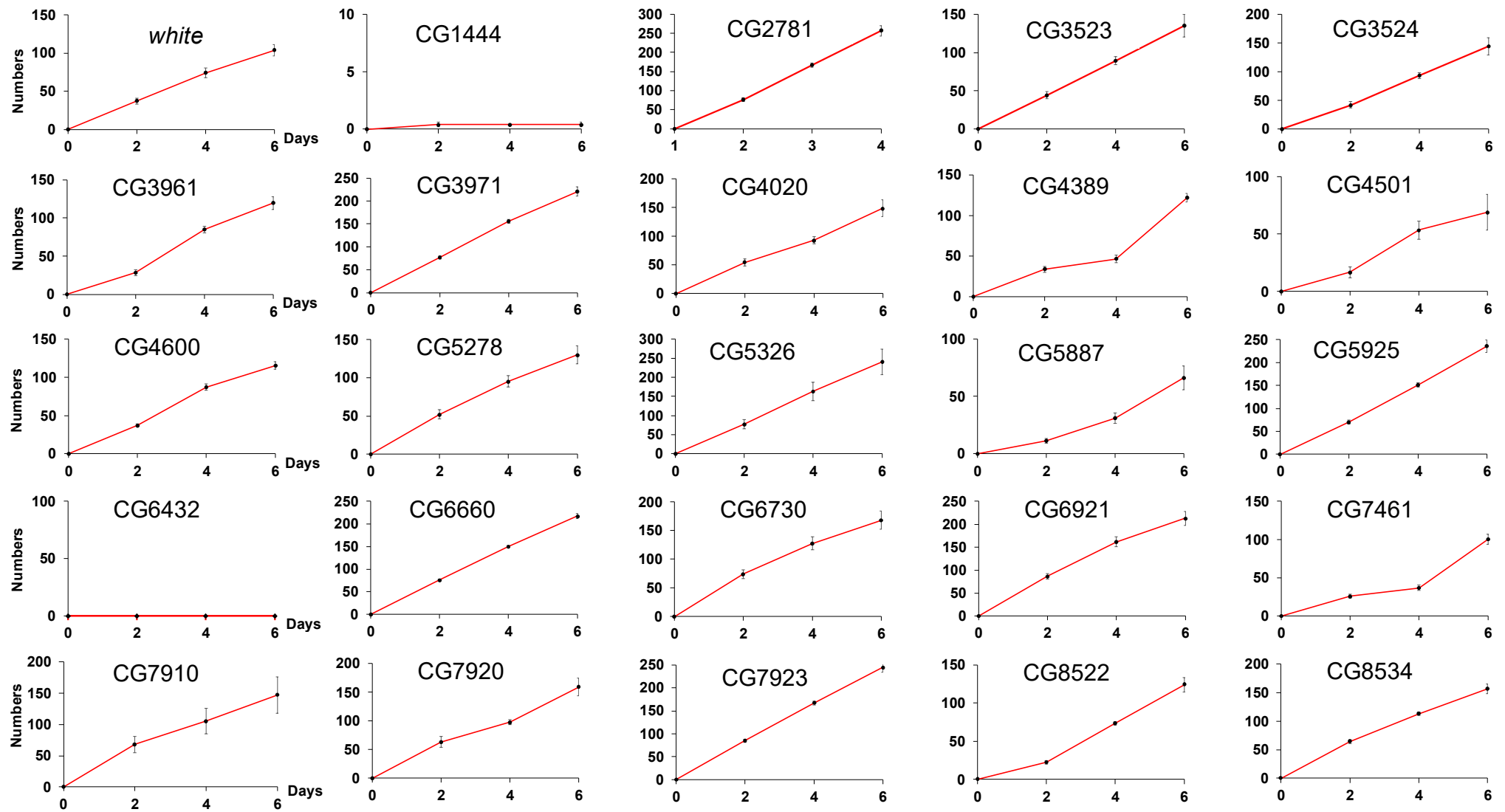

**A** continued

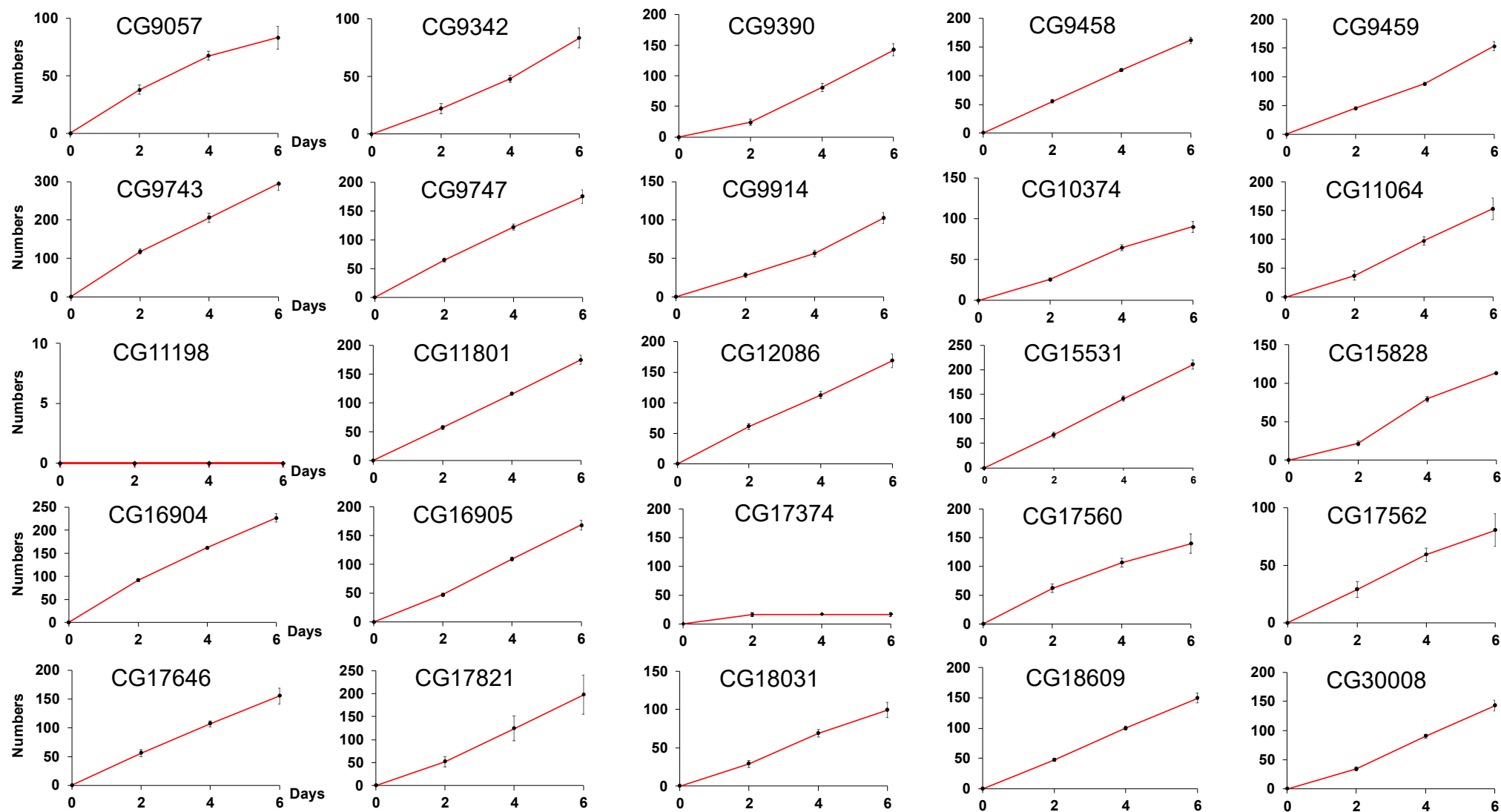

**A** continued

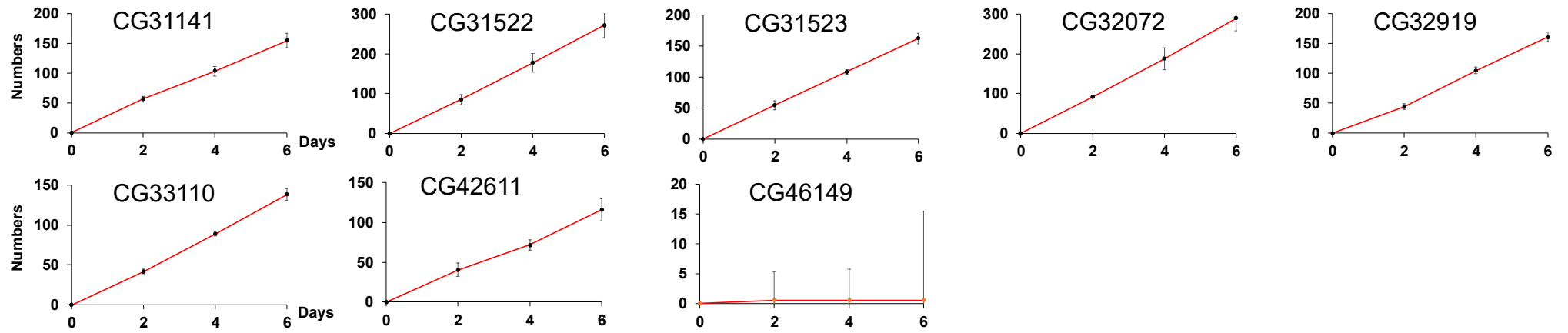

**B**

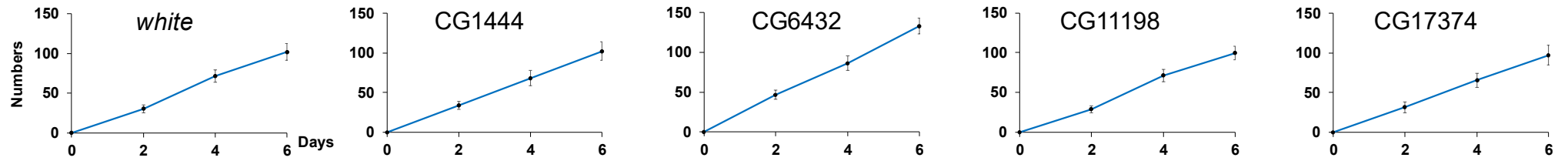

Supplement: S1 Fig — (A) 1407-gal4>UAS-RNAi females crossed to Canton-S males were let to lay eggs during six days (D) in three successive vials and the progeny was counted at adult emergence. The y-axis indicates total numbers (cumulative) of emerging flies after 2, 4 and 6 days. For each genotype, values are mean of emerging flies from 20 females maintained in separate tubes. (B) Reciprocal crosses to test male fertility. (PDF) [file pgen.1011186.s002.pdf]

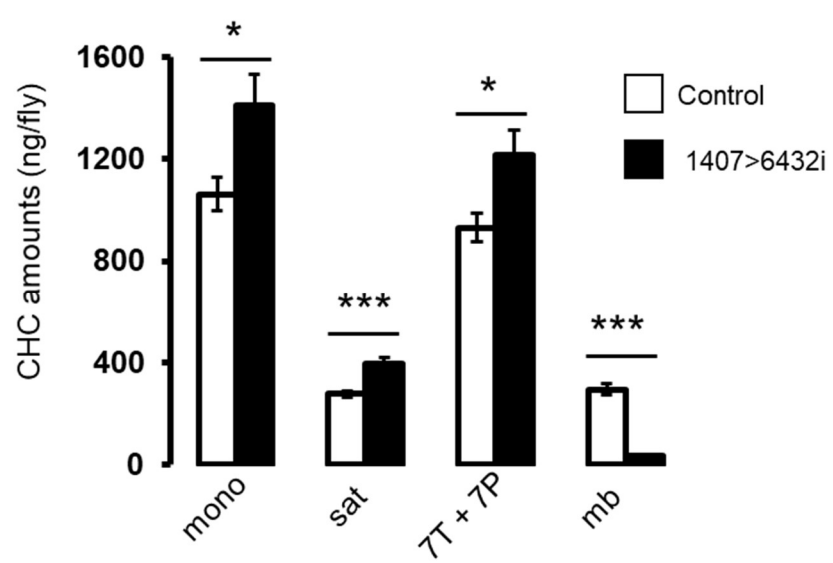

Supplement: S4 Fig — Means values of CHCs from 10 control (white) or 10 1407-gal4>CG6432-RNAi (black) males. Note the drop of mbCHCs that is compensated by an increase in linear CHCs in CG6432 knockdown males. (PDF) [file pgen.1011186.s005.pdf]

**A**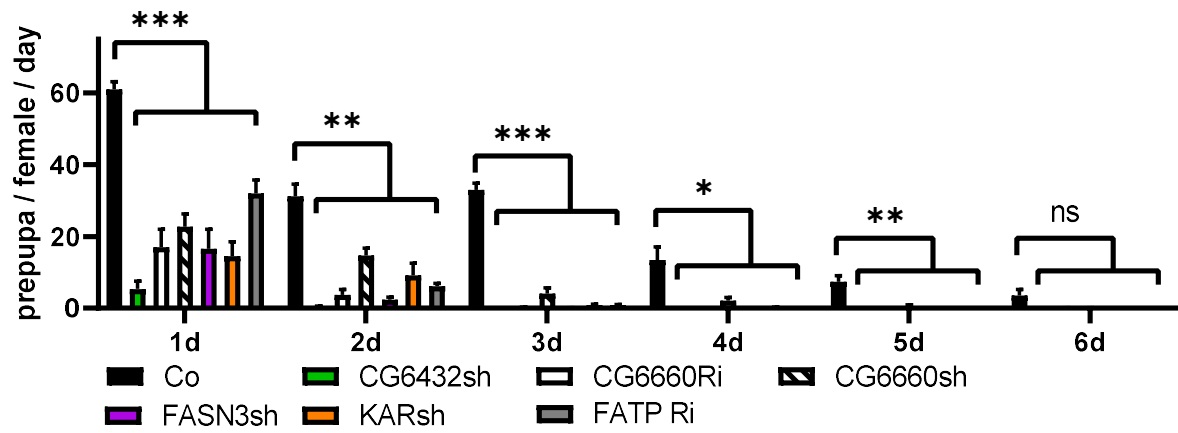**B**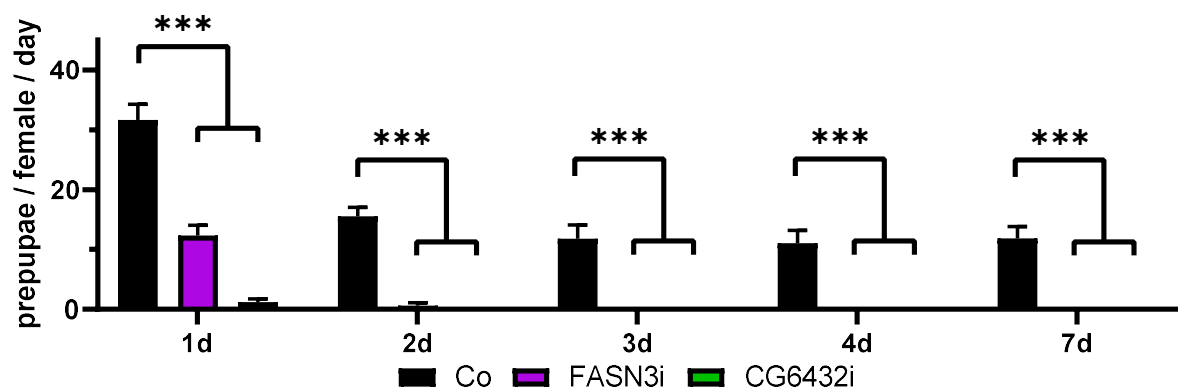**C**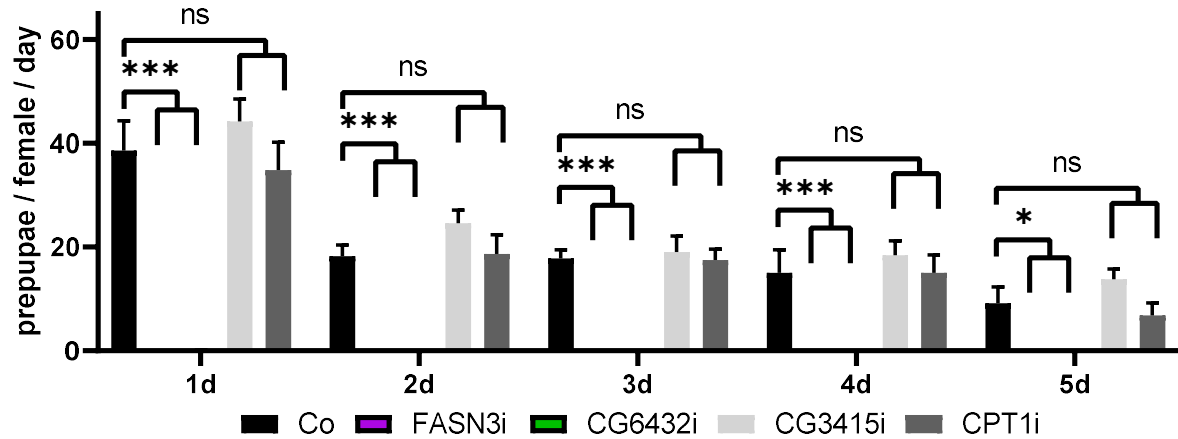**D**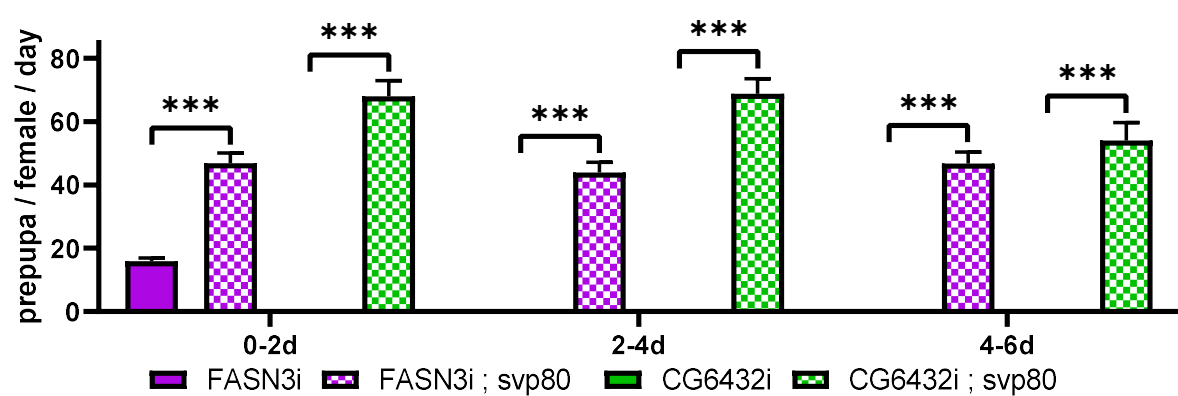

Supplement: S5 Fig — Fertility tests: (A) Testing additional lines directing either double strand RNA (Ri) or short-hairpin RNA (sh): pupal progeny of promE-gal4 females either control (black) or expressing CG6432-shRNA (green), eloCG6660-RNAi (white) eloCG6660-shRNA (dashed), FASN3-shRNA (purple), KAR/spidey-shRNA (orange) or FATP-RNAi (dark grey); developing animals were switched to 27°C at early metamorphosis, 3 adult females were mated to wild type males 3–5 days after adult eclosion; males were removed the day after and females were transferred to new vials every day. (B) Pupal progeny of 1407-gal4 females either control (black) or expressing FASN3-RNAi (purple) or CG6432-RNAi (green); 3-day old females were mated to wild type males; males were removed the day after and females were transferred to new vials every day for offspring counting. (C) Pupal progeny of promE-gal4 females either control (black) or expressing FASN3-RNAi (purple), CG6432-RNAi (green), CG3415-RNAi (light grey) or CPT1-RNAi (dark grey); developing animals were switched to 27°C at L2/L3 transition, adult females were mated to wild type males 4 days after adult eclosion and transferred to new vials every day; day 1 (1d) corresponds to the eggs laid by 5-day old females. (D) Adult progeny of 1407-gal4 females expressing either FASN3-RNAi (purple) or CG6432-RNAi (green), with (dotted colors) or without (plain colors) the svp-gal80 transgene; 3-4-day old females were mated to wild type males; males were removed the day after and females were transferred to new vials every days for offspring counting. Bars correspond to the mean values of pupae (5 replicates with 3 females each in A and C) or adults (20 females individually tested in B and D) obtained from each egg collection. (PDF) [file pgen.1011186.s006.pdf]

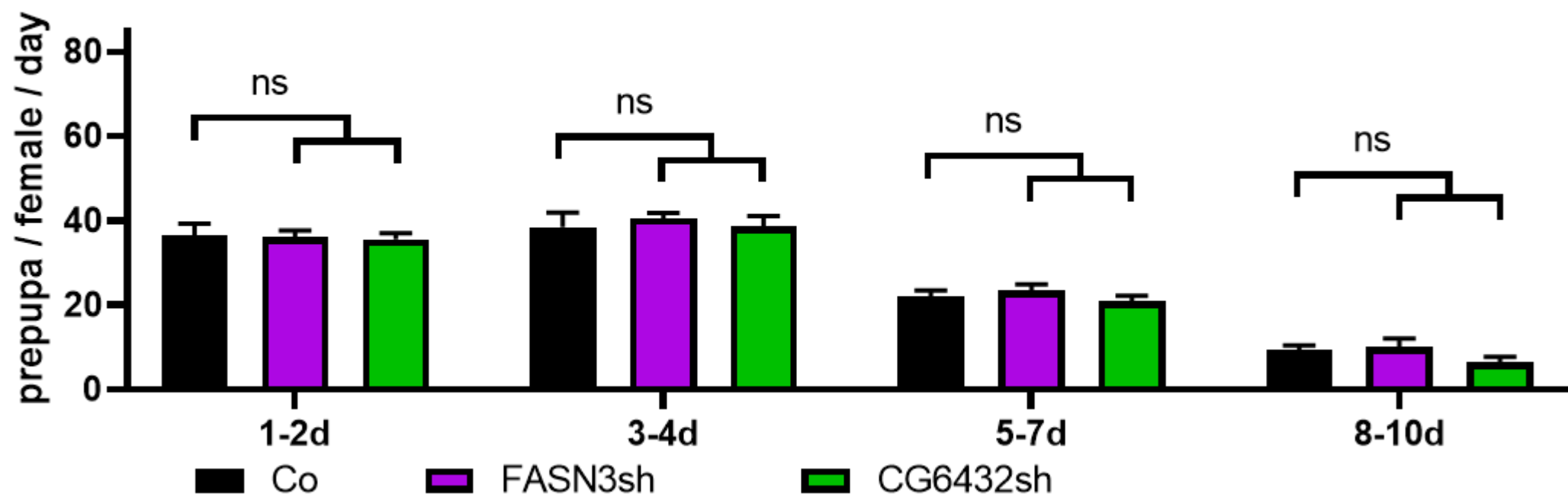

Supplement: S6 Fig — nanos-gal4 6-days old females either control (black) or expressing FASN3-sh (purple), CG6432-sh (green) were transferred every second or third days in new vials. Total pupal progeny is shown for each collection but calculated for one day. Females were maintained at 27°C since L3 early pupal stages and mated with wild type males during 6 days. Next, three females were transferred in new tubes as indicated. Mean values are obtained from six replicates (3 females each) for control and seven replicates for FASN3-sh and CG6432-sh. (PDF) [file pgen.1011186.s007.pdf]

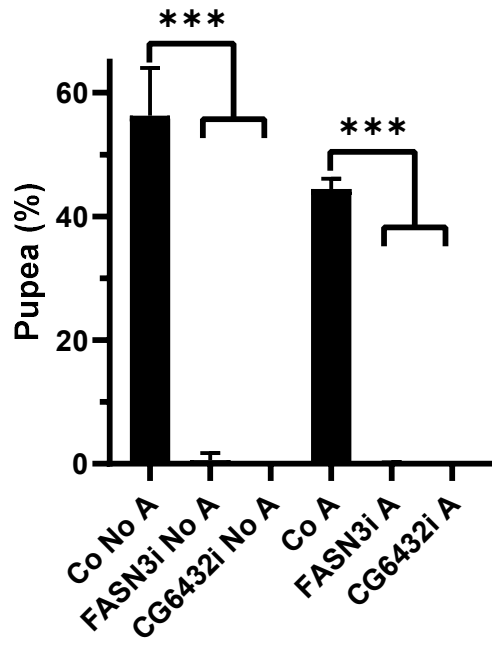

Supplement: S7 Fig — Pupal progeny from dechorionated eggs laid by promE-gal4 10-day old females either control (Co) or expressing FASN3-RNAi or CG6432-RNAi, with (A) or without (No A) in vitro activation. (PDF) [file pgen.1011186.s008.pdf]
