# Supplementary material for: A fatty acid anabolic pathway in specialized-cells sustains a remote signal that controls egg activation in Drosophila
Source: PLoS Genet. 2024 Mar 14;20(3):e1011186. doi: 10.1371/journal.pgen.1011186 (PMC10965083; doi:10.1371/journal.pgen.1011186)
Supplement: S3 Fig — Peptidic sequence alignment of CG6432 (Dm) to the best homologues Acss3 in mouse (Mm) and Acs1 in the yeast Saccharomyces cerevisiae (Sc), using www.uniprot.org. (PDF) [file pgen.1011186.s004.pdf]

```
Dm  -----------MEPGPSAVN------------------YEGS-----------------  13
Mm  MKPSWLQCRKVTGAGTLGAP-------------------LPGSPSVRGAAVTRRALVAGF  41
Sc  MSPSAVQSSKLEEQSSEIDKLKAKMSQSAATAQQKKEHEYEHLTSVK--IVPQRPISDRL  58
                  .

Dm  -ESV--CGMPVEAHDPLYLKAYRQSVQNPAAFWEEQG-NLLDWDRPWEKVLDNSN-----  64
Mm  GGRG--CRALTTGSGGEYKTHFAASVADPERFWGKAA-EQISWYKPWTKTLESRY-----  93
Sc  QPAIATHYSPHLDGLQDYQRLHKESIEDPAKFFGSKATQFLNWSKPFDKVFIPDPKTGRP  118
                     *   .  *: :*  *: . . : :.* :*: *.:

Dm  -PPFTKWYVGGYLNACYNSIDRHILAGRGSNVALIHDSPLTGTLRRVTYQELYDQIVLLA  123
Mm  -PPSTSWFVEGMLNICYNAIDRHIENGQGDKIAIIYDSPVTDTKATISYKEVLEQVSKLA  152
Sc  SFQNNAWFLNGQLNACYNCVDRHALKTPN-KKAIIFEGDEPGQGYSITYKELLEEVCQVA  177
        . *:: * ** ***.:***     . : *:*.:.   .   ::*:*: :::  :*

Dm  GGLA-KLGVVKGDRVVIYMPLIPETIIAMLAIVRLGAIHSVVFGGFAARELCSRIEHVEP  182
Mm  GVLV-KQGVKKGDTVVIYMPMIPQAIYTMLACARIGAIHSLIFGGFASKELSTRIDHAKP  211
Sc  QVLTYSMGVRKGDTVAVYMPMVPEAIITLLAISRIGAIHSVVFAGFSSNSLRDRINDGDS  237
      *. . ** *** *.:***::*::* ::**  *:*****::*.**::..*  **:. .

Dm  KLVIASNVGVEPGKVVPYLDILHSAISMSRWRPPQRNIIFRRDNVSPDTTKLDPLTDVLW  242
Mm  KVVVTASFGIEPGRKVEYIPLLEEALRIGQHRPDR-VLIYSRPNMEK--VPLMSGRDLDW  268
Sc  KVVITTDESNRGGKVIETKRIVDDALRETP--GVRHVLVYRKTNNPS--VAFHAPRDLDW  293
    *:*:::. . . *: :    ::..*:        :  ::: : *     . :    *: *

Dm  SDILKMAEGERPIACVPIEANDPLYILYTSGTTDKPKGVLRTIGGHLVALVYTLRTLYGI  302
Mm  EEEMAK---AQSHDCVPVLSEHPLYILYTSGTTGLPKGVVRPTGGYAVMLNWTMSSIYGL  325
Sc  ATEKKKY--KTYYPCTPVDSEDPLFLLYTSGSTGAPKGVQHSTAGYLLGALLTMRYTFDT  351
                  *.*: ::.**::*****:*. **** :  .*: :    *:   :.

Dm  NPGHTWWAASDMGWVVGHSYICYGPLCLGATSVMYEGKPDRTPDPGQYFRIIDQYQVRSI  362
Mm  KPGEVWWAASDLGWVVGHSYICYGPLLHGNTTVLYEGKPVGTPDAGAYFRVLAEHGVAAL  385
Sc  HQEDVFFTAGDIGWITGHTYVVYGPLLYGCATLVFEGTPA-YPNYSRYWDIIDEHKVTQF  410
    :  ..:::*.*:**:.**:*: ****  *  :::::**.*   *: . *: :: :: *  :

Dm  FSVPTSFRVIRRADPDISYGRQYSMKSLRAIFIAGEHCDYETKSWIEKT---FKVPVLNH  419
Mm  FTAPTAIRAIRQQDPGAALGKQYSLTRFKTLFVAGERCDVETLEWSKKV---FRVPVLDH  442
Sc  YVAPTALRLLKRAG--DSYIENHSLKSLRCLGSVGEPIAAEVWEWYSEKIGKNEIPIVDT  468
    : .**::* ::: .   :  .::*:. :: :  .**    *. .* .:      .:*:::

Dm  WWQTETGSAVTATCLGFQQNLSPPTYSTGLPLMGYDVKILKADGSEAQ-TSEL-GRIALK  477
Mm  WWQTETGSPITASCIGLGNSKTPPPGQAGKCVPGYNVMILDDNMQKLK-ARSL-GNIVVK  500
Sc  YWQTESGSHLVTPLAGGVT--PMKPGSASFPFFGIDAVVLDPNTGEELNTSHAEGVLAVK  526
    :****:** :.:   *          .:.  . * :. :*. :  :   :    * :.:*

Dm  LPLPPGNMATLYKNEELFRTLYFQKFPGYYDTMDAGYKDERGYIFVTARDDDVINVAGHR  537
Mm  LPLPPGAFSGLWKNQEAFKHLYFEKFPGYYDTMDAGYMDEEGYLYVMSRVDDVINVAGHR  560
Sc  A-AWPSFARTIWKNHDRYLDTYLNPYPGYYFTGDGAAKDKDGYIWILGRVDDVVNVSGHR  585
        *.    ::**.: :   *:: :**** * *..  *: **::: .* ***:**:***

Dm  LSTSSLEDAVLRHPDVVDVAVFGVPEATKGQVPLCLYIPVENCKKTD------AKLSTEI  591
Mm  ISAGAIEESVLSHGTVADCAVVGKEDPLKGHVPLALCVLKKDVNASE------EQVLEEI  614
Sc  LSTAEIEAAIIEDPIVAECAVVGFNDDLTGQAVAAFVVLKNKSSWSTATDDELQDIKKHL  645
    :*:. :* ::: .  *.: **.*  :  .*:.  .: :  :. . :         .:  .:

Dm  IKLIRDVVGPIAAFRLVTSVNNLPRTRSGKTMRKAMADFARNERVVLP--ATIDDASVFI  649
Mm  VKHVRQSIGPVAAFRNAVFVKQLPKTRSGKIPRSTLSALVNGKPYKVT--PTIEDPSIFG  672
Sc  VFTVRKDIGPFAAPKLIILVDDLPKTRSGKIMRRILRKILAGESDQLGDVSTLSNPGIVR  705
    :  :*. :**.** :     *.:**:*****  *  :  :  .:   :    *:.: .:.

Dm  EIRRALNQLGYAMTAPDPIVAKLLD                                    674
Mm  HIEEVLKQAV---------------                                    682
Sc  HLIDSVKL-----------------                                    713
    .:   ::
```
