# Supplementary material for: A fatty acid anabolic pathway in specialized-cells sustains a remote signal that controls egg activation in Drosophila
Source: PLoS Genet. 2024 Mar 14;20(3):e1011186. doi: 10.1371/journal.pgen.1011186 (PMC10965083; doi:10.1371/journal.pgen.1011186)
Supplement: S1 Table — (A) List of the genes and the corresponding lines screened for female sterility (fertility column) using the 1407-gal4 driver. (B) List of the genes and the corresponding lines tested for female sterility using the prom-gal4 driver. RNAi and shRNA lines were provided by NIG, BDSC or VDRC; three of them have been generated in S Eaton, CWT or JM laboratories (Stock column). The lines from BDSC express shRNA. (C) List of the gal4 drivers (left column), their tissue specific expression (middle column), and their usage (right column) in the present study. (PDF) [file pgen.1011186.s009.pdf]

A

CG	Name, Expected function	Line number	Stock center	Fertility
1444	Ketoacyl-DH/VLCFA synthesis	40949	VDRC	Sterile
2781	Elongase, VLCFA synthesis	102543	VDRC	+
3523	FASN1, LCFA synthesis	29349	VDRC	+
3524	FASN2, LCFA synthesis	4290	VDRC	+
3961	LCFA-CoA ligase	37305	VDRC	+
3971	Baldspot, Elongase, VLCFA synthesis	47519	VDRC	+
4020	FA-CoA reductase	107095	VDRC	+
4389	Mtpα, b-oxidation	21845	VDRC	+
4501	bgm, LCFA-CoA ligase	105635	VDRC	+
4600	yip2, Thiolase, b-oxidation	26562	VDRC	+
5887	desat1, desaturase	104350	VDRC	+
5925	desat2, desaturase	103666	VDRC	+
6432	short chain acyl-CoA ligase	43451	VDRC	Sterile
6660	Elongase, VLCFA synthesis	101046	VDRC	+
7461	Acyl-CoA dehydrogenase, b-oxidation	7461R-4	NIG	+
7910	FA amide hydrolase	51546	VDRC	+
7920	Acetyl-CoA hydrolase	21577	VDRC	+
7923	Fad2, desatF, desaturase		CWT	+
8522	SREBP, Transcription factor	37640	VDRC	+
9057	Lsd-2, Lipid storage	40734	VDRC	+
9342	Mtp, Lipid transport	110414	VDRC	+
9390	AcCoAS, Acetate-CoA ligase	100281	VDRC	+
9458	Elongase, VLCFA synthesis	48702	VDRC	+
9459	Elongase, VLCFA synthesis	48905	VDRC	+
9914	3-hydroxyacyl-CoA dehydrogenase	106649	VDRC	+
10374	Lsd-1, Lipid storage	30844	VDRC	+
11064	apolpp, Lipid transport	100944	VDRC	+
11198	ACC, malonyl-CoA synthesis	8105	VDRC	Sterile
12086	cue, LDL receptor	104645	VDRC	+
15531	FA desaturase	1397	VDRC	+
15828	Apoltp, Lipid transport		S Eaton	+
16904	Elongase, VLCFA synthesis	106515	VDRC	+
16905	eloF, VLCFA synthesis	16905-R1	NIG	+
17374	FASN3, LCFA synthesis		JM	Sterile
17560	FA-CoA reductase	104756	VDRC	+
17562	FA-CoA reductase	37365	VDRC	+
17646	ABC transporter-like	100378	VDRC	+
18031	FarO, FA-CoA reductase	30220	VDRC	+
18609	Elongase, VLCFA synthesis	4994	VDRC	+
30008	Elongase, VLCFA synthesis	6760	VDRC	+
31523	Elongase, VLCFA synthesis	45226	VDRC	+
10096	FA-CoA reductase	6090	VDRC	+
33110	Elongase, VLCFA synthesis	6926-R2	NIG	+
42611	mgl, LDL receptor	105071	VDRC	+
46149	Fatp, lipid transport, FA-CoA ligase	9406	VDRC	Sterile

B

CG	Name, Expected function	Line number	Stock center	Usage
1444	Ketoacyl-DH/VLCFA synthesis	40949	VDRC	Fig 1D
1444	Ketoacyl-DH/VLCFA synthesis	65013	BDSC	S5A Fig
3415	peroxisomal Multifunctional enzyme	108880	VDRC	S5C Fig
6432	short chain acyl-CoA ligase	43451	VDRC	Fig 1D
6432	short chain acyl-CoA ligase	58155	BDSC	S5A, S6 Fig
6660	Elongase, VLCFA synthesis	6660-R2	NIG	Fig 1D
6660	Elongase, VLCFA synthesis	101046	VDRC	S5A Fig
6660	Elongase, VLCFA synthesis	62422	BDSC	S5A Fig
11198	ACC, malonyl-CoA synthesis	8105	VDRC	Fig 1D
12891	carnitine O-palmitoyltransferase 1	105400	VDRC	S5C Fig
17374	FASN3, LCFA synthesis		JM	Fig 1D
17374	FASN3, LCFA synthesis	63026	BDSC	S5A S6 Fig
46149	Fatp, lipid transport, FA-CoA ligase	9406	VDRC	Fig 1D
46149	Fatp, lipid transport, FA-CoA ligase	100124	VDRC	S5A Fig

C

Driver	Speificity	Usage
1407-gal4	oencoytes and other tissues	Fig 1B; S1, S2, S4, S5B, S5D Fig
BO-gal4	oencoytes in embryo and L1	Fig 1A
promE-gal4	oenocytes from L1 to adult	Fig 1D; Fig 2-6, S5A, S5C, S7 Fig
Cg-gal4	fat body and hemocytes	Fig 1C
da-gal4	ubiquitous	Fig 1A
svp-gal80	gal4 repressor in oenocytes	Fig 2B, 2D, 2I; S5D Fig
Tub-gal80ts	thermozensitive gal4 repressor	Fig 1D; Fig 2-6, S5A, S5C, S7 Fig
nanos-gal4	germline	S6 Fig
