# Supplementary material for: A fatty acid anabolic pathway in specialized-cells sustains a remote signal that controls egg activation in Drosophila
Source: PLoS Genet. 2024 Mar 14;20(3):e1011186. doi: 10.1371/journal.pgen.1011186 (PMC10965083; doi:10.1371/journal.pgen.1011186)
Supplement: S2 Table — CHC amounts in 1407>CG6432-RNAi (6432i) flies: 4-5-day old males (top) or females (bottom). First column: CHC identities; elemental composition is indicated as the carbon chain length followed by the number of double bonds; Me- are mbCHCs. CHCs are expressed in ng/fly (Tot) or in percentages relative to total CHC amount as the mean (± SEM) of CHCs produced by 10 flies maintained 4 days at 25°C. (PDF) [file pgen.1011186.s010.pdf]

Males CHC	*P*	***6432i-C***	***6432i***
Tot	0.24	1634.1 ± 91.8	1840.3 ± 146.8
9-T	**0.003**	2.50 ± 0.25	3.78 ± 0.28
7-T	**0.002**	51.33 ± 1.05.2	55.7 ± 0.61
5-T	0.25	3.72 ± 0.18	4.14 ± 0.31
23 :0	**0.004**	13.54 ± 0.41	14.69 ± 0.30
Me-24	**<.001**	3.03 ± 0.24	0.38 ± 0.06
9-P	**0.003**	1.27 ± 0.14	2.24 ± 0.14
7-P	**<.001**	5.62 ± 0.19	10.24 ± 0.53
5-P	0.13	0.12 ± 0.07	0.34 ± 0.11
25 :0	0.50	1.85 ± 0.19	2.05 ± 0.22
Me-26	**<.001**	6.68 ± 0.43	0.42 ± 0.11
27 :0	**<.001**	1.20 ± 0.15	2.37 ± 0.21
Me-28	**<.001**	7.76 ± 0.61	0.62 ± 0.10
29 :0	**<.001**	0.47 ± 0.11	2.66 ± 0.24

Females CHC	*P*	***6432i-C***	***6432i***
Tot 23-29	0.26	1291.6 ± 64.7	1574.7 ± 236.1
9-T	0.25	0.70 ± 0.11	0.89 ± 0.11
7-T	0.75	3.32 ± 0.38	3.47 ± 0.29
5-T	**0.03**	0.26.± 0.06	0.60 ± 0.13
23 :0	0.05	11.22 ± 0.62	13.39 ± 0.84
7,11-PD	**0.04**	2.44 ± 0.30	1.68 ± 0.16
Me-24	**<.001**	1.88 ± 0.23	0.09 ± 0.01
9-P	1	3.14 ± 0.31	3.14 ± 0.37
7-P	0.96	2.07 ± 0.18	2.06 ± 0.19
5-P	0.42	0.16 ± 0.06	0.23 ± 0.06
25 :0	0.29	5.24 ± 0.28	5.83 ± 0.48
7,11-HD	**<.001**	18.84 ± 0.72	13.84 ± 0.56
Me-26	**<.001**	16.37 ± 0.57	0.60 ± 0.14
9-H	0.05	3.12 ± 0.14	2.56 ± 0.23
7-H	**0.01**	2.20 ± 0.16	2.90 ± 0.18
5-H	**0.01**	0.05 ± 0.02	0.19 ± 0.05
27 :0	**<.001**	2.78 ± 0.23	10.17 ± 0.44
7,11-ND	**<.001**	14.82 ± 0.52	28.95 ± 1.55
Me-28	**<.001**	9.23 ± 0.42	1.37 ± 0.34
9-N	0.11	0.19 ± 0.07	0.40 ± 0.10
7-N	0.06	0.22 ± 0.08	0.44 ± 0.08
29 :0	**<.001**	0.65 ± 0.07	6.50 ± 0.55
