# Supplementary material for: A fatty acid anabolic pathway in specialized-cells sustains a remote signal that controls egg activation in Drosophila
Source: PLoS Genet. 2024 Mar 14;20(3):e1011186. doi: 10.1371/journal.pgen.1011186 (PMC10965083; doi:10.1371/journal.pgen.1011186)
Supplement: S3 Table — CHC amounts in 1407-gal4> eloCG6660-RNAi (6660i) flies: 4-5-day old males (top) or females (bottom). First column: CHC identities; elemental composition is indicated as the carbon chain length followed by the number of double bonds; Abbreviation as in S2 Table. (PDF) [file pgen.1011186.s011.pdf]

**Male CHCs**	***P***	***6660i-C***	***6660i***
Tot 23-29	0.67	1325.4 ± 81.9	1393.2 ± 104.6
9-T	0.05	2.02 ± 0.13	1.79 ± 0.11
7-T	0.20	41.27 ± 1.92	47.46 ± 2.60
5-T	0.20	3.65 ± 0.30	4.65 ± 0.42
23 :0	0.22	10.71 ± 1.02	9.52 ± 0.28
Me-24	**0.01**	4.77 ± 0.30	5.95 ± 0.48
9-P	**0.01**	4.38 ± 0.46	2.10 ± 0.10
7-P	**0.02**	9.48 ± 0.58	10.24 ± 0.53
5-P	0.18	0.43 ± 0.11	0.26 ± 0.05
25 :0	0.55	1.92 ± 0.19	1.49 ± 0.53
Me-26	0.85	10.75 ± 0.54	11.22 ± 1.87
27 :0	0.97	0.96 ± 0.15	0.97 ± 0.46
Me-28	0.33	8.42 ± 1.34	7.22 ± 0.49
29 :0	0.43	0.28 ± 0.08	0.40 ± 0.12

**Female CHCs**	***P***	***6660i-C***	***6660i***
Tot 23-29	0.29	2080.4 ± 85.0	2388.4 ± 255.1
9-T	0.38	0.20 ± 0.03	0.16 ± 0.03
7-T	0.08	1.55 ± 0.21	1.09 ± 0.07
5-T	0.57	0.10.± 0.02	0.09 ± 0.02
23 :0	0.36	3.94 ± 0.34	4.43 ± 0.18
7,11-PD	0.08	2.66 ± 0.57	1.67 ± 0.18
Me-24	0.17	4.76 ± 0.38	3.90 ± 0.23
9-P	0.32	4.48 ± 0.32	5.54 ± 0.70
7-P	0.30	3.24 ± 0.41	2.71 ± 0.13
5-P	0.23	0.22 ± 0.03	0.16 ± 0.01
25 :0	0.26	3.43 ± 0.22	3.82 ± 0.18
7,11-HD	**0.01**	12.55 ± 1.10	9.69 ± 0.61
Me-26	0.77	31.20 ± 0.76	31.71 ± 0.93
9-H	0.23	4.13 ± 0.27	5.24 ± 0.59
7-H	0.79	2.34 ± 0.18	2.46 ± 0.30
5-H	0.54	0.12 ± 0.01	0.15 ± 0.04
27 :0	0.23	2.26 ± 0.17	2.69 ± 0.22
7,11-ND	0.07	6.36 ± 0.90	4.79 ± 0.30
Me-28	0.11	12.91 ± 1.19	16.53 ± 0.96
9-N	0.88	0.92 ± 0.16	0.88 ± 0.15
7-N	0.35	0.38 ± 0.07	0.32 ± 0.04
29 :0	0.93	0.54 ± 0.10	0.53 ± 0.13
