# Supplementary material for: A fatty acid anabolic pathway in specialized-cells sustains a remote signal that controls egg activation in Drosophila
Source: PLoS Genet. 2024 Mar 14;20(3):e1011186. doi: 10.1371/journal.pgen.1011186 (PMC10965083; doi:10.1371/journal.pgen.1011186)
Supplement: S1 Raw Data — (ZIP) [file pgen.1011186.s001.zip › Poidevin DATA/SUP DATA/Supplementary figure S5/S5 Fig Sup regroup.doc]

S5 Fig

5A, additional RNAi lines with promE (**old S5 Fig**)

5B, few early progeny with 1407-gal4 (**Claude**)

5C, promE starting 5days post eclosion: FASN3, CG6432, CG3415 (Ri-13) CG12891 (Ri-117) (**new Jacques & Delphine**)

5D, svp-gal4 with FASN3 and CG6432 (**Claude**)
